# Global burden of four cardiovascular diseases attributable to low fruits and vegetables intake, 1990–2021, with a forecast to 2044

**DOI:** 10.3389/fnut.2025.1655651

**Published:** 2025-10-02

**Authors:** Xin He, JiaXin Huang, Hua Cao, Zhangbo Cheng

**Affiliations:** ^1^Shengli Clinical Medical College, Fujian Medical University, Fuzhou, China; ^2^Department of Cardiovascular Surgery, Fuzhou University Affiliated Provincial Hospital, Fuzhou, China

**Keywords:** cardiovascular diseases, fruits and vegetables intake, dietary risk factors, global burden of disease, mortality trends, Socio-demographic Index (SDI)

## Abstract

**Background:**

Cardiovascular diseases (CVDs) remain the leading global cause of death. The contribution of inadequate fruits and vegetables (F&V) intake to specific CVD subtypes has not been well characterized across places and time.

**Methods:**

Using Global Burden of Disease (GBD) 2021 estimates for 204 countries/territories, we have quantified deaths and disability-adjusted life years (DALYs) from ischaemic heart disease (IHD), hypertensive heart disease (HHD), peripheral artery disease (PAD), and aortic aneurysm (AA) attributable to low fruit or low vegetables intake (1990–2021). We have assessed age-standardized mortality and DALY rates (ASMR, ASDR) and temporal trends via estimated annual percentage change (EAPC), examined patterns by age, sex, and Sociodemographic Index (SDI), decomposed changes into demographics versus epidemiologic effects, and generated forecasts to 2044 using Nordpred.

**Results:**

In 2021, inadequate F&V intake accounted for a substantial CVD burden worldwide. IHD and HHD dominated deaths and DALYs. Although ASMRs and ASDRs generally declined, absolute deaths rose, driven largely by population aging. AA, PAD, and IHD burdens were higher in men, whereas HHD burden was higher in women. Middle- and low-SDI settings carried greater IHD and HHD burdens, while AA and PAD were more prominent in high-SDI regions. Notably, HHD mortality attributable to low fruits intake increased across all SDI strata. Decomposition analyses indicated aging as the principal driver of rising deaths in high-SDI regions and combined effects of population growth and adverse epidemiologic change in lower-SDI regions. Projections suggested continued increases in deaths with relatively stable ASMRs through 2044.

**Conclusion:**

Despite improvements in age-standardized rates, persistent dietary risks and demographic aging sustained a high, uneven burden of diet-related CVDs. Equitable, population-level policies to improve F&V affordability and access–particularly in lower-SDI regions–are essential to curb future cardiovascular mortality.

## 1 Introduction

Cardiovascular diseases (CVDs) are the leading cause of death and disability worldwide, responsible for nearly 18 million deaths in 2021 alone (1, 2). Despite improvements in prevention and treatment, the global CVDs burden remains high, especially in low- and middle-income countries. As populations age and lifestyles change, the role of modifiable risk factors becomes increasingly important in shaping long-term cardiovascular health (3, 4).

Among dietary risks, low intake of fruits and vegetables (F&V) is a well-established contributor to cardiovascular morbidity (5, 6). These foods are rich in fiber, potassium, and antioxidants, which support vascular health and blood pressure regulation (7, 8). However, despite widespread recognition of their importance, global surveys indicate that three-quarters of the population still falls short of recommended intake levels, with striking disparities–low- and middle-income countries (LMICs) consume merely two-thirds of the WHO-recommended 400 g daily, particularly in sub-Saharan Africa and Southeast Asia where affordability and supply chain barriers persist (9). Global data on the mortality burden of CVDs attributable to insufficient F&V consumption remain limited.

Most previous studies have focused on the overall impact of fruits and vegetables intake on total CVDs, with limited attention to specific disease subtypes. In contrast, this study provides a comprehensive analysis of the effects of insufficient fruits and vegetables intake on four major CVD subtypes: ischaemic heart disease (IHD), hypertensive heart disease (HHD), peripheral artery disease (PAD), and aortic aneurysm (AA). Moreover, the differential impact of inadequate intake across regions and disease subtypes is demonstrated. Through detailed analyses stratified by gender, age, and spatiotemporal factors, the findings of this study offer new insights into the global burden of CVDs and provide a basis for more targeted dietary interventions (10–13). Importantly, few analyses have jointly assessed the contribution of low F&V intake to mortality from the four major CVDs: IHD, HHD, PAD, and AA. This gap limits the ability of policymakers to design targeted dietary interventions and prioritize prevention strategies based on specific disease patterns and population vulnerabilities.

This study utilized data from the Global Burden of Disease (GBD) Study 2021 to quantify global, regional, and national mortality and disability-adjusted life years (DALYs) from four major cardiovascular diseases (CVDs) – ischaemic heart disease (IHD), hypertensive heart disease (HHD), peripheral artery disease (PAD), and aortic aneurysm (AA) – attributable to low intake of fruits and vegetables (F&V) between 1990 and 2021. The data were disaggregated by age, sex, and the Sociodemographic Development Index (SDI) to provide a comprehensive analysis of how sociodemographic factors influence disease burden (14, 15). Temporal trends were assessed using the estimated annual percentage change (EAPC), allowing for the evaluation of changes over time. For long-term projections to 2044, the study employed the Nordpred model, a widely recognized and robust forecasting method specifically designed for projecting mortality and disease burden based on historical data. Nordpred was chosen over other time-series models due to its proven ability to handle age-standardized rates and account for population growth and aging, making it highly suitable for forecasting complex disease trends. The model’s validation in several global health studies and its capacity to provide reliable projections for CVDs across different regions and demographics further strengthen its appropriateness for this analysis. Through the use of Nordpred, this study aims to provide a granular understanding of diet-related CVD mortality, inform equitable nutrition policies, and support global efforts to reduce cardiovascular risks through population-level dietary improvements.

## 2 Materials and methods

### 2.1 Data source, study population and setting

This study draws on data from the GBD Study 2021, which provides harmonized estimates of diseases and risk factors across 204 countries and territories (16, 17). It further extracted data on mortality and DALYs for four CVD subtypes–IHD, HHD, PAD, and AA–attributable to low fruit and low vegetables intake between 1990 and 2021.

The GBD 2021 estimates–accessed via the Global Health Data Exchange (GHDx) platform^1^ –served as the foundation for evaluating the burden of CVDs attributable to insufficient F&V intake. Annual data on CVD-related mortality, DALYs, and age-standardized rates (ASRs) from 1990 to 2021 were extracted. Variable selection was aligned with the study objectives, focusing on relevant disease categories, dietary risk factors, and key demographic indicators. Methodological details have been comprehensively documented in previous GBD publications.

In this study, “low fruits intake” refers to daily intake of less than 340–350 g, based on the Theoretical Minimum-Risk Exposure Level (TMREL) for fruits intake, as defined by the Global Burden of Disease (GBD) study [GBD 2021 Risk Factor Collaborators (18)]. These thresholds are grounded in a systematic review of global epidemiological studies, which identify this level of intake as being associated with the lowest risk for various diet-related diseases, including cardiovascular diseases [GBD 2021 Risk Factor Collaborators (18)]. This includes fresh, frozen, cooked, canned, and dried fruit, but excludes fruit juices and salted/pickled fruit. Similarly, “low vegetables intake” refers to daily intake of less than 306–372 g, also based on the TMREL for vegetables intake as per GBD 2021 [GBD 2021 Risk Factor Collaborators (18)]. This intake level was similarly derived from global dietary health studies, and is associated with optimal health outcomes. It includes non-starchy fresh, frozen, cooked, and dried vegetables, while excluding legumes, salted/pickled vegetables, juices, nuts/seeds, and starchy varieties like potatoes or corn (18).

These two risk factors were analyzed in relation to four major cardiovascular conditions: ischaemic heart disease (IHD; ICD-10: I20–I25), hypertensive heart disease (HHD; ICD-10: I11), lower extremity peripheral artery disease (PAD; ICD-10: I73.9), and aortic aneurysm (AA; ICD-10: I71). These diseases were identified through standardized diagnostic codes from hospital records, medical registries, and health system data. The GBD modeling framework ensured high-quality, comparable estimates of disease burden across diverse settings and time periods.

Mortality and DALYs were used as the primary outcomes, each reported with corresponding 95% uncertainty intervals (UIs). DALYs represent the overall health loss from a condition, combining years of life lost (YLLs) due to premature mortality with years lived with disability (YLDs). Details on the calculation of both YLLs and YLDs are provided in the Section “2.2 Statistical analysis” below. This composite measure captures both the fatal and non-fatal impacts of CVDs attributable to inadequate F&V intake.

To explore the role of socio-economic development in shaping disease burden, this study employed the Sociodemographic Index (SDI)–a composite metric based on lag-distributed income per capita, average educational attainment among individuals aged 15 years or older, and the total fertility rate among women younger than 25 years (19, 20). SDI scores ranged from 0 to 1 and were categorized into five groups for analysis: (1) low SDI (0–0.47), (2) low-middle SDI (0.47–0.62), (3) middle SDI (0.62–0.71), (4) high-middle SDI (0.71–0.81), and (5) high SDI (0.81–1.00). This classification allowed for stratified comparisons of CVD burden by development level, offering critical insight into how socio-economic context influences diet-related health outcomes globally.

### 2.2 Statistical analysis

The burden of CVDs attributable to inadequate F&V intake was evaluated using mortality rates (per 10,000 population), disability-adjusted life years (DALYs, per 100,000 population), age-standardized mortality rates (ASMR rates, per 100,000 population), age-standardized DALY rates (ASDR, per 100,000 population), and EAPC in ASMR and ASDR. Analyses were conducted across sexes, regions, and countries at global, regional, and national levels. DALYs provide a comprehensive measure of disease burden by combining YLLs due to premature mortality with YLDs. YLLs were calculated by multiplying the number of deaths by the difference between standard life expectancy and age at death, while YLDs were derived by multiplying the prevalence of disease sequelae by corresponding disability weights, which range from 0 (perfect health) to 1 (equivalent to death). To account for statistical uncertainty in the estimates, 95% UIs were reported for all metrics, defined as the range between the 2.5th and 97.5th percentiles of the posterior distribution, providing a credible range of plausible values for the burden of CVDs attributable to insufficient F&V consumption.

The age-standardized rate (ASR) for both ASMR and ASDR was calculated using the following formula:


$$A\,S\,R=\overset{n}{\underset{i=1}{\sum }}\frac{\left(E_{i}\times P_{i}\right)}{W_{i}}$$

E_i_ The total number of events in age group; P_i_ The total population of age group; W_i_ The weight of age group in the standard age distribution; *n* = The total number of age groups.

Age-standardized mortality rates were calculated using the GBD 2021 standard world population. ASMR rates allow for the comparison of mortality patterns across populations, regions, and time periods by accounting for differences in age distribution. Without such adjustment, direct comparisons may be confounded by demographic variation, potentially leading to biased or misleading interpretations. By removing the influence of age structure, ASMR facilitates more equitable comparisons of mortality rates. Similarly, ASDR, which incorporate both fatal and non-fatal health outcomes, provide a comparable metric for assessing overall disease burden across populations (21).

To evaluate temporal trends in the ASMR and ASDR of CVDs attributable to insufficient F&V consumption, this study estimated the EAPC from 1990 to 2021. The EAPC quantifies the average yearly change in age-standardized rates, enabling a clearer understanding of the trajectory and magnitude of changes in CVD mortality and disability burdens associated with suboptimal dietary intake over time and across regions.

An age–period–cohort (APC) model was employed to disentangle the effects of age, time period, and birth cohort on trends in ASMR and ASDR (22, 23). The age effect reflects both biological processes and social determinants associated with aging, such as physiological decline and shifts in social roles and exposures. For this analysis, individuals were stratified into 15 age groups: 25–29, 30–34, 35–39, 40–44, 45–49, 50–54, 55–59, 60–64, 65–69, 70–74, 75–79, 80–84, 85–89, 90–94, and ≥95 years. Analyses of IHD, HHD, and AA included all age groups, while PAD analyses were restricted to individuals aged 40 years and older.

To forecast the future burden of CVDs–including mortality, DALYs, ASMR, and ASDR–this study applied the Nordpred model, which accounts for temporal changes in population demographics, dietary patterns, and health system performance. All statistical analyses and visualizations were conducted using R (version 4.3.1) and Python (version 3.14), with statistical significance defined as a two-sided *p*-value < 0.05.

### 2.3 Patient and public involvement

The GBD database served as the foundation for this study. It is a widely respected resource that provides comprehensive data on various health conditions, risk factors, and mortality rates worldwide. Since the GBD data were anonymized and contained aggregated scores, this study focused on reorganizing and analyzing the information to advance public health research and inform health policy on cardiovascular diseases related to inadequate F&V intake. No individual patient data were involved in the design or analysis of the study.

## 3 Results

### 3.1 Global burden attributable to low F&V intake, 2021

**FIGURE 1:**
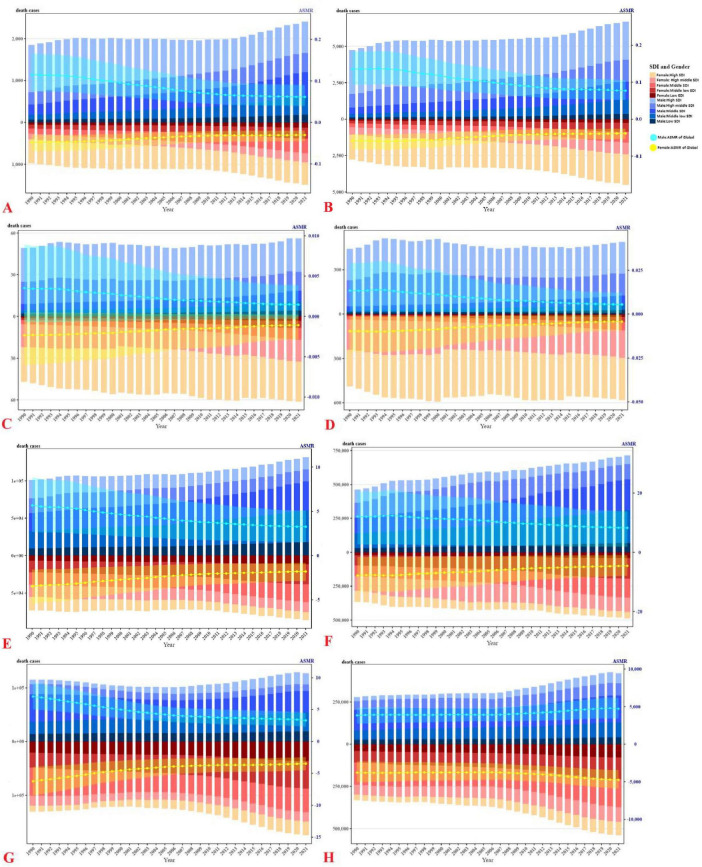
Death cases and ASMR of CVDs due to inadequate F&V intake (1990–2021), divided by gender, SDI levels, and region. **(A)** AA, **(C)** PAD, **(E)** IHD, **(G)** HHD, attributed to insufficient intake of vegetables; **(B)** AA, **(D)** PAD, **(F)** IHD, **(H)** HHD, attributed to insufficient intake of fruits; bar chart represents death cases, line chart represents ASMR.

From 1990 to 2021, inadequate F&V intake remained a persistent key contributor to the global burden of four major CVD subtypes (IHD, HHD, AA, PAD), with global age-standardized mortality rates (ASMRs) exhibiting an overall downward trajectory yet marked disparities across Socio-demographic Index (SDI) regions (Table 1 and Supplementary Table 1). IHD and HHD emerged as the two most burdensome subtypes: while their global ASMRs declined steadily over the 31-years period, low-SDI regions consistently maintained substantially higher ASMRs than high-SDI regions in both 1990 and 2021, reflecting enduring regional inequities in dietary risk mitigation (Table 1 and Supplementary Table 1). Detailed temporal trends in death cases and ASMRs, stratified by gender, SDI levels, and regional grouping, are illustrated in Figure 1.

**TABLE 1:** The ASMR of cardiovascular diseases related to inadequate F&V intake globally, by SDI categories and GBD regions, from 1990 to 2021.

Location	Insufficient fruits intake and CVDs 2021		Insufficient vegetables intake and CVDs 2021	
	AA-ASMR (95% UI)	AA-EAPC (95% UI)	AA-ASMR (95% UI)	AA-EAPC (95% UI)
Global	0.065 (0.044, 0.089)	−1.888 (−2.024, −1.753)	0.051 (0.034, 0.074)	−2.068 (−2.207, −1.929)
High SDI	0.098 (0.066, 0.134)	−2.805 (−2.962, −2.647)	0.064 (0.040, 0.096)	−3.518 (−3.693, −3.343)
High-middle SDI	0.058 (0.040, 0.080)	−1.236 (−1.431, −1.041)	0.038 (0.023, 0.057)	−1.065 (−1.223, −0.906)
Middle SDI	0.038 (0.025, 0.053)	−0.280 (−0.392, −0.167)	0.041 (0.027, 0.058)	−0.669 (−0.813, −0.525)
Middle-low SDI	0.061 (0.038, 0.093)	1.309 (1.238, 1.381)	0.057 (0.036, 0.088)	1.005 (0.936, 1.073)
Low SDI	0.068 (0.036, 0.114)	0.510 (0.354, 0.666)	0.072 (0.037, 0.125)	0.118 (−0.055, 0.290)
	PAD-ASMR (95% UI)	PAD-EAPC (95% UI)	PAD-ASMR (95% UI)	PAD-EAPC (95% UI)
Global	0.006 (0.001, 0.013)	−3.202 (−3.354, −3.050)	0.002 (−0.000, 0.004)	−2.909 (−3.038, −2.781)
High SDI	0.009 (0.001, 0.020)	−2.857 (−3.043, −2.671)	0.002 (−0.000, 0.006)	−2.812 (−3.062, −2.561)
High-middle SDI	0.008 (0.001, 0.018)	−3.944 (−4.155, −3.734)	0.002 (−0.000, 0.005)	−3.586 (−3.762, −3.410)
Middle SDI	0.002 (0.000, 0.005)	−1.216 (−1.298, −1.134)	0.001 (−0.000, 0.002)	−1.687 (−1.825, −1.549)
Middle-low SDI	0.003 (0.000, 0.007)	0.513 (0.424, 0.603)	0.001 (−0.000, 0.003)	0.339 (0.239, 0.440)
Low SDI	0.006 (0.001, 0.018)	0.584 (0.416, 0.753)	0.002 (−0.000, 0.007)	0.334 (0.131, 0.538)
	IHD-ASMR (95% UI)	IHD-EAPC (95% UI)	IHD-ASMR (95% UI)	IHD-EAPC (95% UI)
Global	6.976 (1.482, 11.851)	−1.627 (−1.707, −1.547)	2.771 (1.270, 4.398)	−2.099 (−2.185, −2.013)
High SDI	2.404 (0.510,4.321)	−3.961 (−4.103, −3.818)	1.363 (0.596, 2.213)	−3.808 (−3.905, −3.712)
High-middle SDI	5.493 (1.140, 9.871)	−3.114 (−3.553, −2.673)	1.715 (0.720, 2.940)	−3.395 (−3.605, −3.185)
Middle SDI	7.058 (1.484, 12.194)	−1.095 (−1.165, −1.025)	2.664 (1.199, 4.350)	−1.912 (−2.002, −1.822)
Middle-low SDI	13.849 (2.922, 23.121)	−0.095 (−0.193, 0.002)	5.197 (2.400, 8.056)	−0.761 (−0.860, −0.662)
Low SDI	11.436 (2.362, 19.250)	−0.155 (−0.260, −0.050)	6.505 (2.997, 10.076)	−0.760 (−0.851, −0.669)
	HHD-ASMR (95% UI)	HHD-EAPC (95% UI)	HHD-ASMR (95% UI)	HHD-EAPC (95% UI)
Global	5.557 (4.207, 6.902)	−1.361 (−1.500, −1.222)	4.090 (3.018, 5.241)	−1.959 (−2.155, −1.762)
High SDI	2.643 (1.957, 3.338)	0.810 (0.575, 1.046)	2.149 (1.516, 2.862)	0.980 (0.727, 1.233)
High-middle SDI	3.890 (2.735, 5.242)	−1.472 (−1.558, −1.386)	1.811 (1.168, 2.693)	−2.950 (−3.129, −2.771)
Middle SDI	6.859 (4.582, 9.484)	−2.781 (−3.049, −2.512)	4.225 (2.894, 5.912)	−4.095 (−4.394, −3.796)
Middle-low SDI	8.787 (6.693, 11.150)	−1.149 (−1.257, −1.042)	7.915 (5.805, 10.285)	−1.173 (−1.271, −1.074)
Low SDI	14.504 (10.229, 18.823)	−0.877 (−1.022, −0.731)	15.122 (10.580, 19.798)	−1.013 (−1.165, −0.861)

AA, aortic aneurysm; PAD, peripheral artery disease; IHD, ischaemic heart disease; HHD, hypertensive heart disease; ASMR, age-standardized mortality rate (per 100 000 population); EAPC, estimated annual percentage change for the preceding ASMR column; UI, uncertainty interval; F&V, fruits and vegetables; SDI, Socio-demographic Index; GBD, Global Burden of Disease; CVDs, cardiovascular diseases. All rates are expressed per 100 000 population.

Aortic aneurysm and PAD contributed far less to global F&V-attributable CVD mortality but exhibited striking regional heterogeneity. High-SDI regions experienced steeper declines in ASMRs for AA and PAD between 1990 and 2021, whereas middle-low and low SDI strata showed either stagnation or modest upticks in these rates (Table 1 and Supplementary Table 1). For metrics capturing non-fatal health impacts and overall health loss, trends in disability-adjusted life years (DALYs) and age-standardized DALY rates (ASDRs) for all four CVD subtypes–further disaggregated by gender, SDI, and region–are detailed in Table 2 and Supplementary Figure 1.

**TABLE 2:** The ASDR of cardiovascular diseases related to inadequate F&V intake globally, by SDI categories and GBD regions, from 1990 to 2021.

Location	Insufficient fruits intake and CVDs 2021		Insufficient vegetables intake and CVDs 2021	
	AA-ASDR (95% UI)	AA-EAPC (95% UI)	AA-ASDR (95% UI)	AA-EAPC (95% UI)
Global	1.361 (0.922, 1.854)	−1.711 (−1.840, −1.581)	1.051 (0.685, 1.523)	−1.857 (−1.998, −1.715)
High SDI	1.975 (1.346, 2.670)	−2.701 (−2.854, −2.548)	1.235 (0.773, 1.843)	−3.425 (−3.589, −3.261)
High-middle SDI	1.382 (0.940, 1.889)	−1.235 (−1.433, −1.036)	0.813 (0.503, 1.226)	−1.126 (−1.300, −0.952)
Middle SDI	0.866 (0.572, 1.196)	−0.225 (−0.337, −0.112)	0.861 (0.575, 1.236)	−0.736 (−0.884, −0.587)
Middle-low SDI	1.314 (0.828, 2.005)	1.221 (1.160, 1.283)	1.209 (0.765, 1.868)	0.949 (0.887, 1.011)
Low SDI	1.469 (0.773, 2.449)	0.458 (0.321, 0.595)	1.556 (0.789, 2.704)	0.078 (−0.080, 0.237)
	PAD-ASDR (95% UI)	PAD-EAPC (95% UI)	PAD-ASDR (95% UI)	PAD-EAPC (95% UI)
Global	0.148 (0.018, 0.335)	−2.788 (−2.918, −2.658)	0.035 (−0.007, 0.092)	−2.707 (−2.790, −2.624)
High SDI	0.207 (0.026, 0.459)	−2.620 (−2.751, −2.488)	0.050 (−0.010, 0.129)	−2.599 (−2.753, −2.446)
High-middle SDI	0.186 (0.023, 0.415)	−3.541 (−3.760, −3.321)	0.034 (−0.007, 0.089)	−3.458 (−3.632, −3.283)
Middle SDI	0.088 (0.010, 0.209)	−1.534 (−1.626, −1.442)	0.022 (−0.004, 0.058)	−2.436 (−2.596, −2.275)
Middle-low SDI	0.099 (0.011, 0.235)	−0.439 (−0.487, −0.390)	0.031 (−0.006, 0.084)	−0.502 (−0.538, −0.465)
Low SDI	0.154 (0.015, 0.404)	0.044 (−0.028, 0.116)	0.052 (−0.008, 0.152)	−0.185 (−0.298, −0.073)
	IHD-ASDR (95% UI)	IHD-EAPC (95%UI)	IHD-ASDR (95% UI)	IHD-EAPC (95% UI)
Global	184.188 (40.872, 304.211)	−1.522 (−1.606, −1.438)	70.624 (34.114, 109.099)	−1.897 (−2.000, −1.794)
High SDI	59.017 (13.073, 103.708)	−3.665 (−3.812, −3.518)	31.353 (14.441, 50.104)	−3.502 (−3.619, −3.384)
High-middle SDI	120.888 (26.434, 211.648)	−3.408 (−3.891, −2.922)	35.880 (15.853, 59.983)	−3.572 (−3.779, −3.365)
Middle SDI	180.215 (39.834, 301.519)	−1.212 (−1.264, −1.160)	66.216 (31.535, 104.277)	−1.893 (−2.020, −1.765)
Middle-low SDI	370.486 (80.670, 606.797)	−0.275 (−0.361, −0.189)	132.316 (63.832, 202.280)	−0.905 (−0.991, −0.818)
Low SDI	288.752 (61.353, 479.142)	−0.431 (−0.521, −0.341)	160.973 (76.842, 245.863)	−0.912 (−1.008, −0.817)
	HHD-ASDR (95% UI)	HHD-EAPC (95% UI)	HHD-ASDR (95% UI)	HHD-EAPC (95% UI)
Global	111.469 (86.141, 136.211)	−1.532 (−1.667, −1.397)	83.675 (62.764, 105.727)	−2.059 (−2.275, −1.842)
High SDI	59.693 (45.306, 74.565)	1.171 (0.939, 1.404)	47.015 (33.636, 62.427)	1.393 (1.120, 1.667)
High-middle SDI	65.306 (47.136, 86.877)	−2.062 (−2.139, −1.986)	27.941 (18.583, 40.650)	−3.810 (−4.028, −3.591)
Middle SDI	125.909 (87.741, 169.590)	−2.900 (−3.162, −2.638)	80.045 (56.786, 108.114)	−4.121 (−4.464, −3.777)
Middle-low SDI	174.185 (133.338, 218.880)	−1.302 (−1.397, −1.207)	154.690 (114.063, 199.380)	−1.323 (−1.408, −1.237)
Low SDI	287.536 (201.145, 375.999)	−1.134 (−1.256, −1.012)	298.165 (206.997, 393.288)	−1.268 (−1.399, −1.137)

AAA, aortic aneurysm; PAD, peripheral artery disease; IHD, ischaemic heart disease; HHD, hypertensive heart disease; ASDR, age-standardized DALY rate (per 100 000 population); EAPC, estimated annual percentage change for the preceding ASDR column; UI, uncertainty interval; F&V, fruits and vegetables; SDI, Socio-demographic Index; GBD, Global Burden of Disease; CVDs, cardiovascular diseases. All rates are expressed per 100 000 population.

### 3.2 National and regional patterns

**FIGURE 2:**
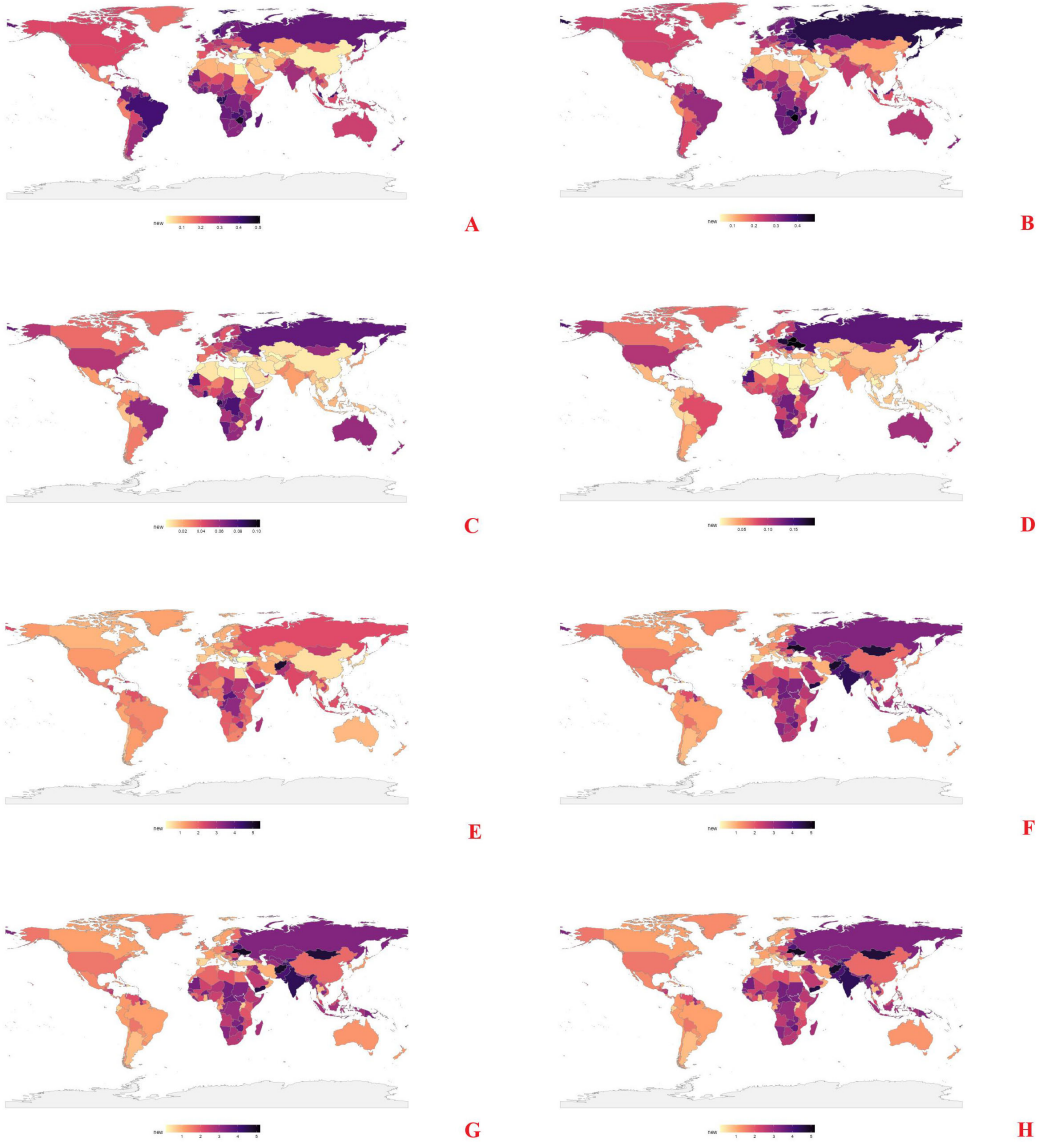
Spatial distribution of ASMR caused by inadequate F&V intake in 2021. **(A)** AA, **(C)** PAD, **(E)** IHD, **(G)** HHD, attributed to insufficient intake of vegetables; **(B)** AA, **(D)** PAD, **(F)** IHD, **(H)** HHD, attributed to insufficient intake of fruits.

Substantial cross-country variation has been observed (Figure 2). ASMR for AA and PAD were lowest in North Africa and highest in Russia, Brazil, and Oceania, as well as in parts of North America. For IHD, high burdens related to low vegetables intake have been seen in Russia and Central Africa, and high fruit-related IHD mortality was particularly evident in Russia. The highest HHD burdens attributable to low vegetables intake were found in Mongolia and Central Africa, while China and India recorded the greatest fruit-related HHD mortality.

Temporal trends from 1990 to 2021 (Supplementary Figure 2) showed wide regional disparities. AA mortality linked to low vegetables intake rose sharply in North Africa; fruit-related AA mortality increased in Russia, Central Africa, Brazil, and Oceania. PAD mortality increased in China and India (vegetable), and in North America and Russia (fruit). For IHD, ASMR declined in North America and Australia but rose in several parts of Asia, Africa, and Latin America. HHD mortality associated with low fruits intake increased consistently, with Mongolia, China, and India exhibiting the largest gains.

### 3.3 Age, sex, and SDI differences

**FIGURE 3:**
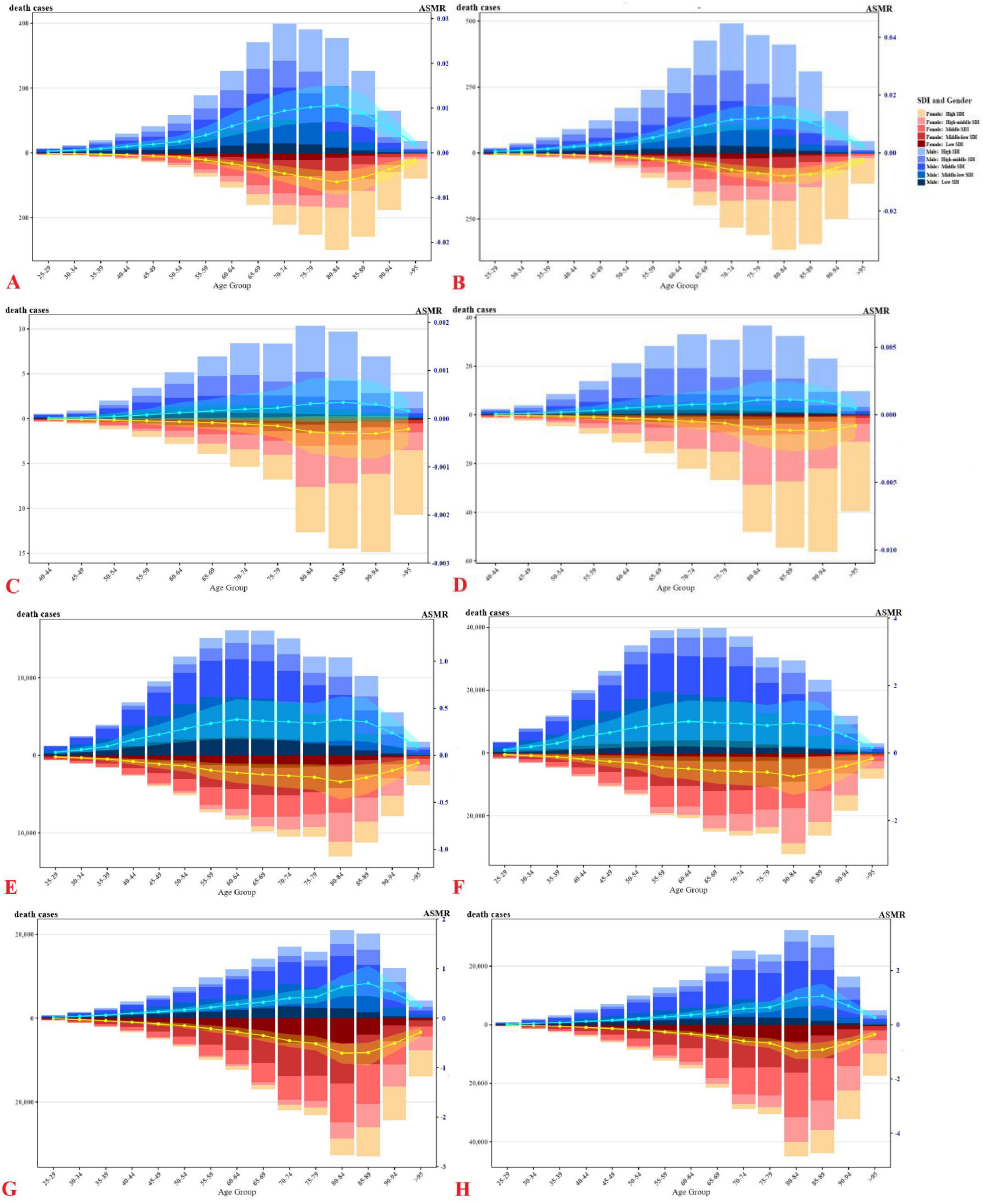
Number and proportion of global CVDs deaths caused by inadequate F&V intake in 2021, divided by age, gender, and SDI levels. **(A)** AA, **(C)** PAD, **(E)** IHD, **(G)** HHD, attributed to insufficient intake of vegetables; **(B)** AA, **(D)** PAD, **(F)** IHD, **(H)** HHD, attributed to insufficient intake of fruits; bar chart represents death cases, line chart represents ASMR.

Age-specific patterns (Figure 3) showed a consistent increase in mortality with age, peaking between 65 and 84 years for most CVDs. PAD mortality peaked between 75 and 94 years, particularly among women in high-SDI settings. IHD mortality rose from age 50 onward, while HHD showed sustained burden through age 85. Similar trends were observed for fruit-related outcomes.

Men exhibited higher mortality rates for AA, PAD, and IHD, whereas HHD mortality was greater in women. By SDI, AA and PAD deaths predominated in high and high-middle SDI countries, while IHD and HHD burdens were concentrated in middle and low-SDI settings. These gradients reflect underlying disparities in diet quality, healthcare access, and competing risks. Patterns in DALYs (Supplementary Figure 3) mirrored those of mortality, highlighting the combined burden of premature death and disability.

### 3.4 SDI gradients and temporal trends

**FIGURE 4:**
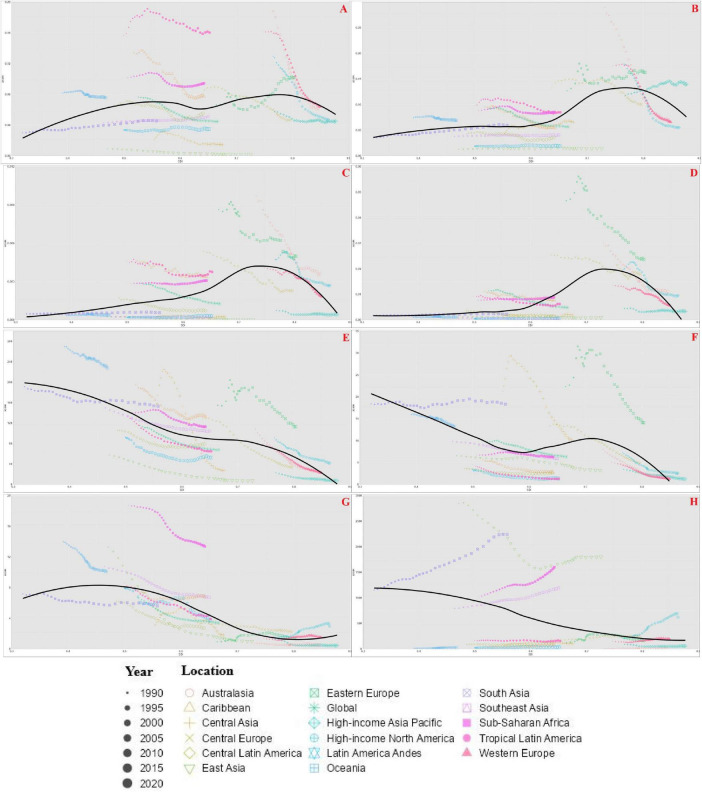
Age-standardized mortality rate (ASMR) caused by inadequate F&V intake in 17 GBD regions from 1990 to 2021. **(A)** AA, **(C)** PAD, **(E)** IHD, **(G)** HHD, attributed to insufficient intake of vegetables; **(B)** AA, **(D)** PAD, **(F)** IHD, **(H)** HHD, attributed to insufficient intake of fruits.

Regional trends in ASMR (Figure 4, Supplementary Figure 4) varied by CVD type and dietary risk. AA mortality linked to low vegetables intake declined in most regions but rose in Eastern Europe, South Asia, and Sub-Saharan Africa. Fruit-related AA mortality increased in high-income Asia Pacific, Eastern Europe, and Central and South Asia, while declining in Oceania, North America, and Western Europe.

Peripheral artery disease-related mortality increased in tropical Latin America, South Asia, and Sub-Saharan Africa (vegetable), but fruit-related PAD mortality declined in all regions except Sub-Saharan Africa. IHD ASMR declined globally for both dietary risks. In contrast, HHD mortality associated with low fruits intake increased across all regions, particularly in East Asia and high-income North America.

**FIGURE 5:**
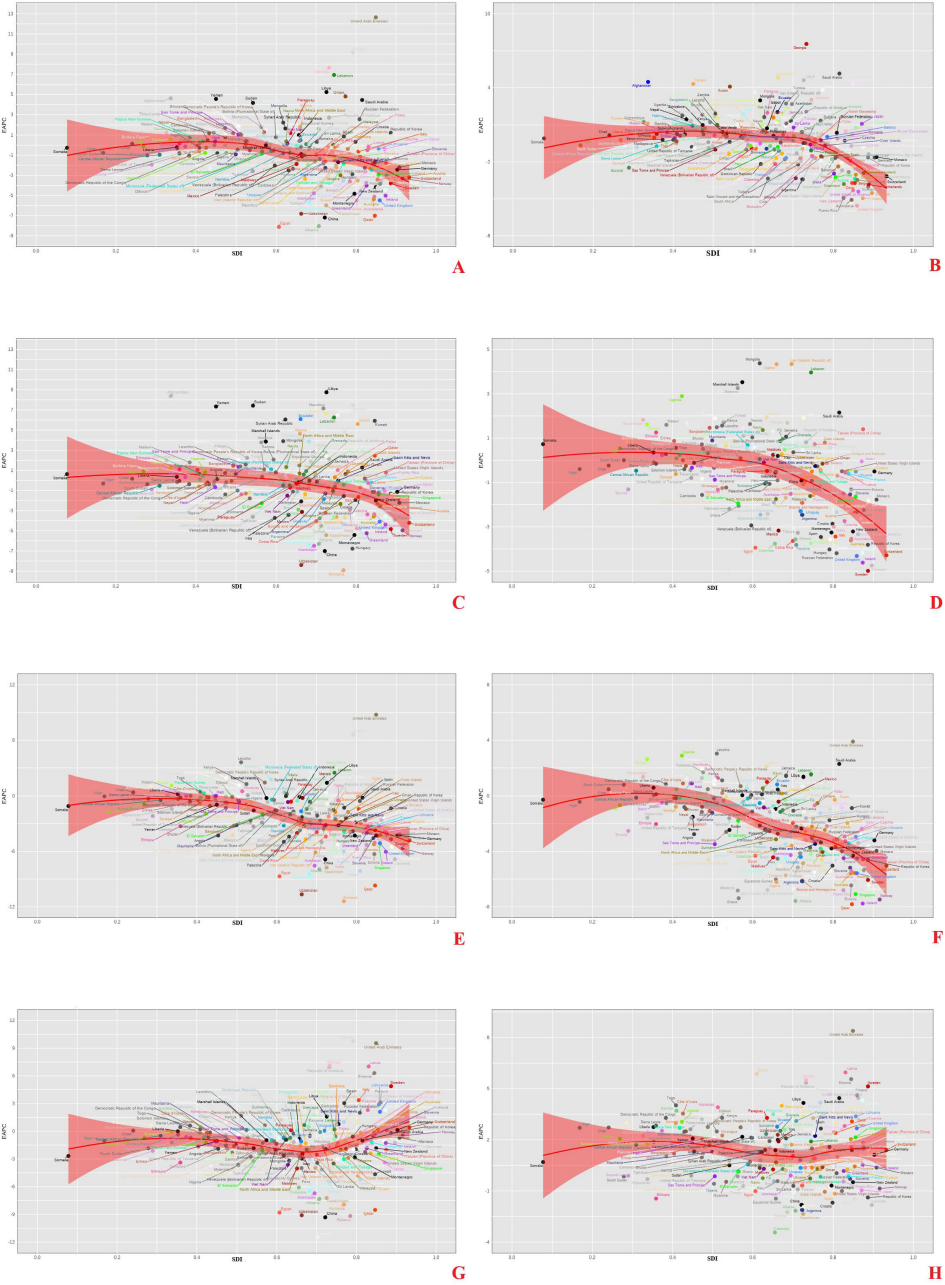
Relationship between the global burden of AA caused by inadequate F&V intake and SDI in 204 countries and regions, EAPC of ASMR. **(A)** AA, **(C)** PAD, **(E)** IHD, **(G)** HHD, attributed to insufficient intake of vegetables; **(B)** AA, **(D)** PAD, **(F)** IHD, **(H)** HHD, attributed to insufficient intake of fruits.

Age-standardized DALY rate trends paralleled ASMR. IHD burden declined steadily, while PAD and HHD exhibited divergent regional trajectories, particularly in low-and middle-SDI settings. Figure 5, Supplementary Figure 5 highlights national EAPC patterns: UAE, Georgia, and Lebanon recorded the highest increases in vegetable-related mortality, while Egypt, Albania, and China had the largest declines. For fruit-related CVDs, Georgia and Mongolia showed the steepest increase while China, Sweden, and the UK showed the sharpest reductions.

### 3.5 Decomposition analysis findings on mortality burden drivers

**FIGURE 6:**
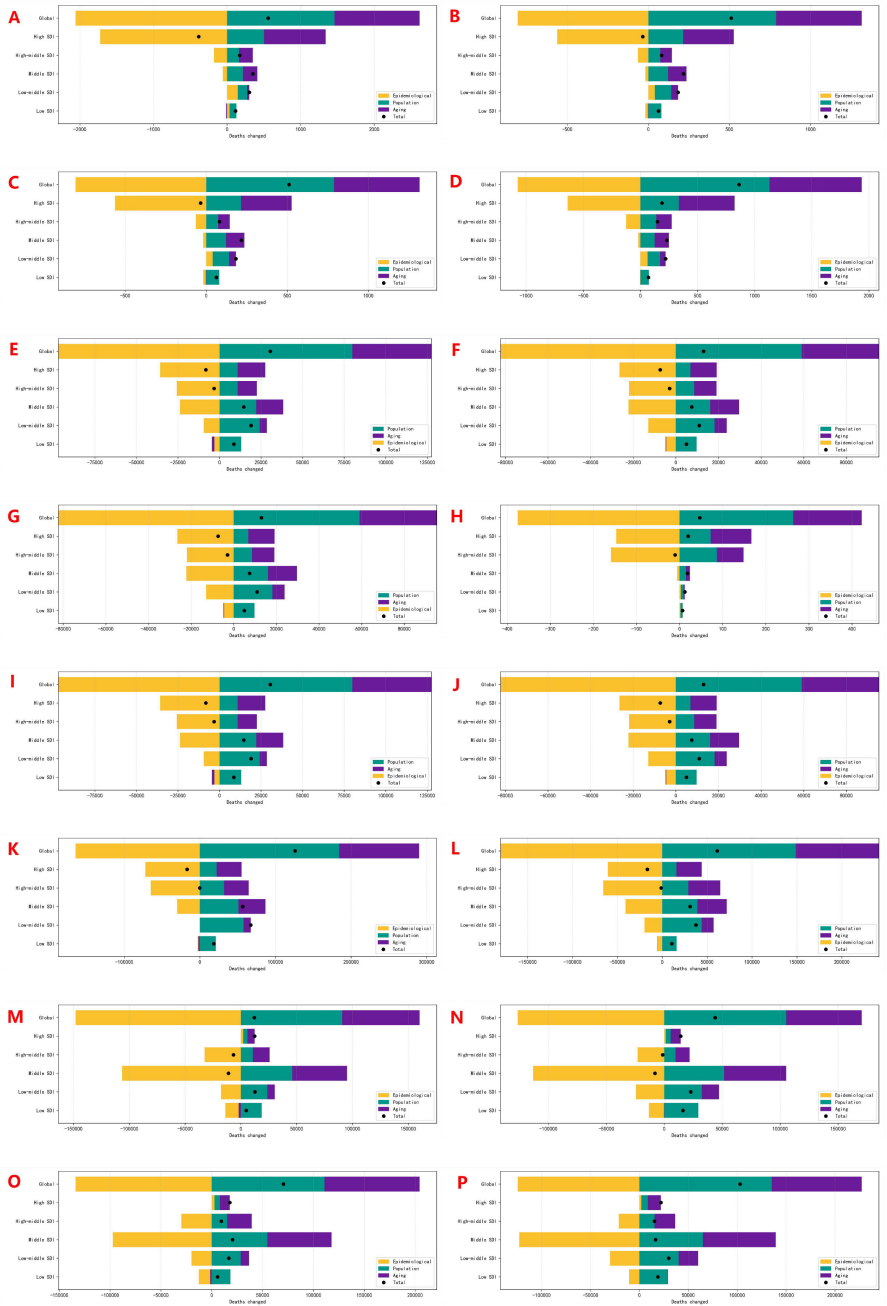
Decomposition analysis of cardiovascular disease mortality attributable to insufficient F&V intake: contributions of age structure, population growth, and epidemiological changes, 1990–2021. **(A,B)** Male/Female AA; **(E,F)** Male/Female PAD; **(I,J)** Male/Female IHD; **(M,N)** Male/Female HHD: attributed to insufficient vegetables intake. **(C,D)** Male/Female AA; **(G,H)** Male/Female PAD; **(K,L)** Male/Female IHD; **(O,P)** Male/Female HHD: attributed to insufficient fruits intake.

As shown in Figure 6, population aging was the dominant contributor to rising mortality in high-SDI countries, though overall deaths declined due to improved risk management and healthcare. In contrast, in low-SDI regions, both population growth and unfavorable epidemiological transitions contributed to increased burden.

For AA and PAD, global mortality increased, but net reductions were observed in high-SDI regions. IHD and HHD mortality attributable to dietary risks rose globally. While IHD burden declined in some high-SDI regions, HHD mortality linked to low fruits intake increased across all SDI categories, underscoring persistent gaps in dietary prevention.

### 3.6 Projections to 2044

Projections (Supplementary Figures 6, 7) suggest continued increases in absolute deaths due to aging, alongside a plateau in ASMR. These trends reflect the saturation of earlier public health gains and ongoing dietary risk exposures. The burden remains sex-specific, with higher mortality from AA, PAD, and IHD in men, and from HHD in women. Without further intervention, these trends may reverse recent progress in reducing diet-related cardiovascular mortality.

## 4 Discussion

Fruits and vegetables played a pivotal role in cardiovascular protection, primarily through mechanisms involving blood pressure regulation, vascular endothelial support, and anti-inflammatory and antioxidant effects (24–26). While the WHO’s recommendation of a minimum daily intake of 400 g was supported by robust epidemiological and mechanistic evidence, this study extended this understanding by offering the first comprehensive, multi-decade assessment of the global burden of four major CVDs (IHD, HHD, PAD, and AA) attributable to inadequate F&V consumption. The analysis of this study revealed significant temporal, geographic, demographic, and socioeconomic heterogeneity, highlighting areas where policy and prevention efforts remained insufficient.

The distribution of diet-related CVDs burden was strikingly uneven. High-SDI countries exhibited the highest ASMR for AA and PAD, potentially reflecting both aging populations and enhanced diagnostic capacity. In contrast, middle-SDI regions bore a disproportionate share of IHD and HHD burdens–likely due to dietary transitions, increased consumption of processed foods, urbanization, and constrained health system infrastructure. Notably, the lower attributable mortality observed in low-SDI regions may be misleading, potentially reflecting underdiagnosis, data gaps, and competing mortality risks rather than true protection from dietary risks (12).

Marked sex disparities have been observed: men showed consistently higher mortality from AA, PAD, and IHD, while women had higher HHD mortality, particularly related to low fruits intake. These differences likely reflect a combination of biological factors and structural inequities–such as unequal intra-household food distribution, disparities in hypertension awareness and control, and differences in healthcare utilization patterns between genders (27, 28).

Although global ASMR and ASDR for most diet-related CVDs have declined over the past three decades, these improvements are not uniformly distributed (29). Of particular concern is the consistent rise in HHD mortality attributable to low fruits intake across all SDI categories. This trend suggests that improvements in hypertension diagnosis and treatment have not been paralleled by sufficient progress in dietary prevention (30). Fruit–unlike vegetable–are often less accessible and more expensive, particularly in low-income and rural areas, where infrastructure limitations (e.g., refrigeration, transport) further constrain availability and affordability (31). These structural barriers persist despite broader awareness of the importance of diet for cardiovascular health (32).

The age-specific trends observed, particularly the inverted U-shaped mortality curve peaking between ages 60 and 84, reflected the cumulative nature of dietary risk (33, 34). IHD burden, spanning a wider age range, underscored the importance of life-course interventions. These findings supported early and sustained efforts to improve dietary quality, including integration of nutrition education into school curricula, workplace health promotion, and community-based interventions (35).

Geographically, countries such as Russia, India, Brazil, and Mongolia faced some of the highest burdens, whereas Mediterranean and East Asian regions performed comparatively better–likely due to enduring dietary traditions and stronger food system resilience (36). The EAPC analyses of this study revealed that rapid growth in countries like China and India had not been matched by proportional reductions in CVD burden, indicating that income gains without corresponding food policy reforms may exacerbate dietary inequalities (37).

The rise of AA mortality in Eastern Europe and Sub-Saharan Africa was an emerging public health concern. These regions underwent rapid epidemiological transitions, facing the dual challenges of persistent infectious disease burdens and rising non-communicable disease mortality. This dual burden underscored the need for integrated, multisectoral strategies that concurrently address nutrition, infectious disease, and chronic disease prevention (38).

The decomposition analysis of this study revealed that while population aging was a dominant driver of increased absolute deaths, improvements in clinical care and risk factor control had partially mitigated this impact in high-SDI countries. However, these gains were fragile. Without systemic action to address the root causes of dietary inadequacy–particularly fruit accessibility–progress might stagnate or even reverse.

To sustainably reduce diet-related CVDs burden, population-level strategies had to go beyond individual behavior change. Fiscal and regulatory measures had demonstrated potential in shaping healthier diets (39). These included F&V subsidies, taxes on ultra-processed foods, reform of agricultural subsidies to favor nutrient-dense crops, and zoning policies that improved healthy food access in urban settings. Public procurement policies, especially in schools and hospitals, could also normalize F&V consumption from an early age (40).

Global health frameworks had to embed nutrition equity as a core principle. Development assistance, trade policy, and food security efforts should have been aligned to address structural constraints in low- and middle-SDI countries. In particular, targeted investments in rural food infrastructure, cold chains, and agricultural diversification were essential to improve year-round fruit availability (41).

Mechanistically, future research should move beyond quantifying burden and explore causal pathways more deeply–such as how micronutrient deficiencies, dietary fiber intake, gut microbiota alterations, and metabolic inflammation mediate the relationship between F&V intake and CVDs. Understanding these pathways will enable the design of more precise interventions and allow tailoring of public health strategies to local contexts.

Finally, dietary surveillance must be significantly improved. Current systems, especially in resource-limited settings, lacked the granularity to capture meaningful patterns in consumption and risk exposure. Standardized, culturally adaptable dietary monitoring tools, integrated with national health surveys and electronic health records, will be critical to inform timely interventions and evaluate policy effectiveness.

In summary, the findings of this study point to both encouraging trends and emerging threats. While global age-standardized rates have improved, rising absolute mortality, growing disparities, and persistent dietary risk exposures threaten future progress. Bold, sustained, and systemic action is essential to ensure that dietary inadequacy does not undermine decades of achievement in cardiovascular disease prevention.

## 5 Limitations

This analysis was based on estimates from the GBD 2021 dataset, which, despite its breadth and methodological rigor, was subject to several limitations. First, variations in national health data systems and dietary surveillance might have introduced uncertainty, particularly in low-resource settings with limited reporting capacity. Second, risk attribution was derived from standardized exposure–response relationships, which might not have fully accounted for context-specific factors such as food quality, preparation methods, or co-existing risk exposures. Third, the modeling framework assumed constant exposure distributions and disease relationships over time, potentially underestimating the effects of future interventions, technological advances, or unforeseen disruptions. Finally, the projections of this study using the Nordpred model were based on historical trends and did not incorporate structural policy shifts that could alter future dietary patterns or cardiovascular outcomes.

## 6 Conclusion

This study presented a comprehensive assessment of the global burden of CVDs attributable to inadequate F&V intake, utilizing data from the GBD Study 2021 and projections extending to 2044. While age-standardized mortality and DALY rates had declined over recent decades, the absolute number of deaths from diet-attributable CVDs continued to rise–largely driven by population aging and ongoing exposure to dietary risk factors. This disease burden was unevenly distributed, with notable disparities across sociodemographic regions, age groups, and sexes. These findings underscored the urgent need for systemic interventions that go beyond modifying individual dietary behaviors. Structural barriers to F&V consumption–including challenges related to affordability, accessibility, and supply chain constraints–must be addressed through fiscal policies, food system reform, and targeted public health strategies. Without coordinated, multisectoral action, the current plateau in cardiovascular mortality might not be sustained, particularly in low- and middle-SDI regions, where inadequate F&V intake remains widespread.

## Data Availability

The original contributions presented in this study are included in this article/Supplementary material, further inquiries can be directed to the corresponding authors.

## Footnotes

1
https://vizhub.healthdata.org/gbd-results/
