# Supplementary material for: Global burden of four cardiovascular diseases attributable to low fruits and vegetables intake, 1990–2021, with a forecast to 2044
Source: Front Nutr. 2025 Oct 2;12:1655651. doi: 10.3389/fnut.2025.1655651 (PMC12527854; doi:10.3389/fnut.2025.1655651)
Supplement: Supplementary file 1 [file Data_Sheet_1.docx]

**Table S1 The ASMR and ASDR of cardiovascular diseases related to inadequate F&V intake globally, by SDI categories and GBD regions, 1990**

| Location | Insufficient fruits intake and CVDs 1990 | | Insufficient vegetables intake and CVDs 1990 | |
| --- | --- | --- | --- | --- |
|  | AA -ASMR(95%UI) | AA-ASDR(95%UI) | AA-ASMR(95%UI) | AA-ASDR(95%UI) |
| Global | 0.105(0.072,0.141) | 2.071(1.425,2.775) | 0.088(0.058,0.125) | 1.683(1.113,2.392) |
| High SDI | 0.205(0.141,0.276) | 4.030(2.795,5.398) | 0.165(0.108,0.237) | 3.127(2.054,4.495) |
| High-middle SDI | 0.075(0.052,0.101) | 1.753(1.212,2.364) | 0.050(0.031,0.073) | 1.080(0.677,1.594) |
| Middle SDI | 0.038(0.026,0.053) | 0.846(0.565,1.183) | 0.044(0.030,0.063) | 0.946(0.633,1.337) |
| Middle-low SDI | 0.041(0.025,0.064) | 0.892(0.545,1.423) | 0.041(0.025,0.064) | 0.886(0.541,1.392) |
| Low SDI | 0.058(0.032,0.101) | 1.239(0.677,2.186) | 0.068(0.036,0.124) | 1.453(0.779,2.678) |
|  | PAD-ASMR(95%UI) | PAD-ASDR(95%UI) | PAD-ASMR(95%UI) | PAD-ASDR(95%UI) |
| Global | 0.014(0.002,0.031) | 0.311(0.036,0.686) | 0.004(-0.001,0.009) | 0.077(-0.015,0.199) |
| High SDI | 0.019(0.002,0.042) | 0.423(0.051,0.927) | 0.005(-0.001,0.013) | 0.106(-0.022,0.271) |
| High-middle SDI | 0.023(0.003,0.051) | 0.452(0.052,0.997) | 0.005(-0.001,0.013) | 0.091(-0.018,0.234) |
| Middle SDI | 0.003(0.000,0.007) | 0.129(0.014,0.308) | 0.001(-0.000,0.003) | 0.041(-0.009,0.113) |
| Middle-low SDI | 0.003(0.000,0.007) | 0.117(0.013,0.282) | 0.001(-0.000,0.003) | 0.036(-0.007,0.099) |
| Low SDI | 0.005(0.001,0.015) | 0.151(0.015,0.382) | 0.002(-0.000,0.006) | 0.054(-0.008,0.156) |
|  | IHD-ASMR(95%UI) | IHD-ASDR(95%UI) | IHD-ASMR(95%UI) | IHD-ASDR(95%UI) |
| Global | 10.915(2.309,18.691) | 276.309(61.059,462.480) | 5.055(2.320,7.912) | 120.740(58.421,184.643) |
| High SDI | 7.898(1.625,13.810) | 178.359(37.923,304.859) | 4.277(1.885,6.863) | 91.127(42.033,143.557) |
| High-middle SDI | 12.029(2.455,20.926) | 279.344(59.129,475.110) | 4.396(1.919,7.112) | 95.430(43.522,152.011) |
| Middle SDI | 9.707(2.048,16.554) | 253.248(55.619,422.732) | 4.595(2.105,7.202) | 112.314(53.892,171.916) |
| Middle-low SDI | 14.919(3.319,25.166) | 420.266(97.206,693.978) | 6.717(3.159,10.318) | 178.293(87.237,267.542) |
| Low SDI | 12.123(2.656,20.833) | 324.355(73.908,547.272) | 7.921(3.718,12.202) | 201.718(97.427,306.047) |
|  | HHD-ASMR(95%UI) | HHD-ASDR(95%UI) | HHD-ASMR(95%UI) | HHD-ASDR(95%UI) |
| Global | 8.578(6.271,10.578) | 178.984(129.772,219.580) | 7.635(5.593,9.791) | 158.828(115.138,204.042) |
| High SDI | 2.561(2.044,3.051) | 52.504(42.968,61.665) | 2.068(1.554,2.654) | 40.876(30.994,52.203) |
| High-middle SDI | 6.415(4.598,8.410) | 123.930(89.624,160.651) | 4.898(3.338,6.822) | 93.616(64.591,129.289) |
| Middle SDI | 15.722(10.265,20.522) | 299.281(194.861,387.622) | 14.378(9.640,19.239) | 272.166(183.261,362.598) |
| Middle-low SDI | 12.421(8.707,16.280) | 255.037(176.424,332.823) | 11.269(7.770,14.912) | 228.417(155.467,302.477) |
| Low SDI | 18.706(12.193,24.731) | 393.125(247.961,525.109) | 20.204(13.145,26.950) | 422.300(268.173,570.020) |

AAA=Aortic Aneurysm;PAD=Peripheral Artery Disease;IHD=Ischaemic Heart Disease;HHD=Hypertensive Heart Disease;ASMR=Age-standardized Mortality Rate (per 100 000 population);ASDR, age-standardized DALY rate (per 100 000 population);UI=Uncertainty Interval;F&V=fruits and vegetables;SDI=Socio-demographic Index;GBD=Global Burden of Disease;CVDs=Cardiovascular Diseases. All rates are expressed per 100 000 population.

**
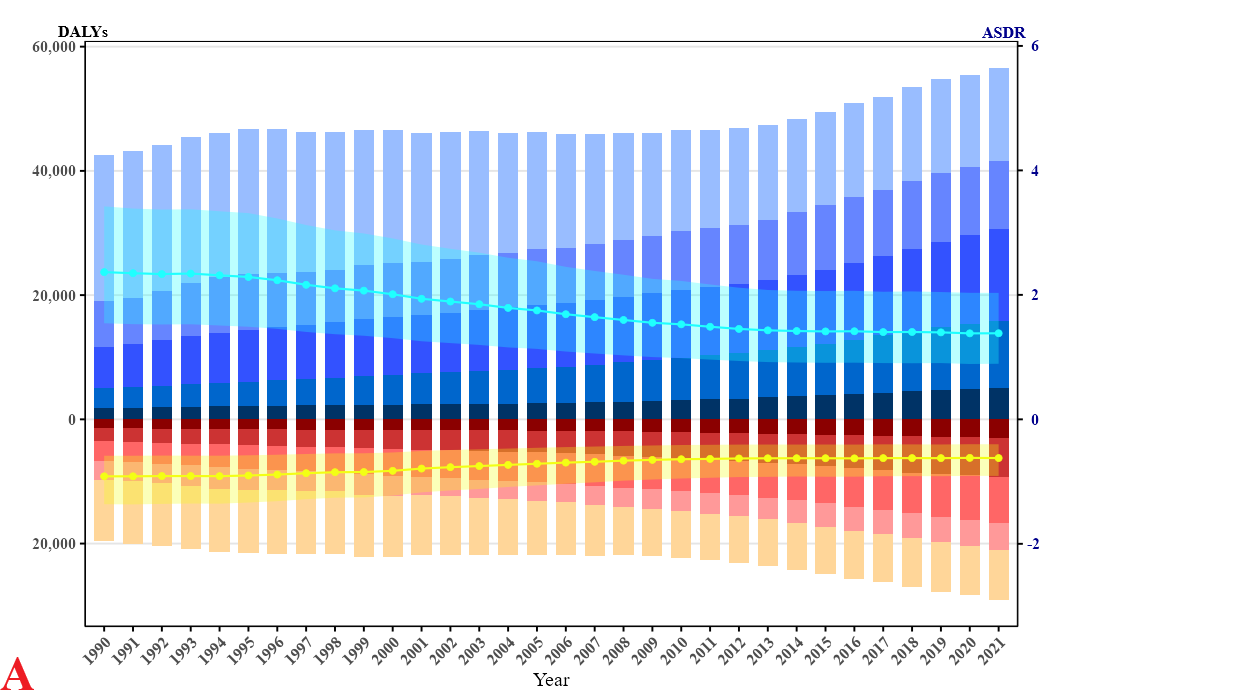

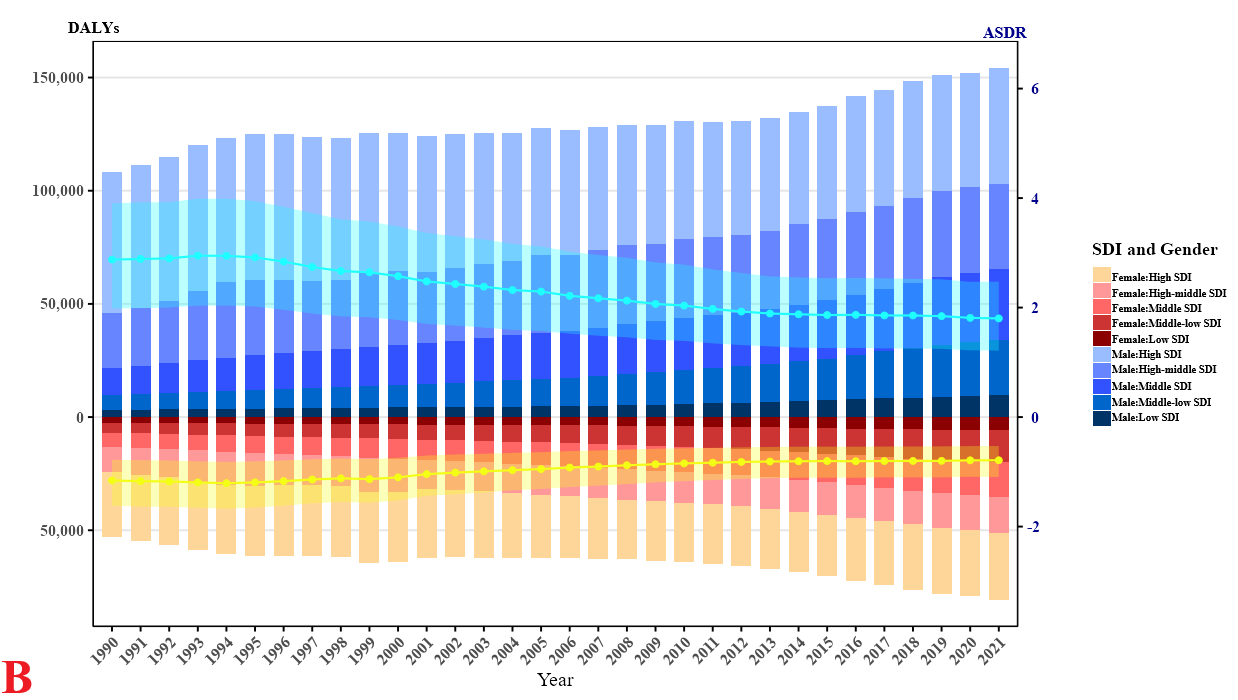
**

**
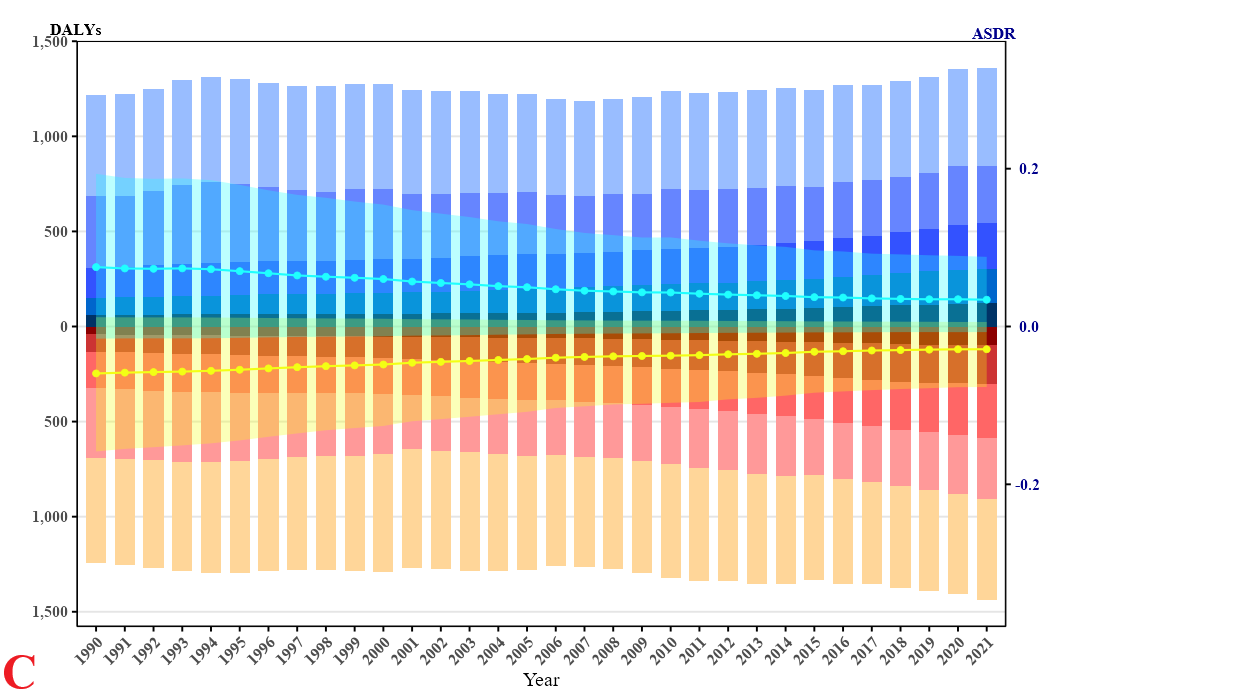

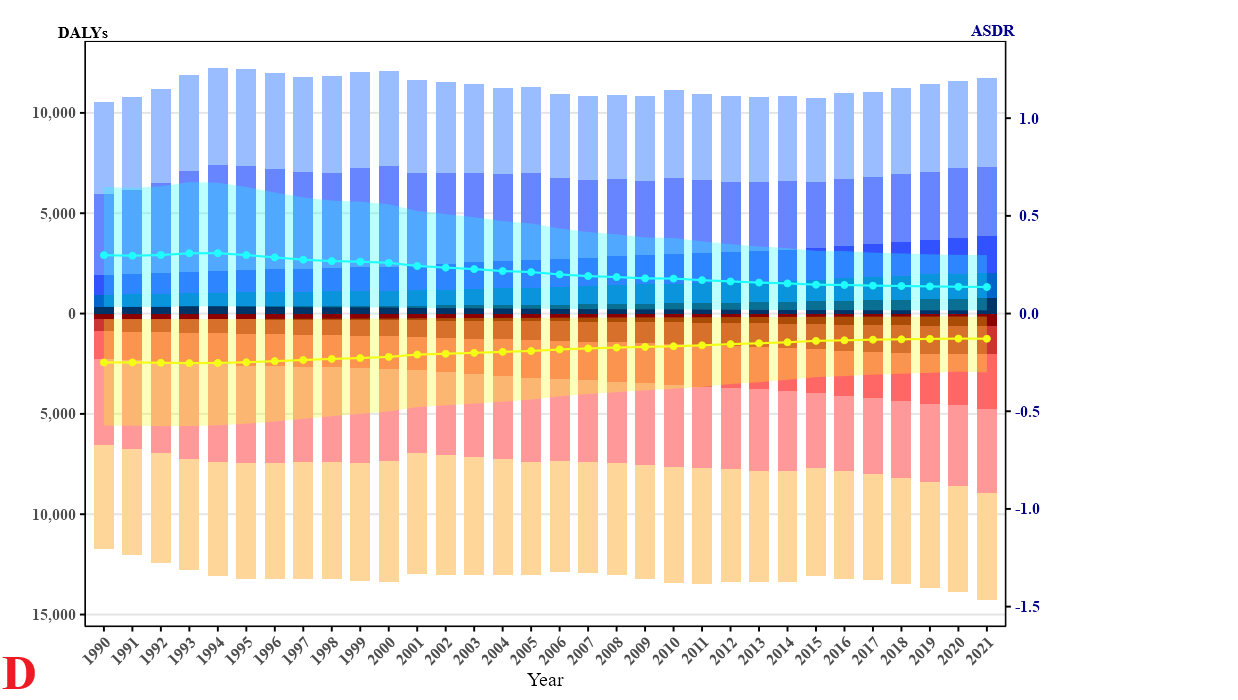
**

**
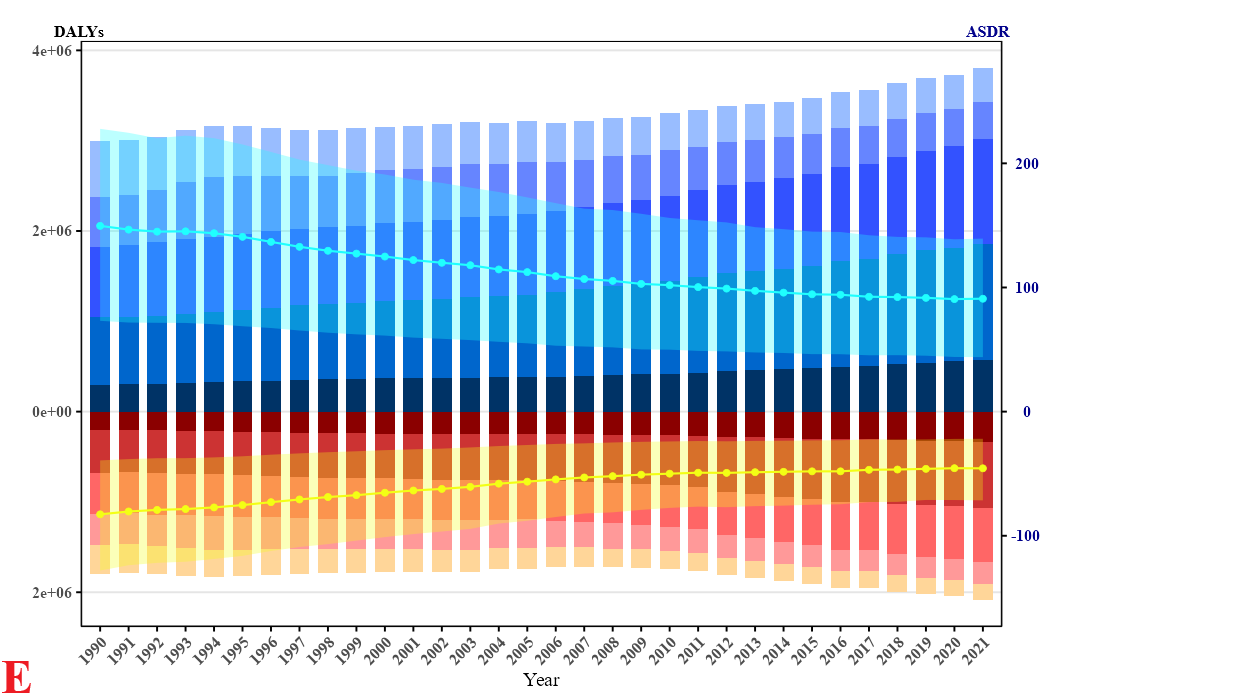

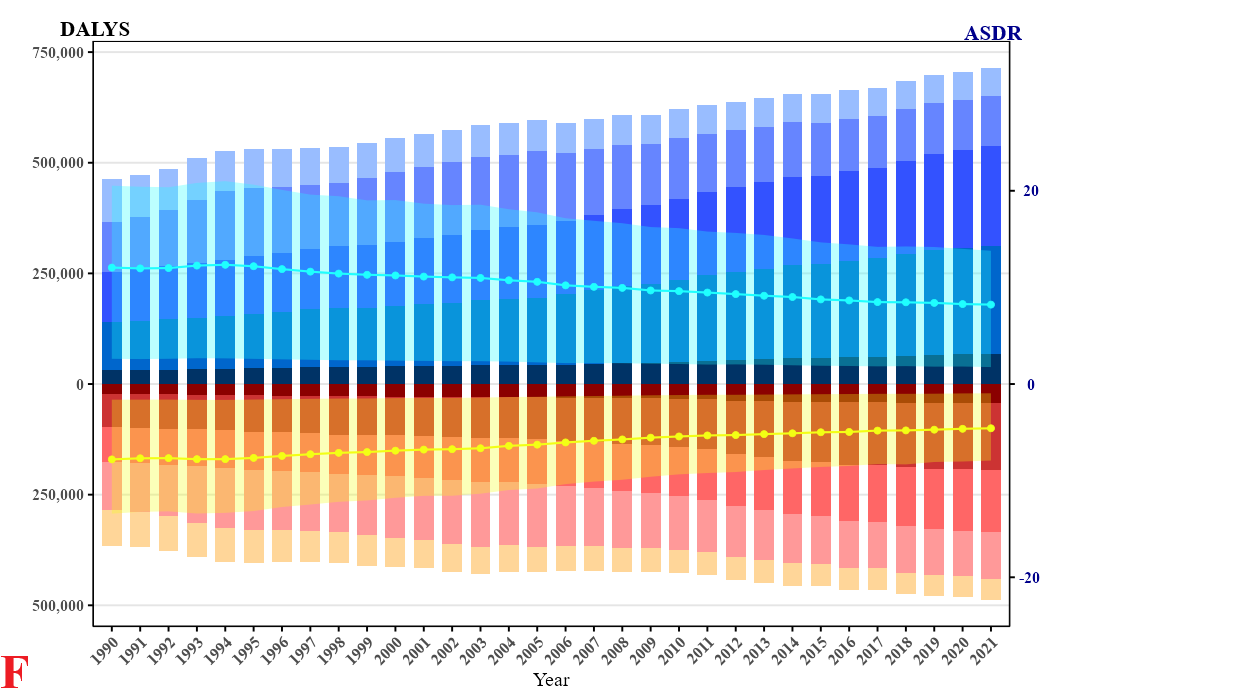
**

**
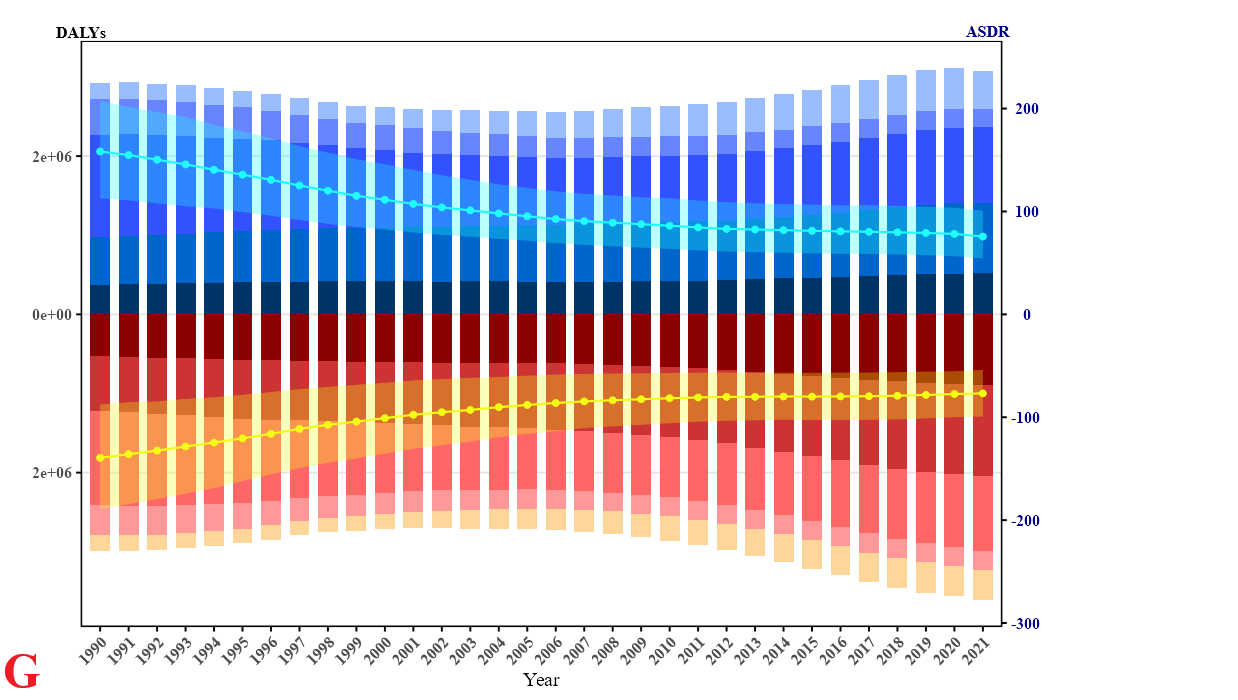

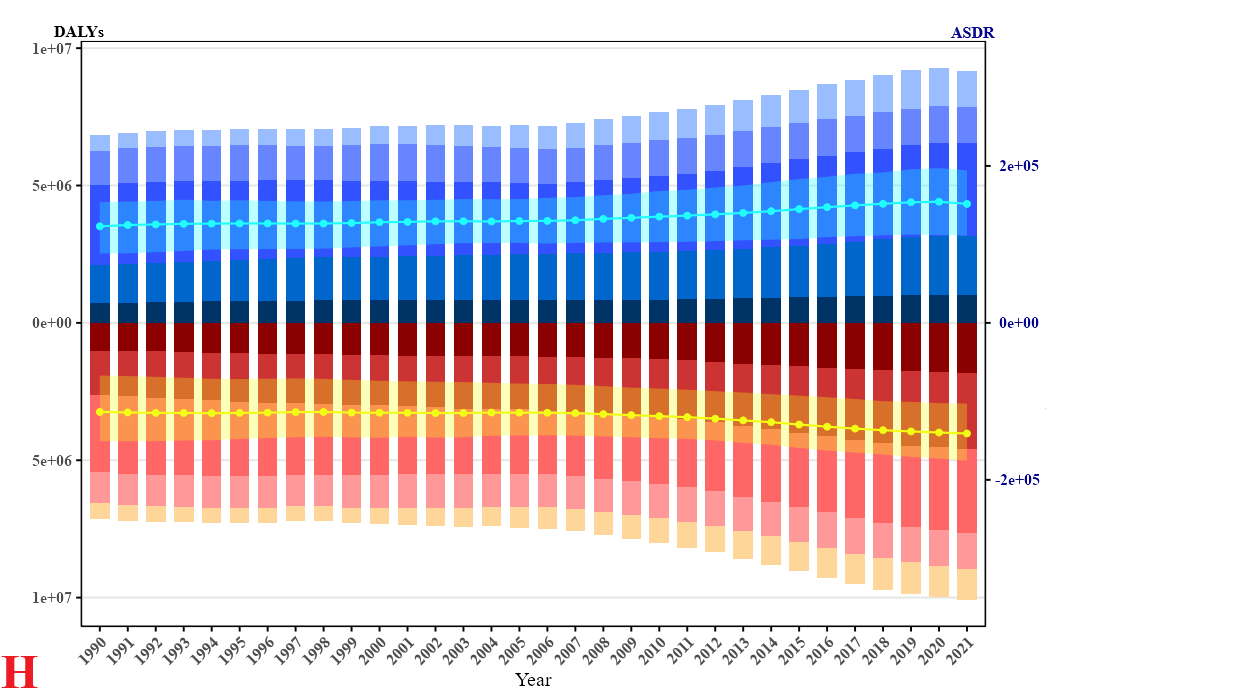
**

**Figure S1.DALYs and ASDR of CVDs Due to Inadequate F&V Intake (1990-2021), Divided by Gender, SDI Levels, and Region**

(A. AA, C. PAD, E. IHD, G. HHD, attributed to insufficient intake of vegetables; B. AA, D. PAD, F. IHD, H. HHD, attributed to insufficient intake of fruits;Bar chart represents DALYs, line chart represents ASDR,All rates are expressed per 100 000 population).

**
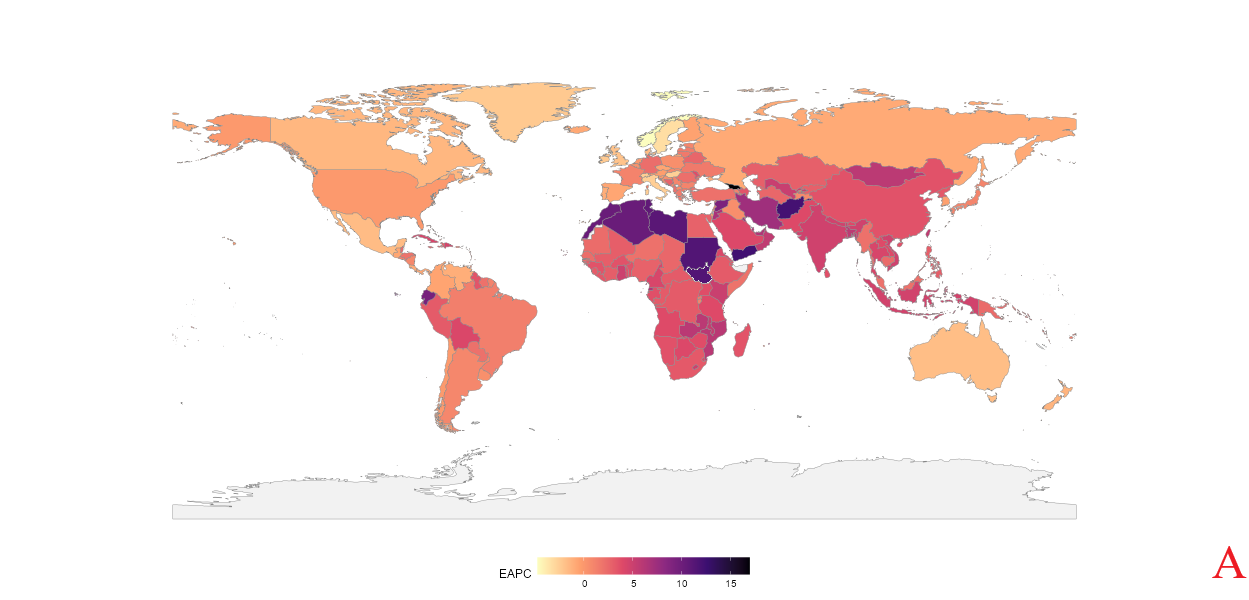

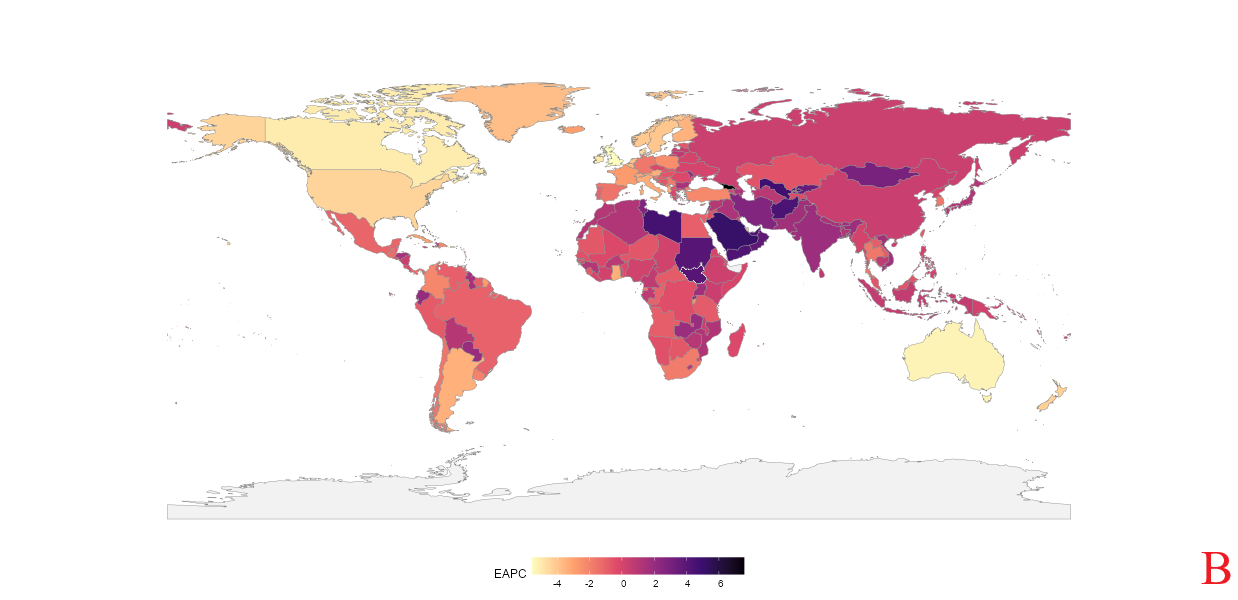
**

**
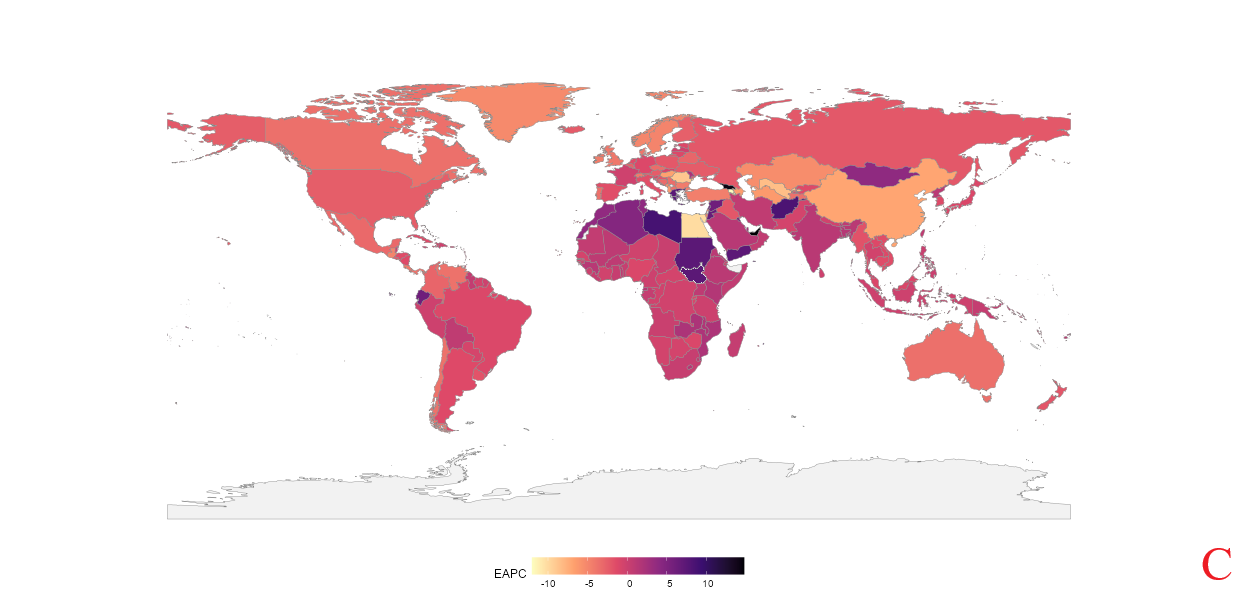

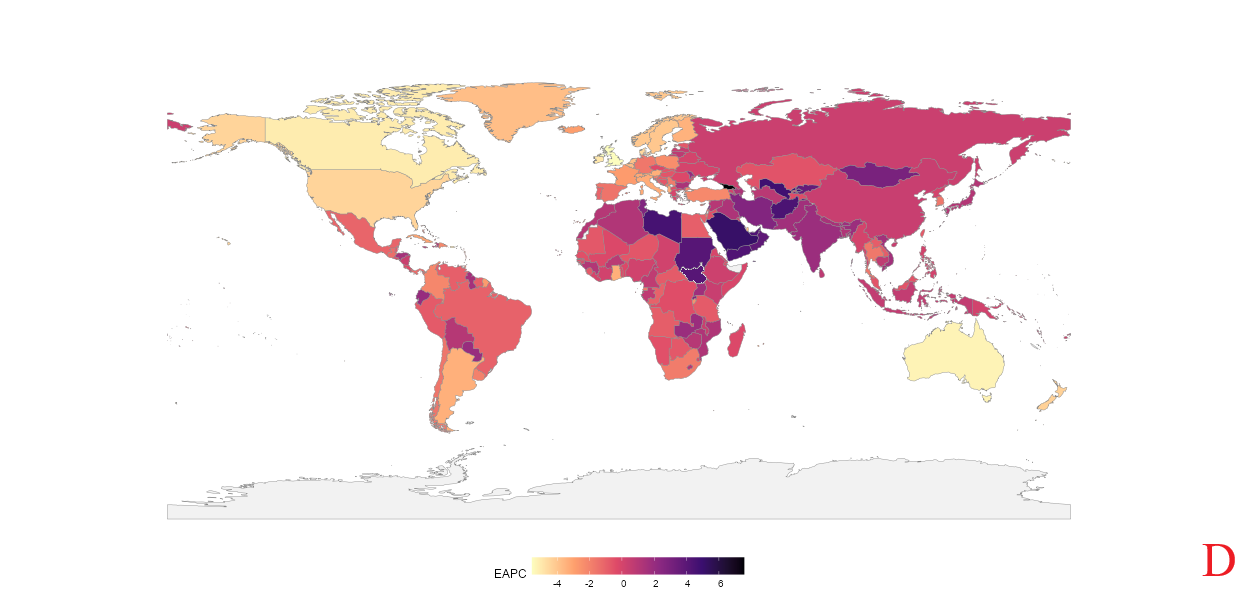
**

**
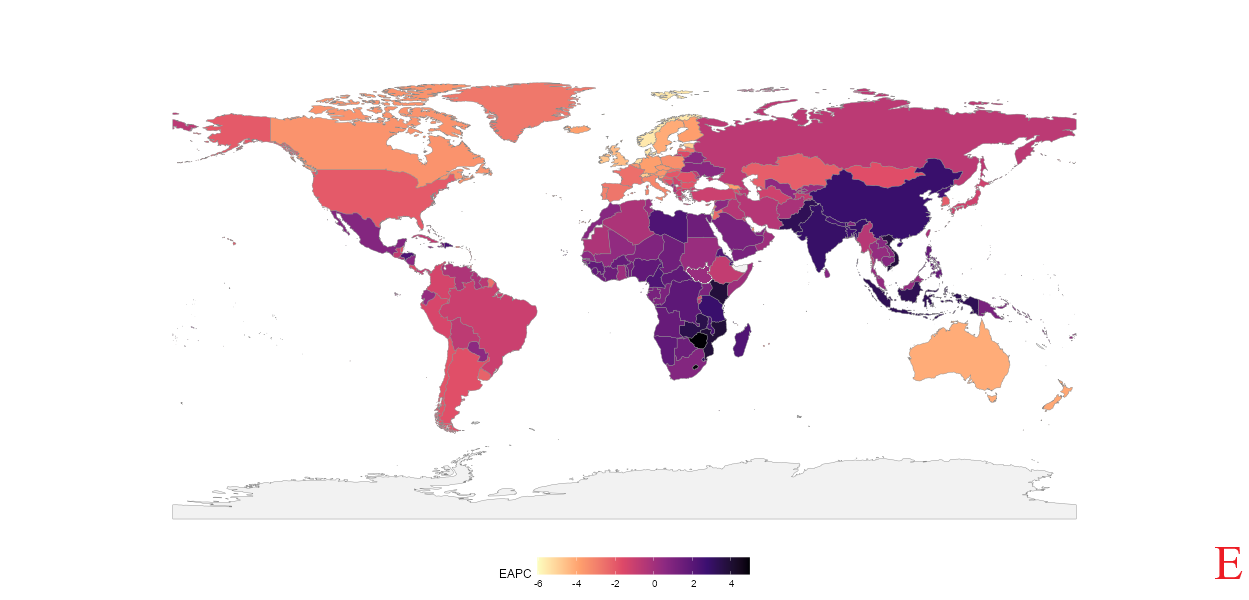

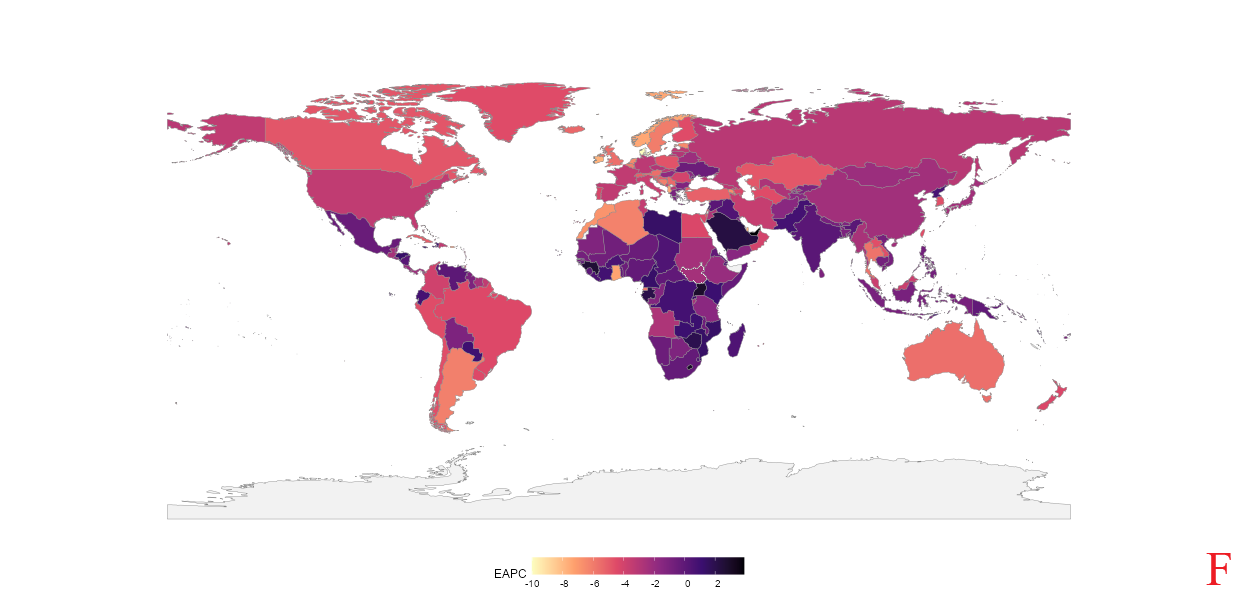
**

**
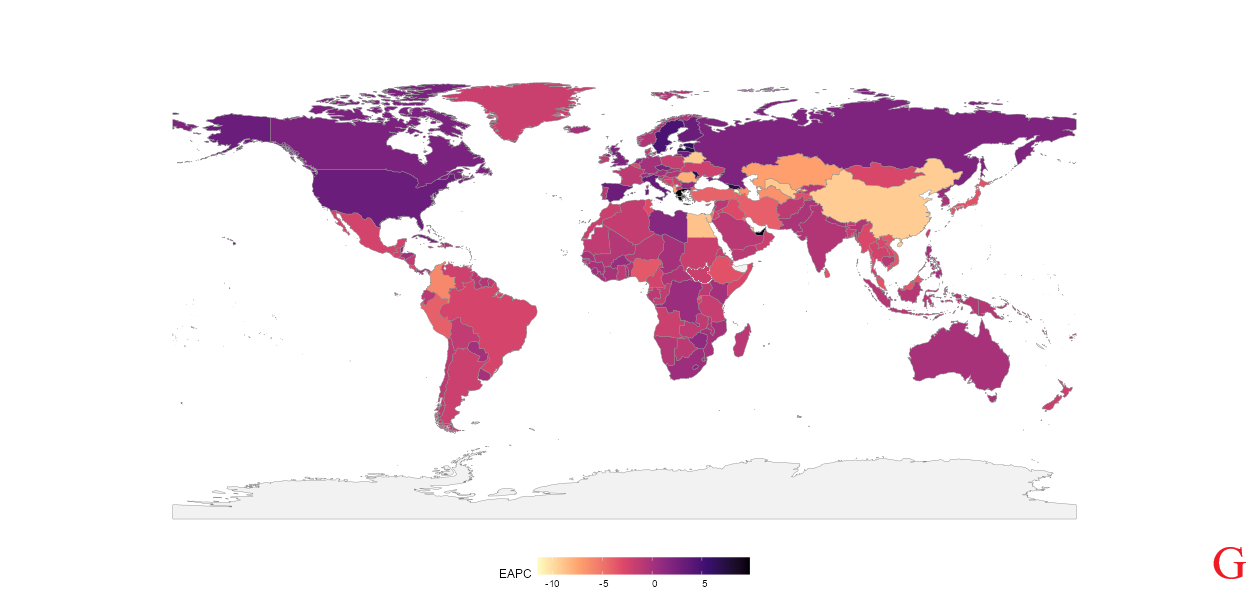

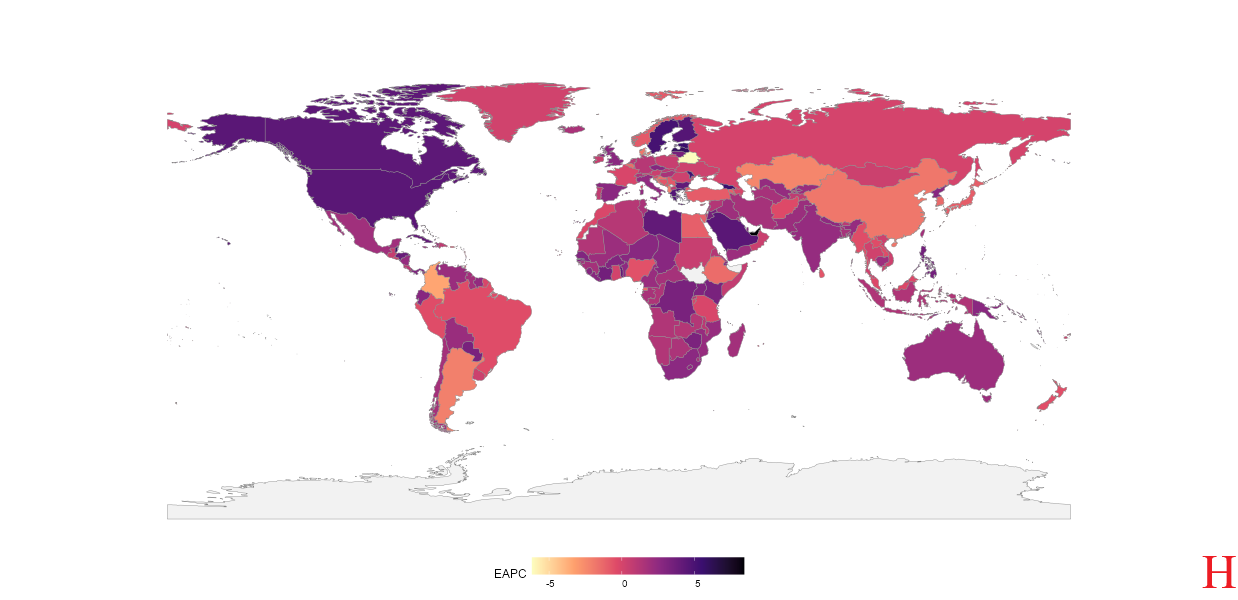
**

**Figure S2. Spatial distribution of EAPC of ASMR caused by inadequate F&V intake in 2021**

(A. AA, C. PAD, E. IHD, G. HHD, attributed to insufficient intake of vegetables; B. AA, D. PAD, F. IHD, H. HHD, attributed to insufficient intake of fruits.All rates are expressed per 100 000 population)


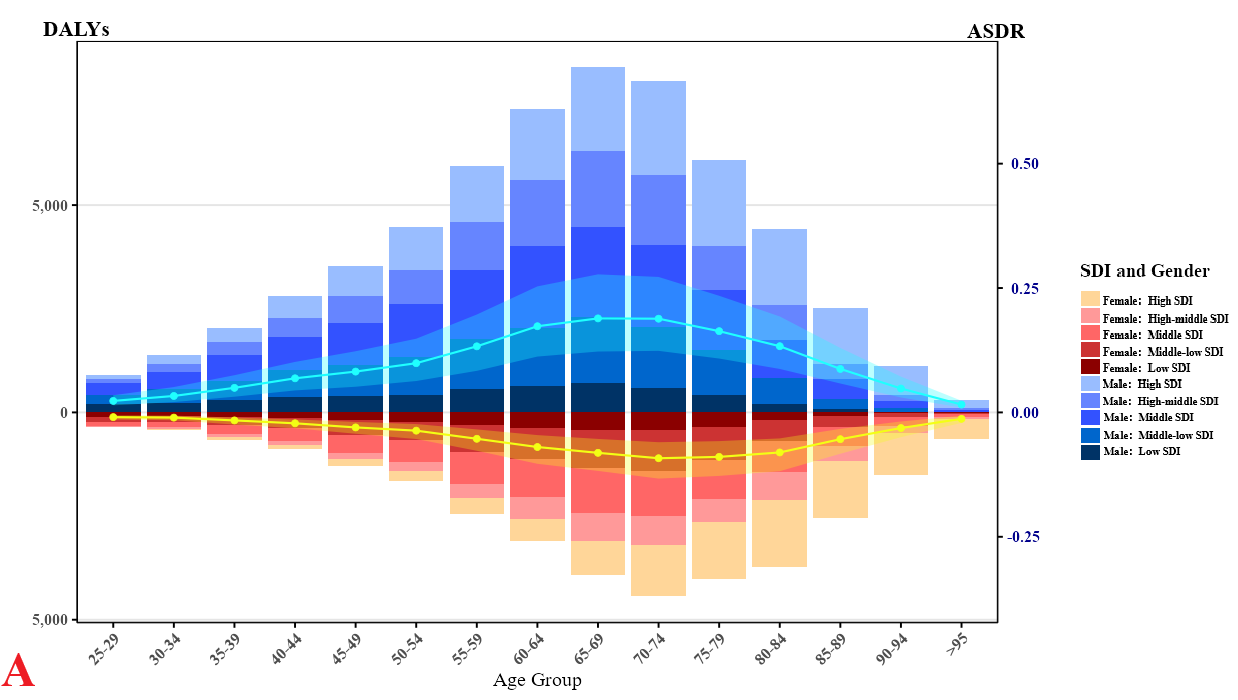

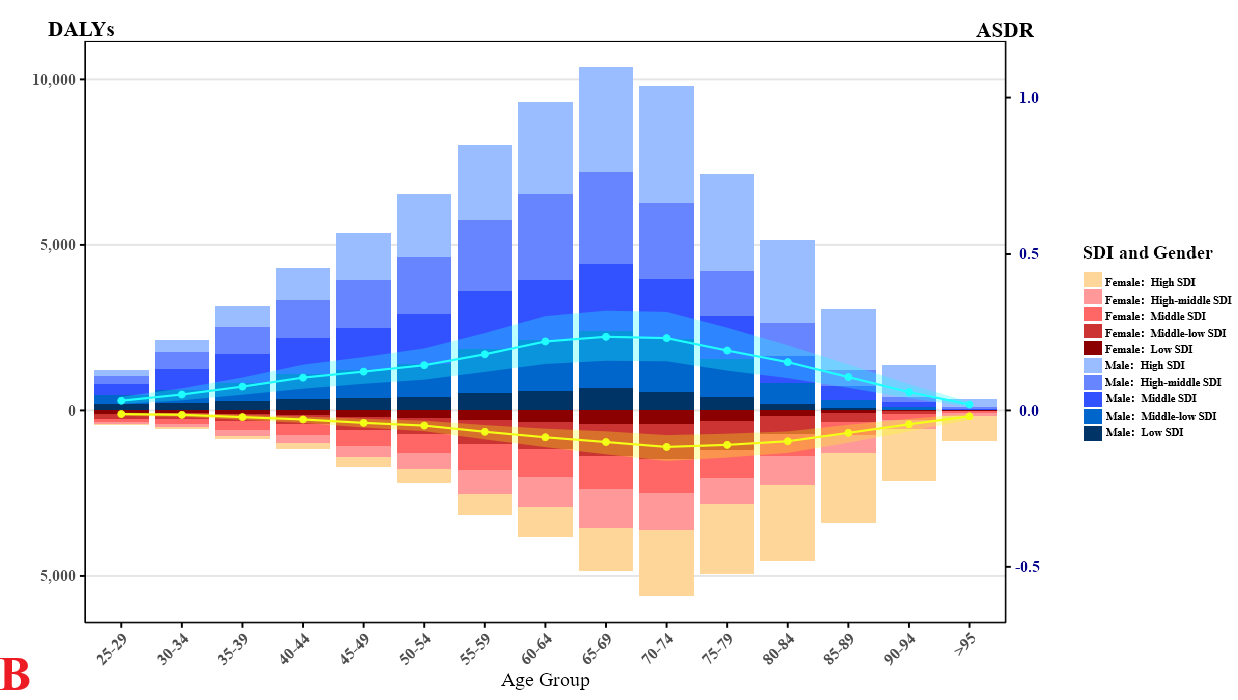


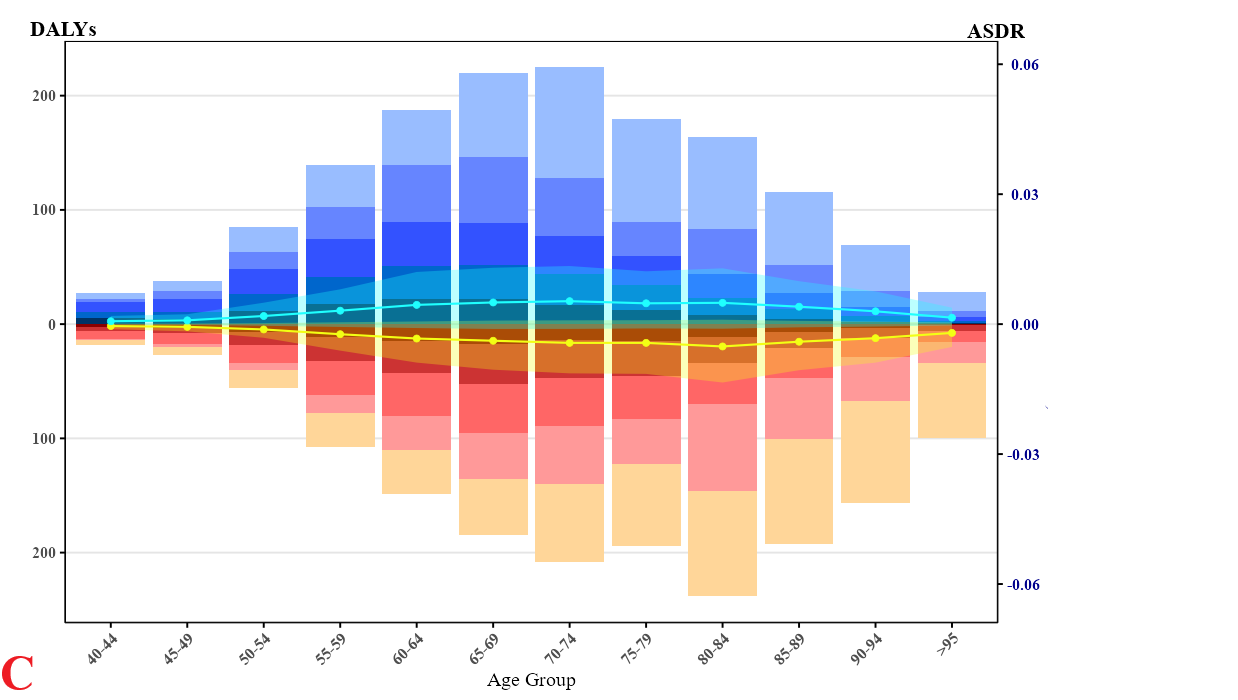

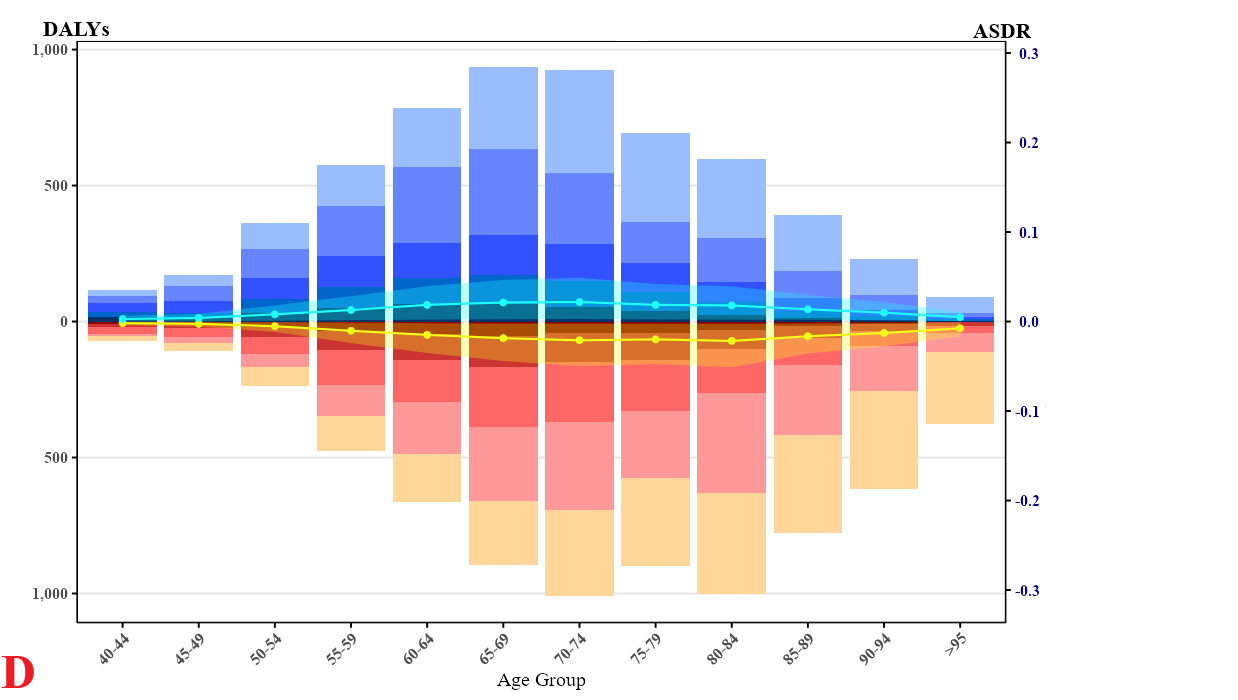


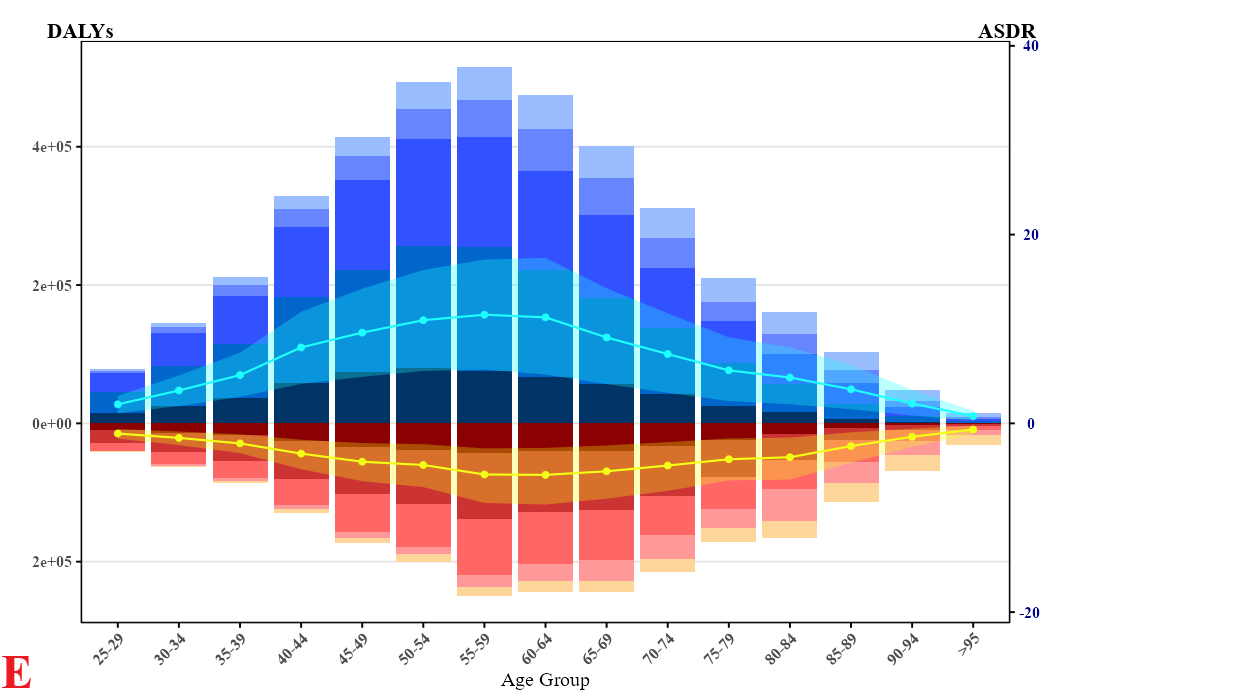

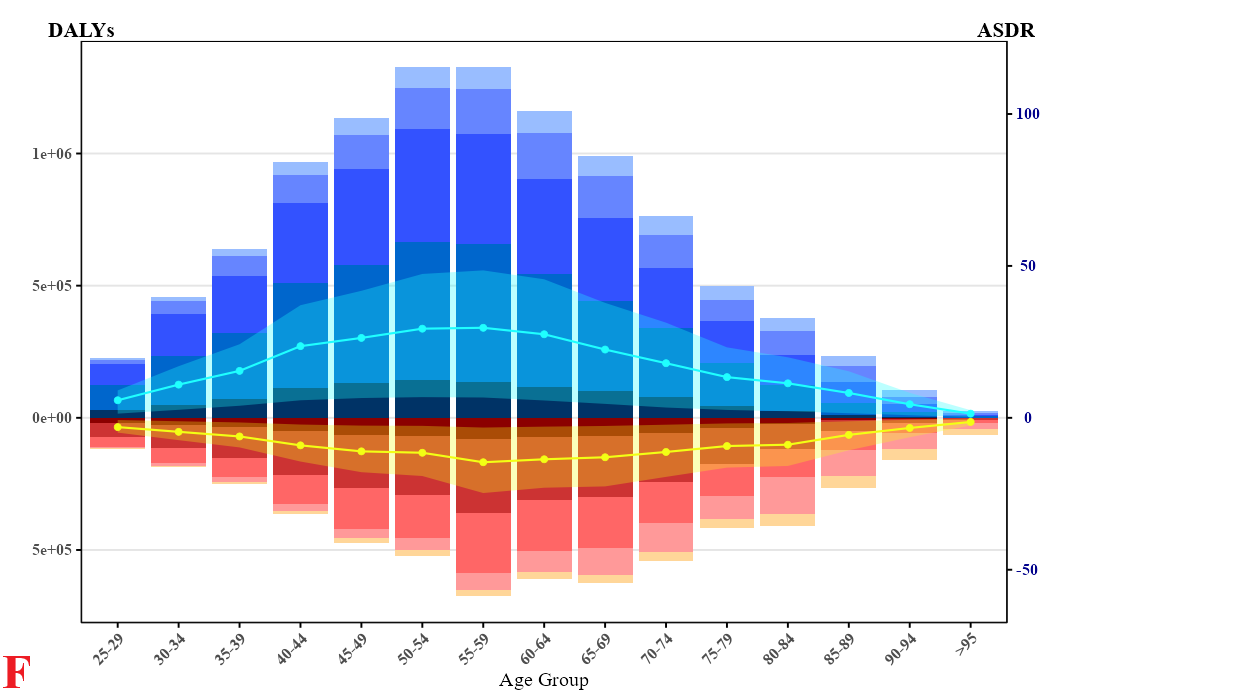


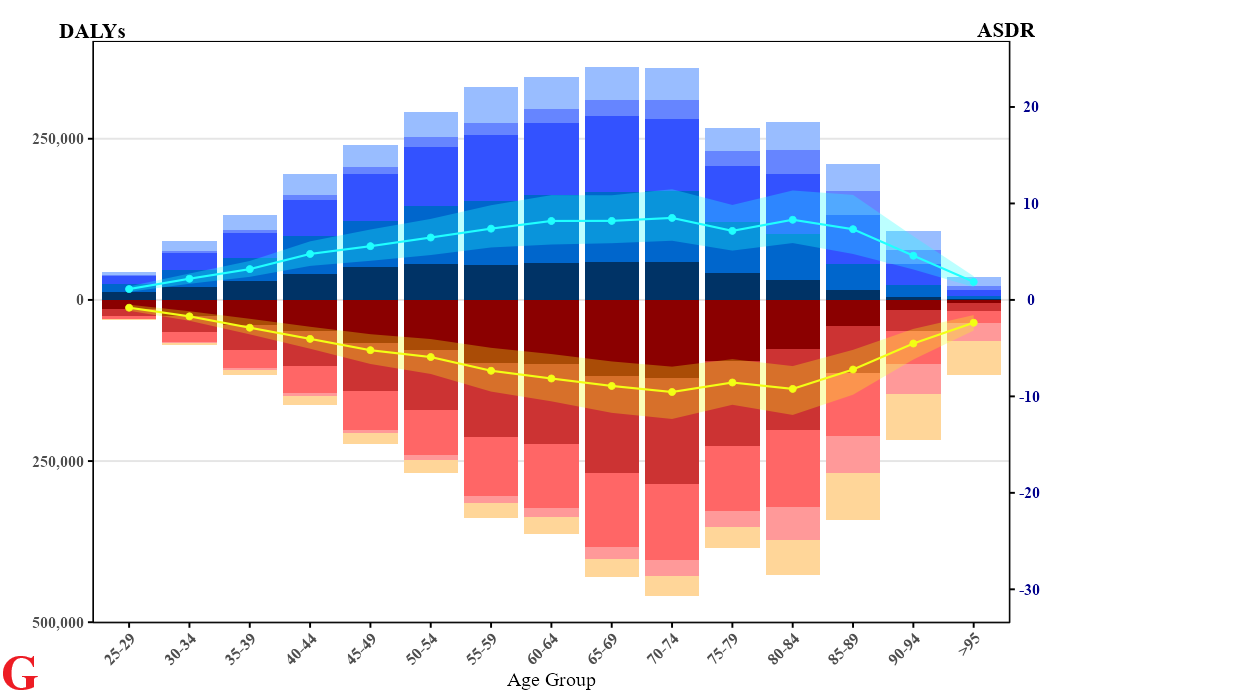

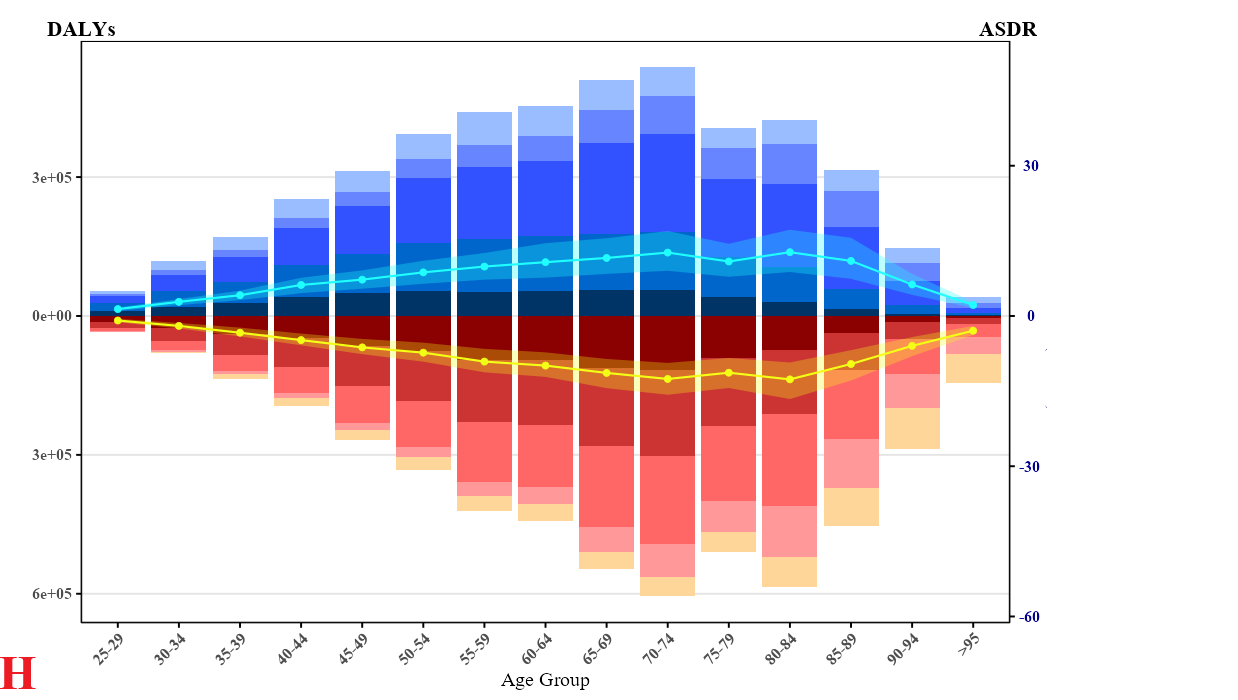


**Figure S3.Number and proportion of global CVDs DALYs caused by inadequate F&V intake in 2021, divided by age, gender, and SDI levels**

(A. AA, C. PAD, E. IHD, G. HHD, attributed to insufficient intake of vegetables; B. AA, D. PAD, F. IHD, H. HHD, attributed to insufficient intake of fruits;Bar chart represents DALYs, line chart represents ASDR,All rates are expressed per 100 000 population.)

**
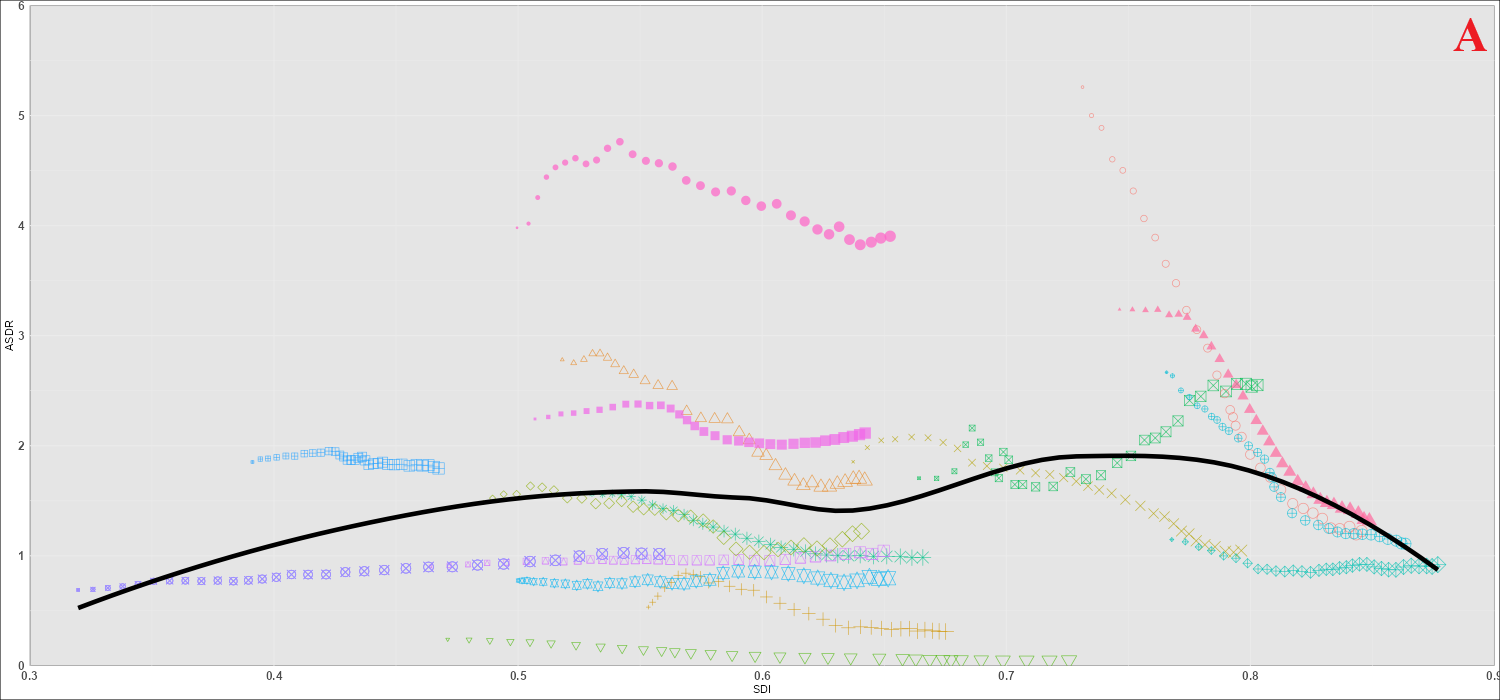

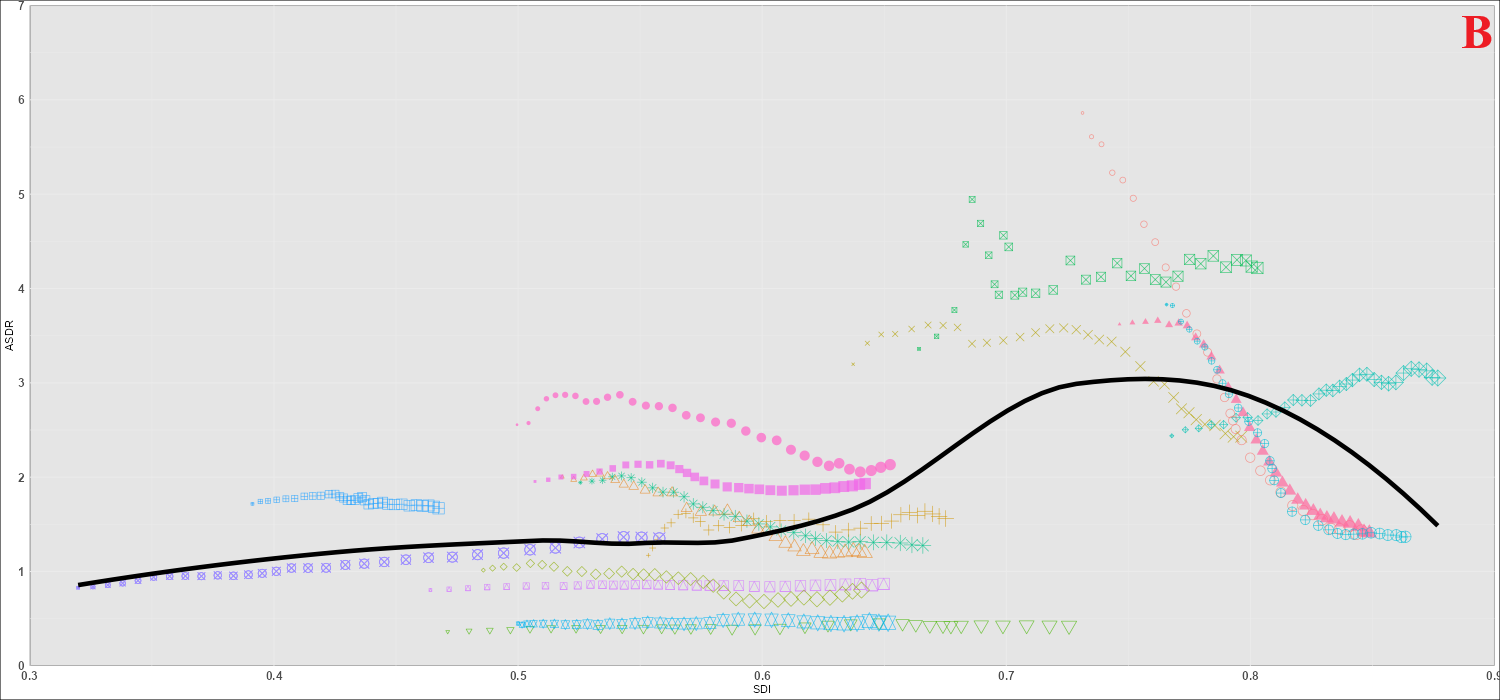
**

**
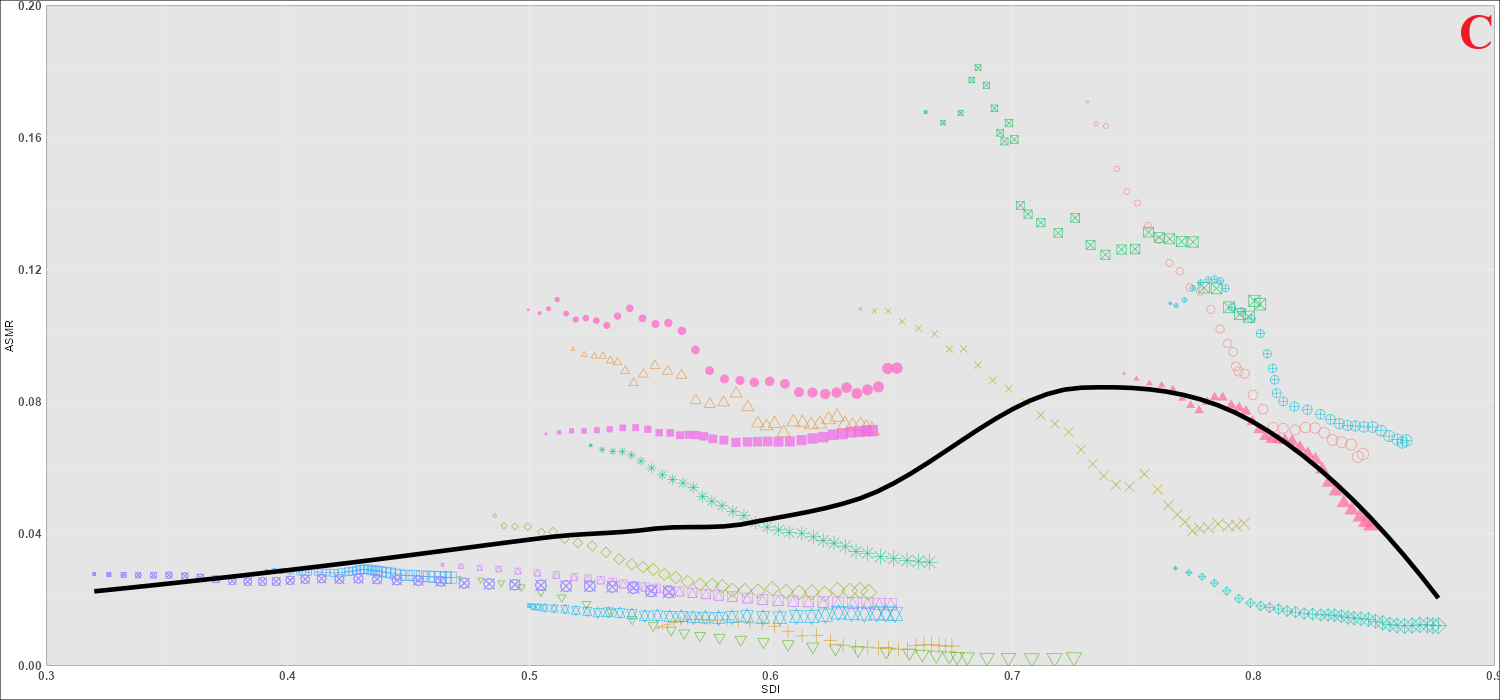

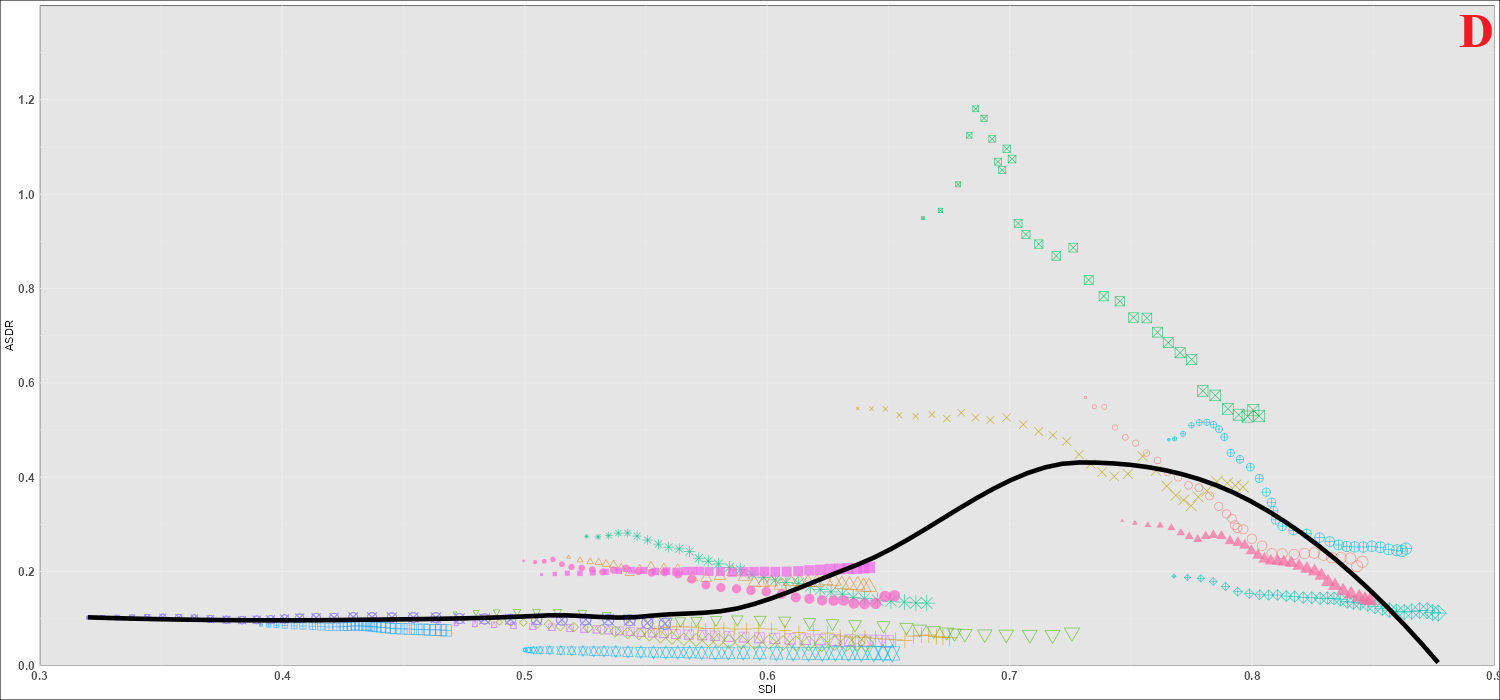
**


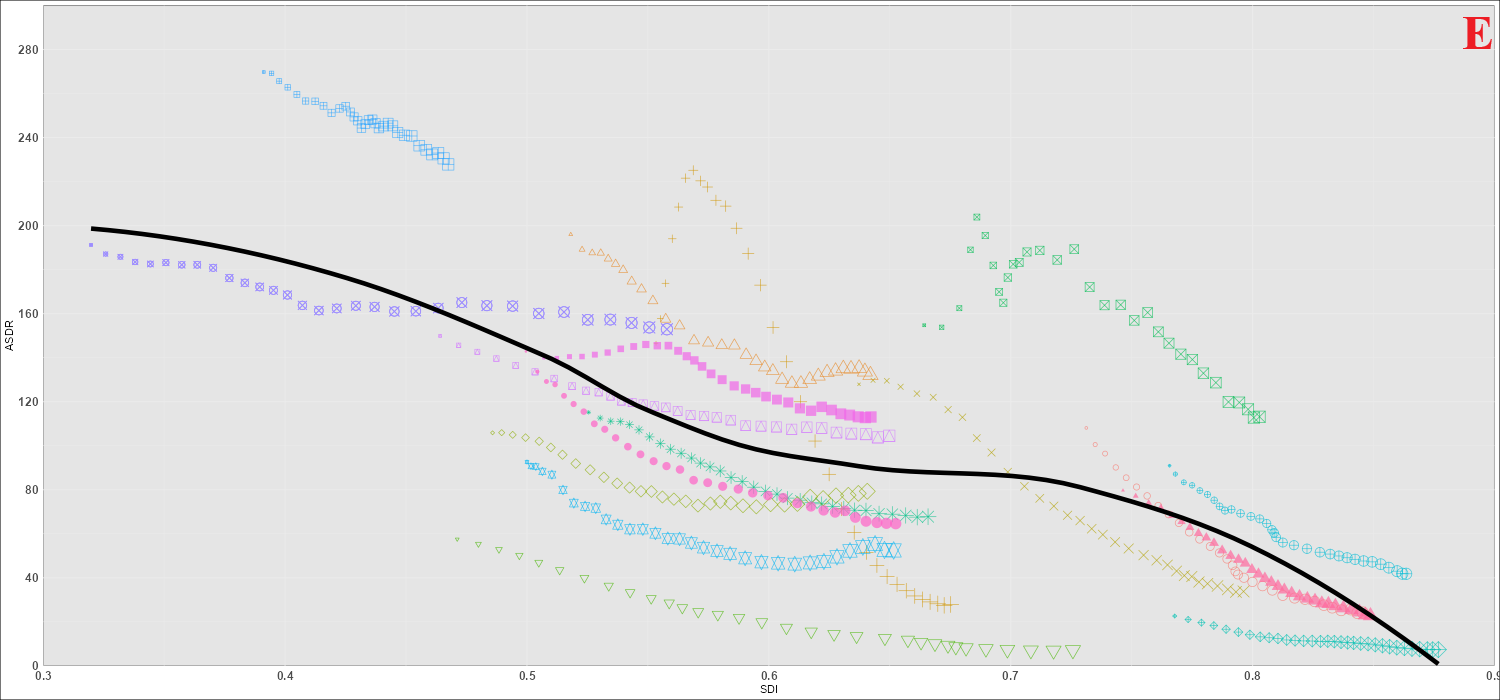

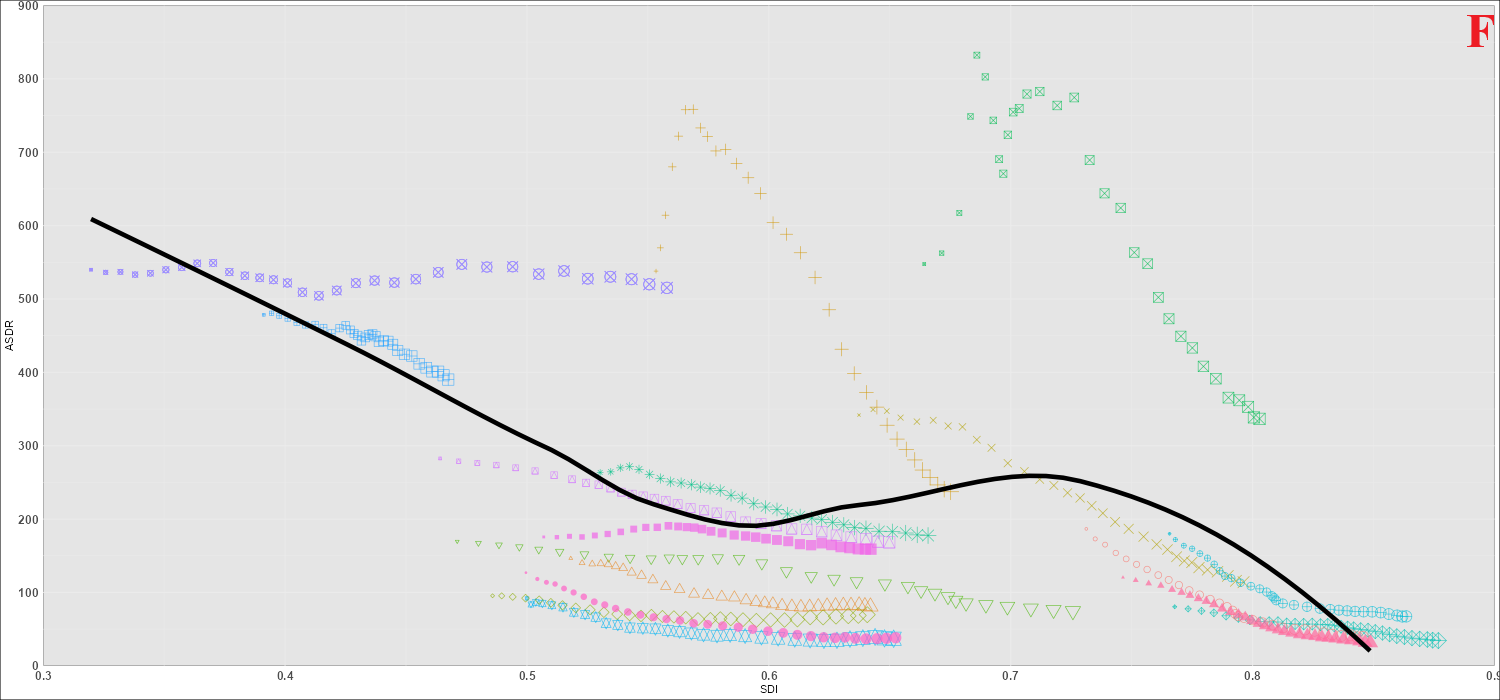


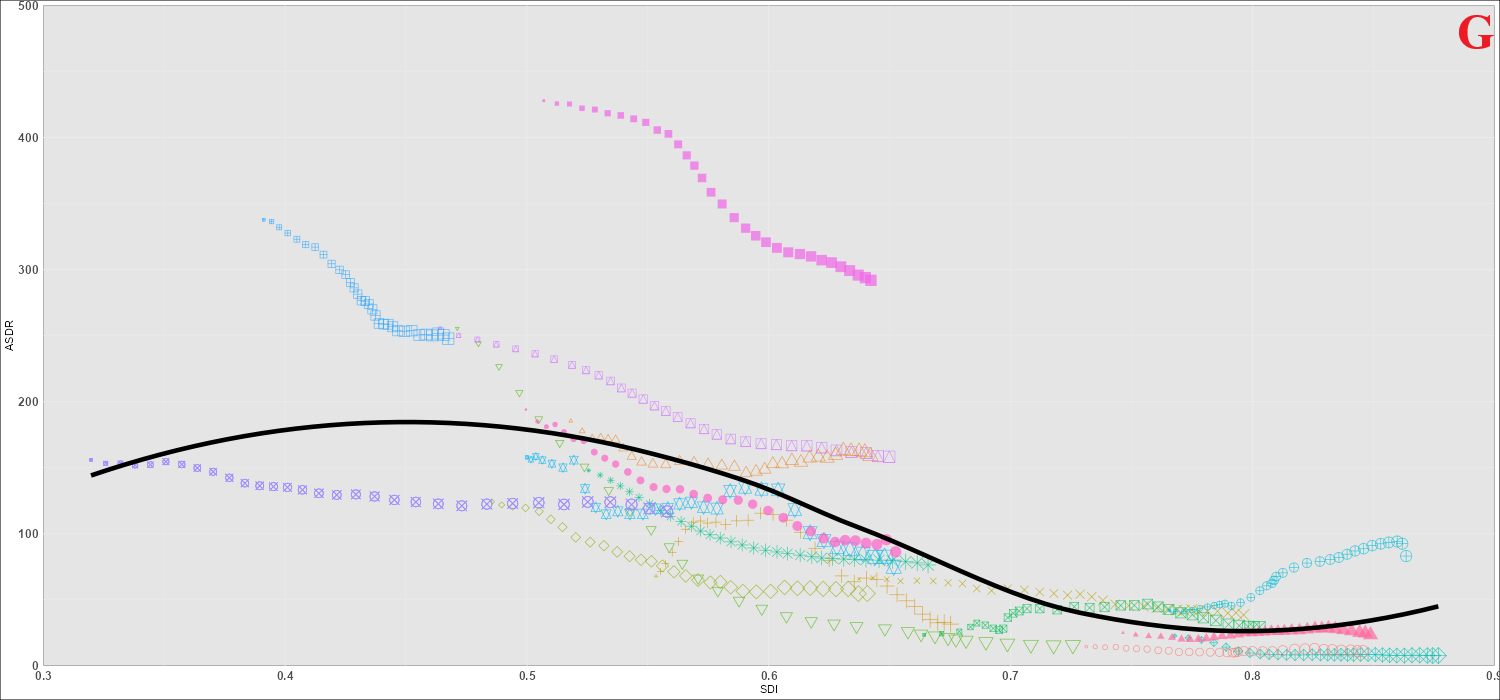

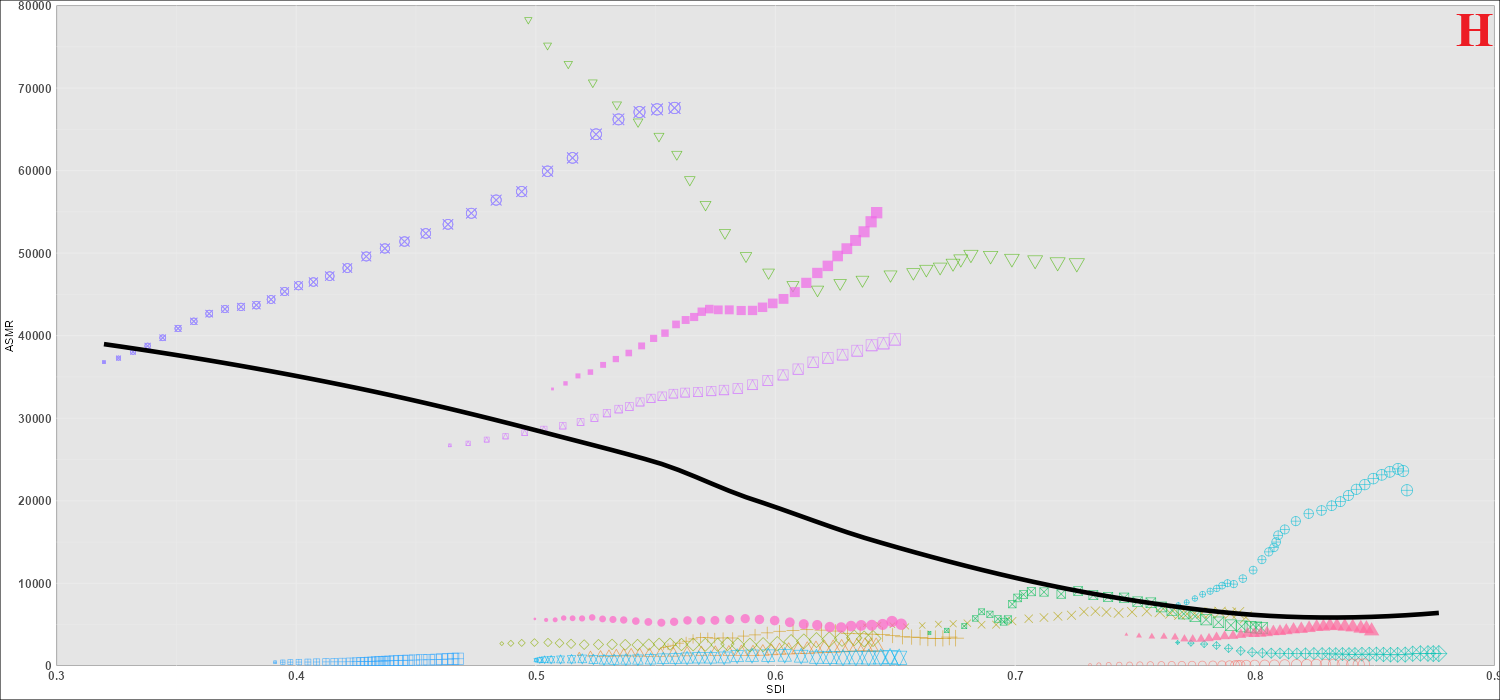


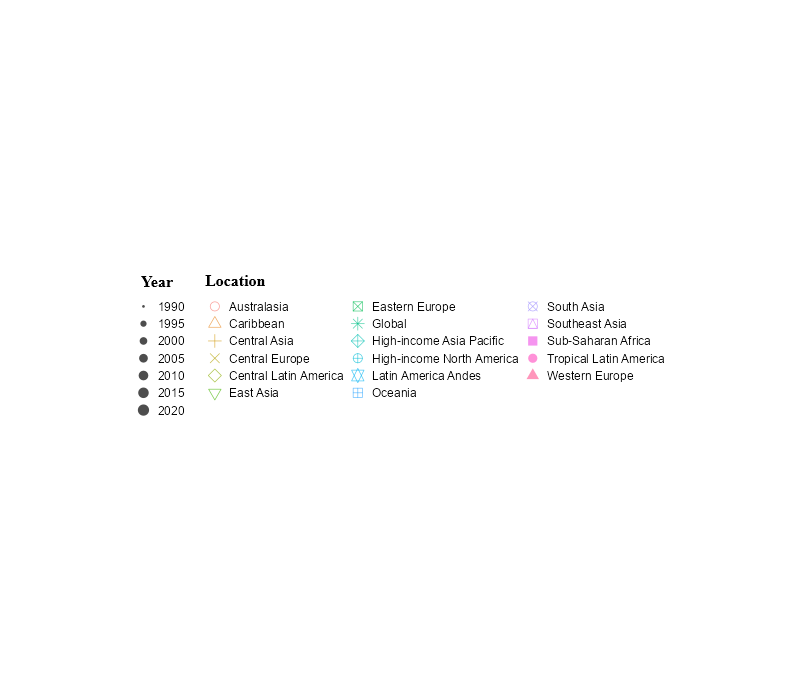


**Figure S4.ASDR caused by inadequate F&V intake in 17 GBD regions from 1990 to 2021**

(A. AA, C. PAD, E. IHD, G. HHD, attributed to insufficient intake of vegetables; B. AA, D. PAD, F. IHD, H. HHD, attributed to insufficient intake of fruits,All rates are expressed per 100 000 population.)

**
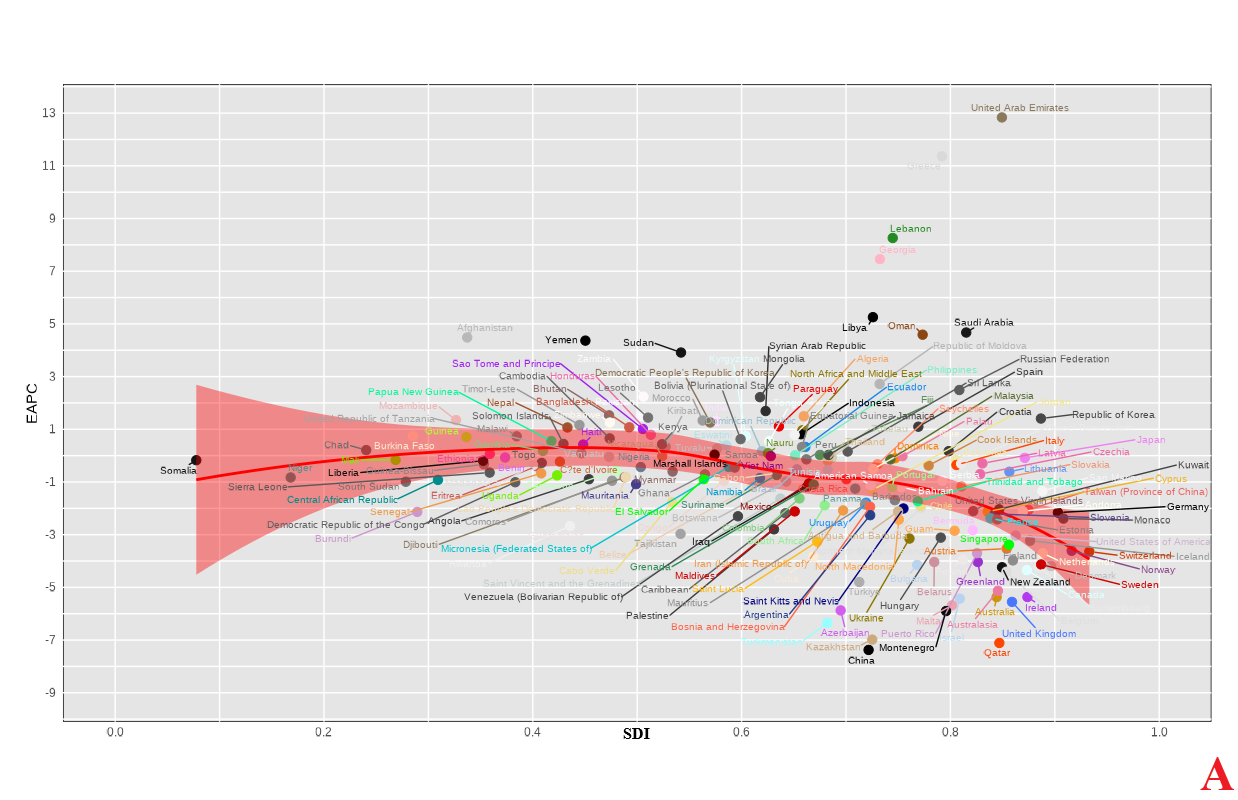

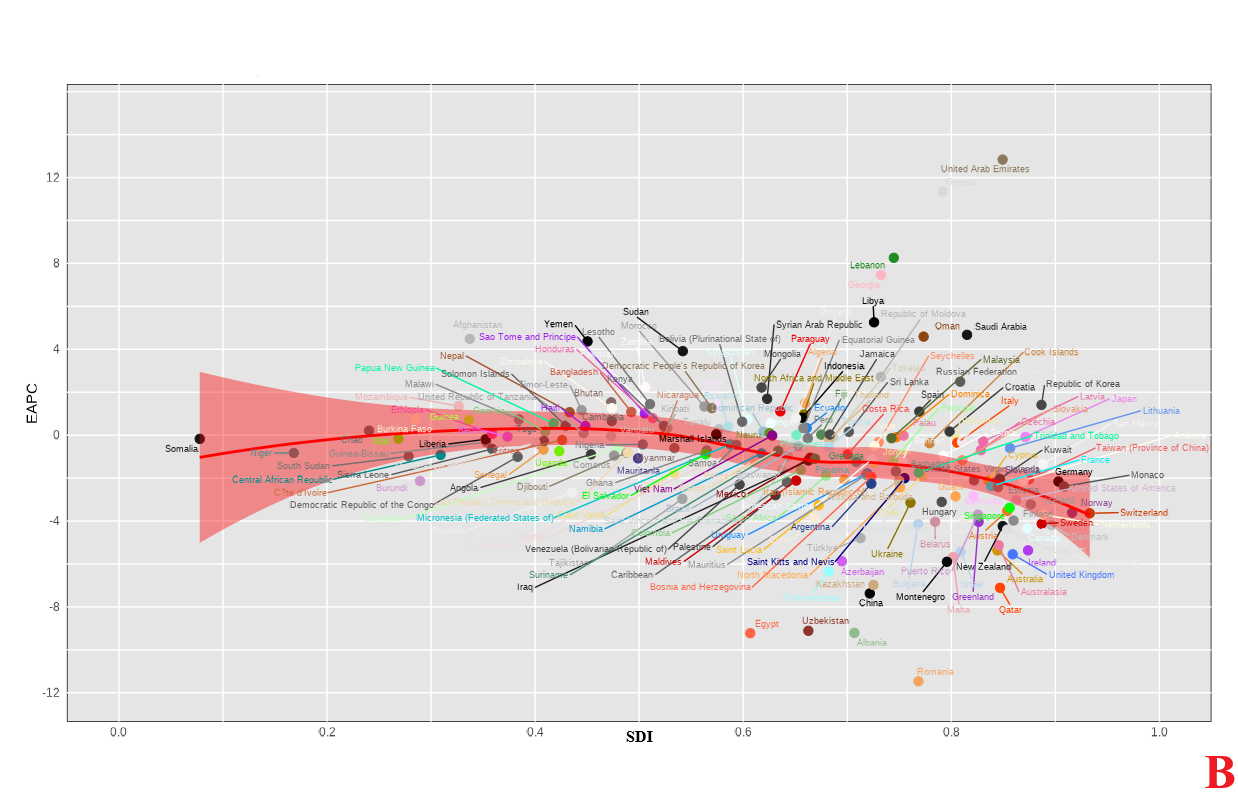
**

**
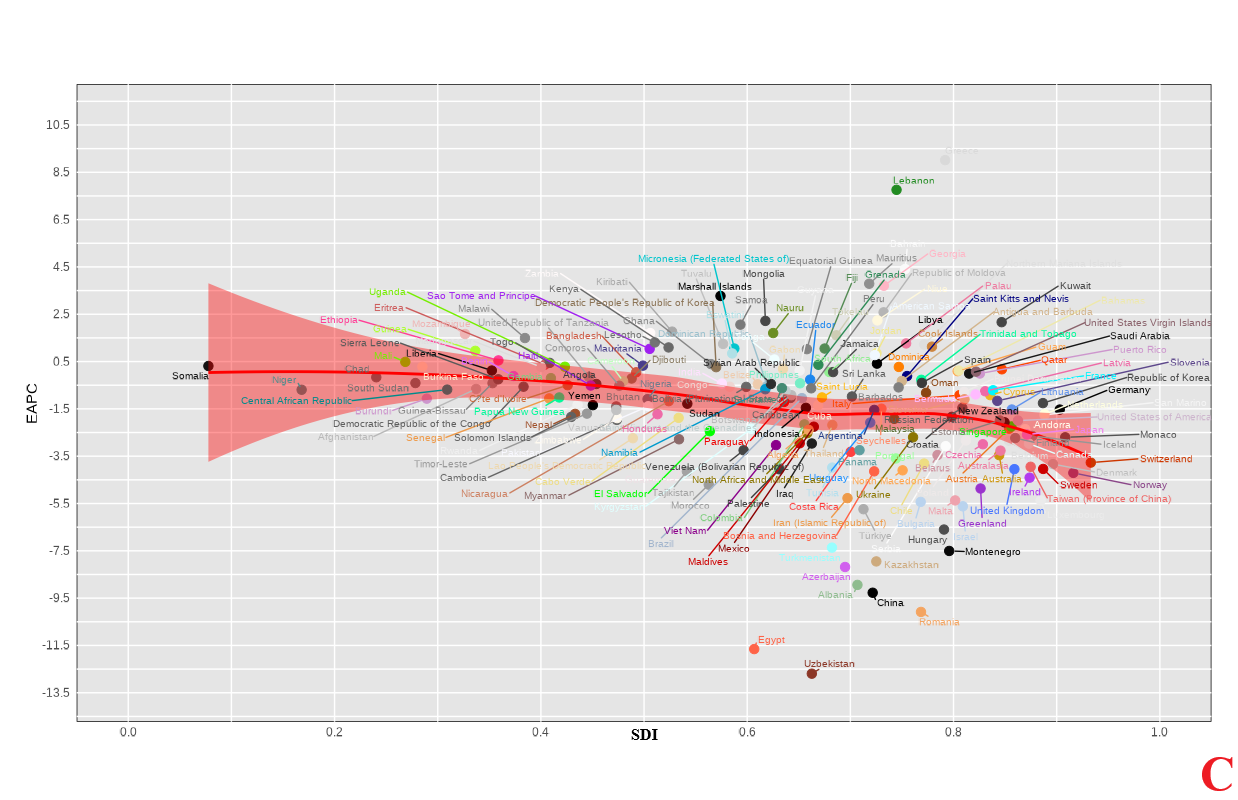

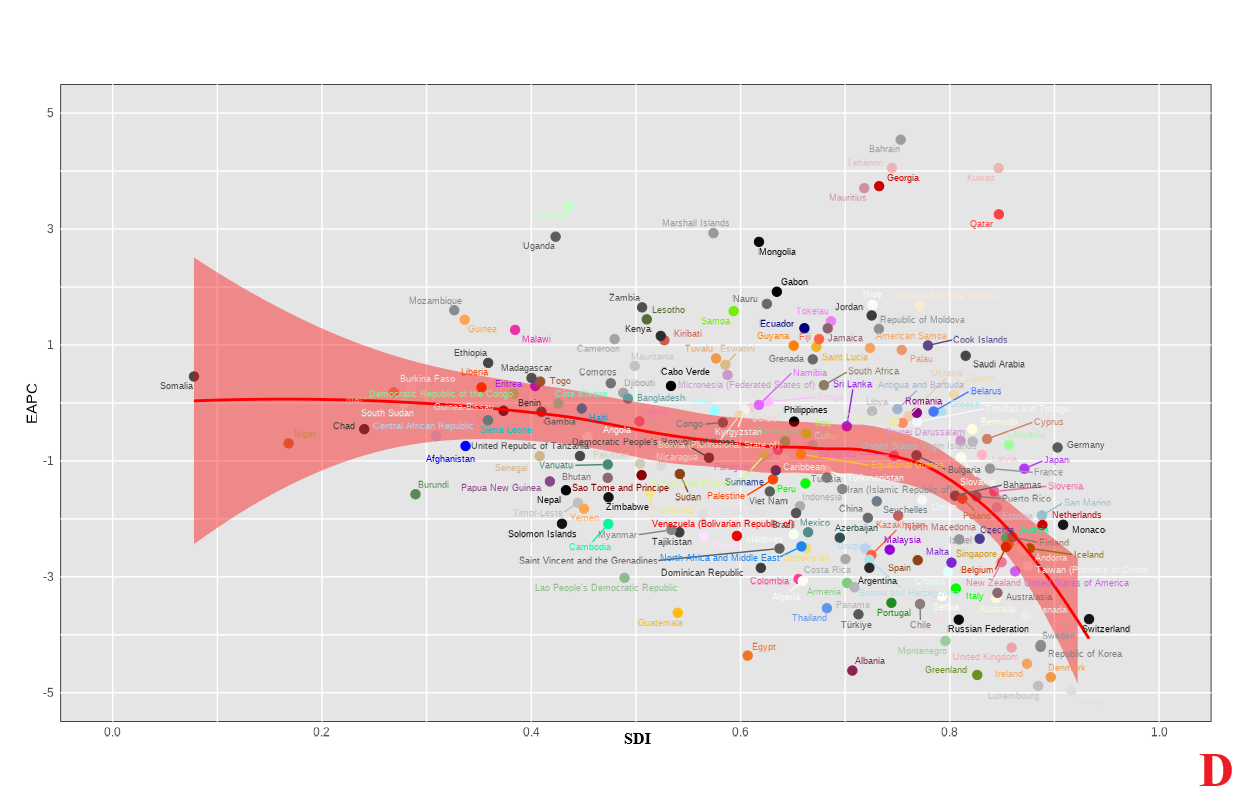
**

**
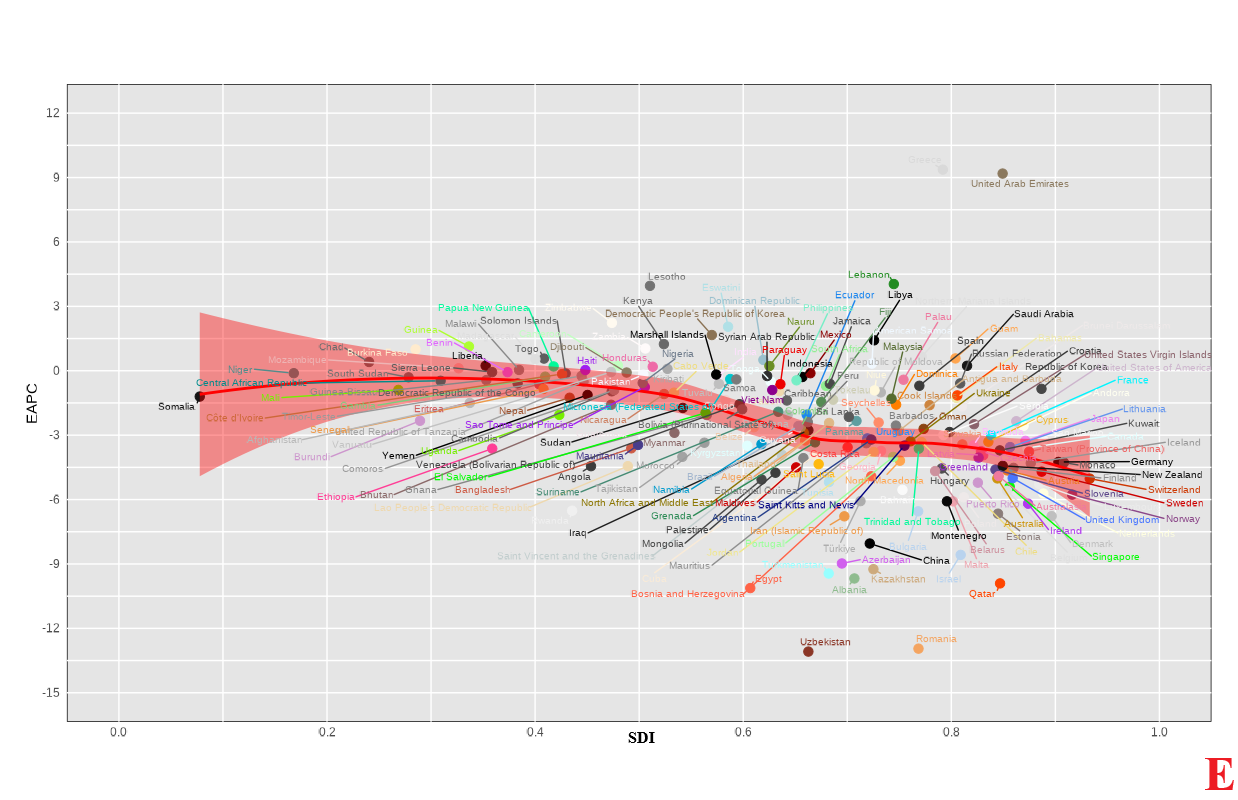

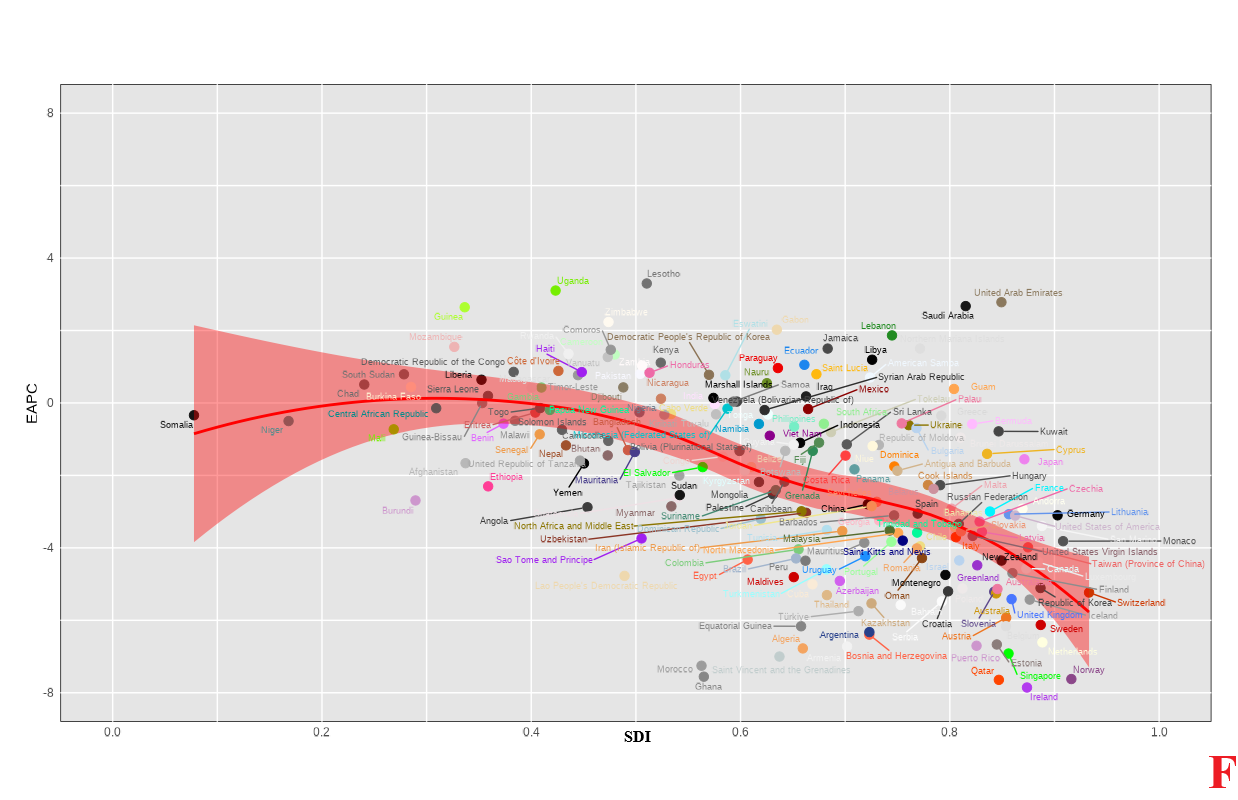
**

**
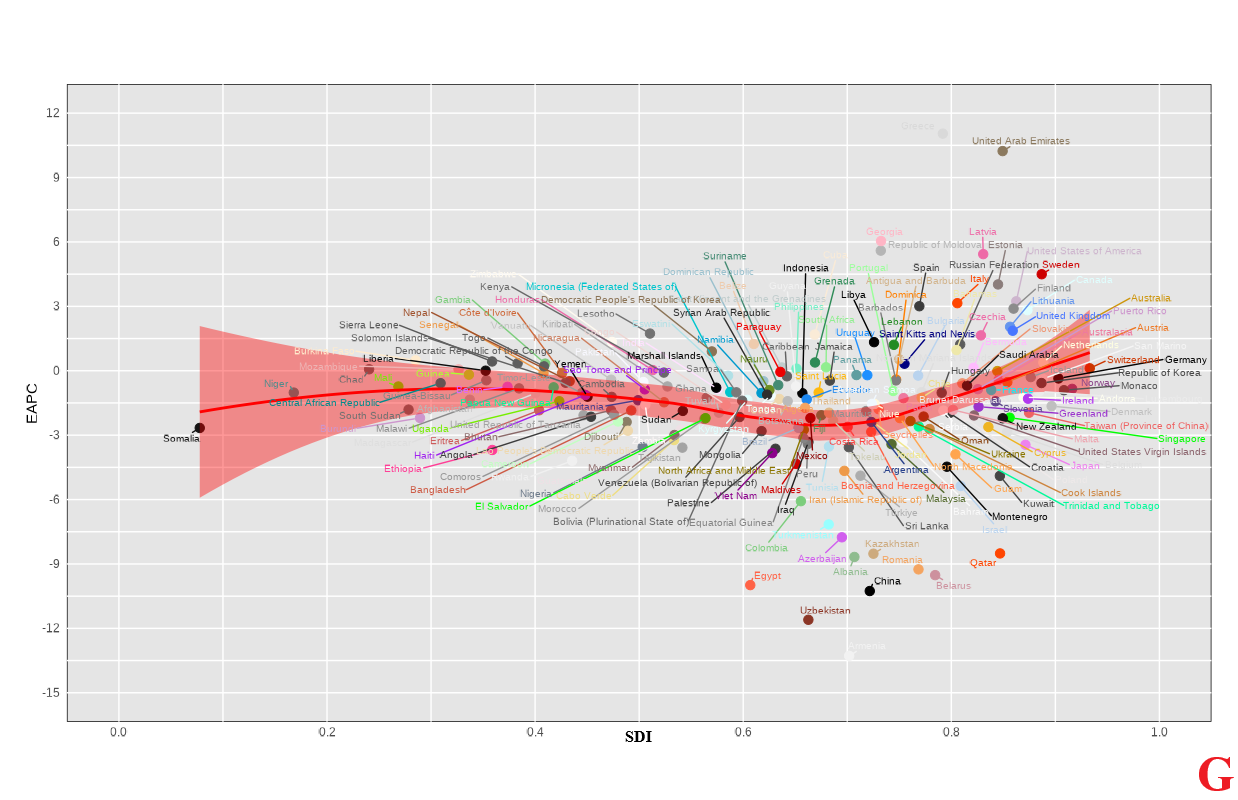

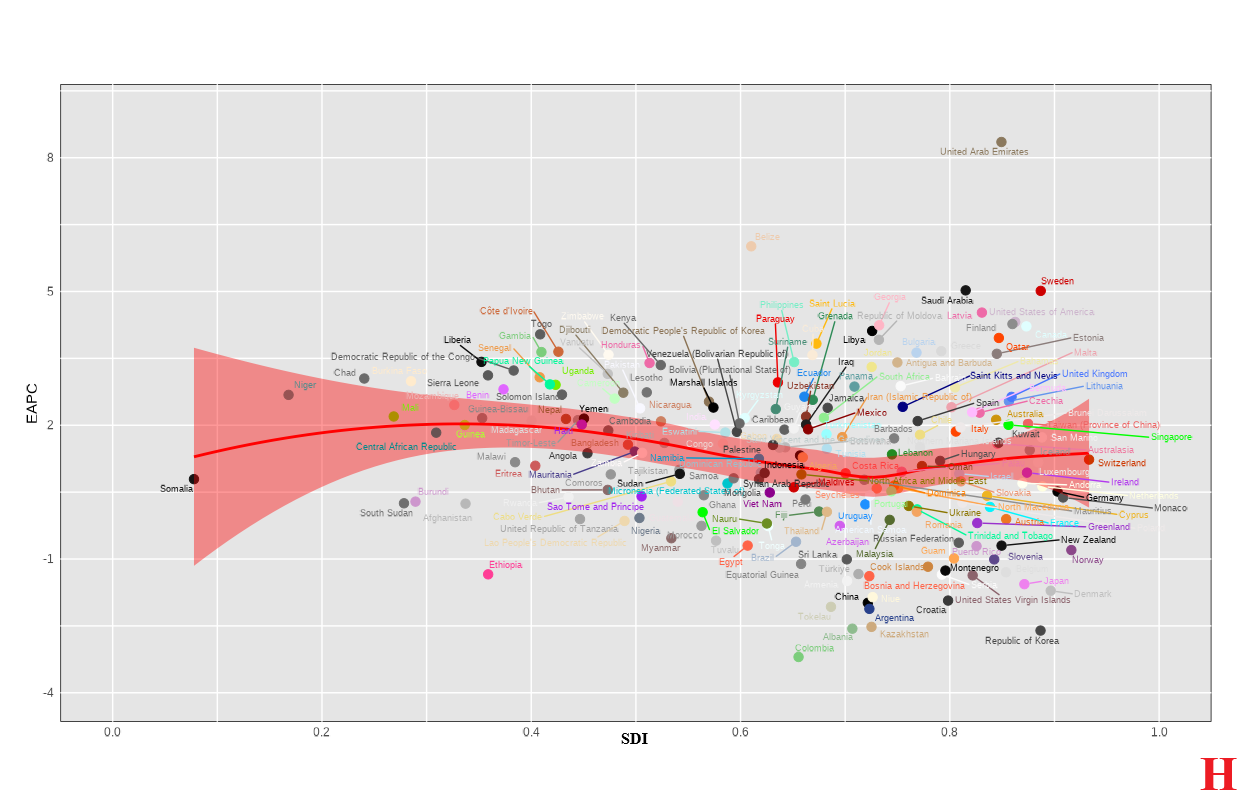
**

**Figure S5.Relationship between the global burden of AA caused by inadequate F&V intake and SDI in 204 countries and regions,EAPC of ASDR**

(A. AA, C. PAD, E. IHD, G. HHD, attributed to insufficient intake of vegetables; B. AA, D. PAD, F. IHD, H. HHD, attributed to insufficient intake of fruits)

**
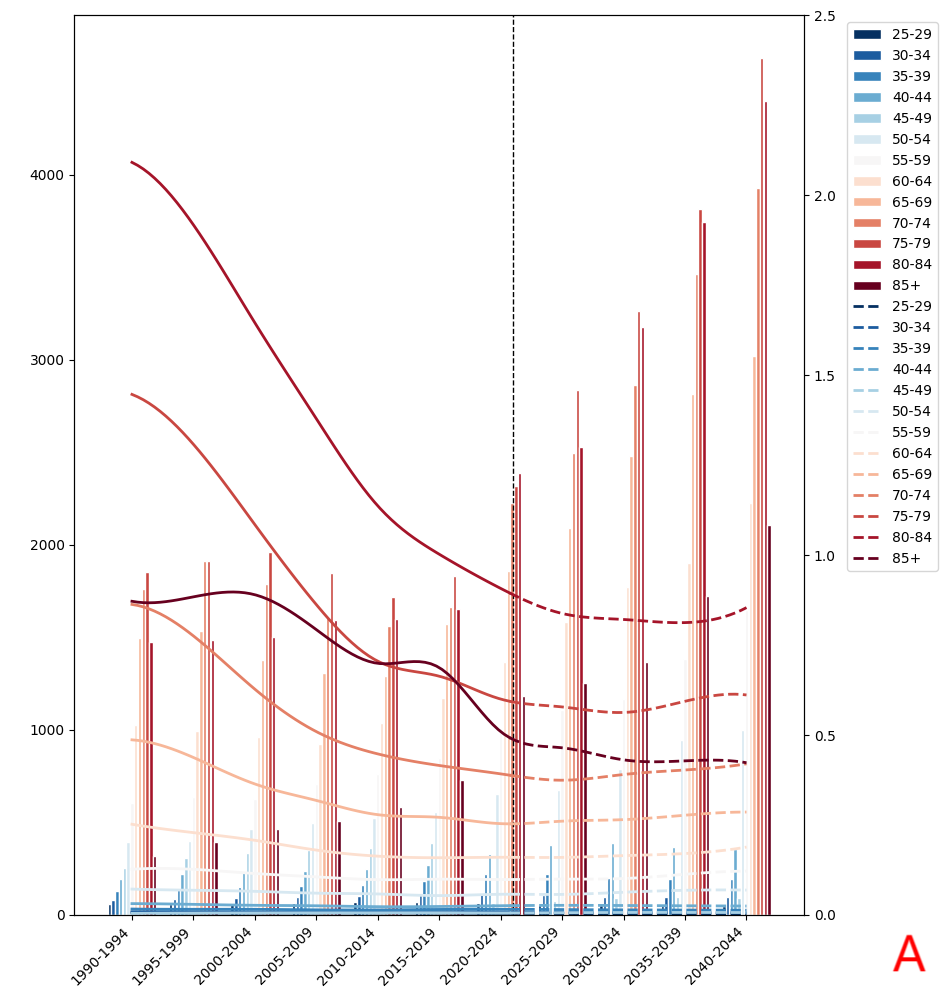

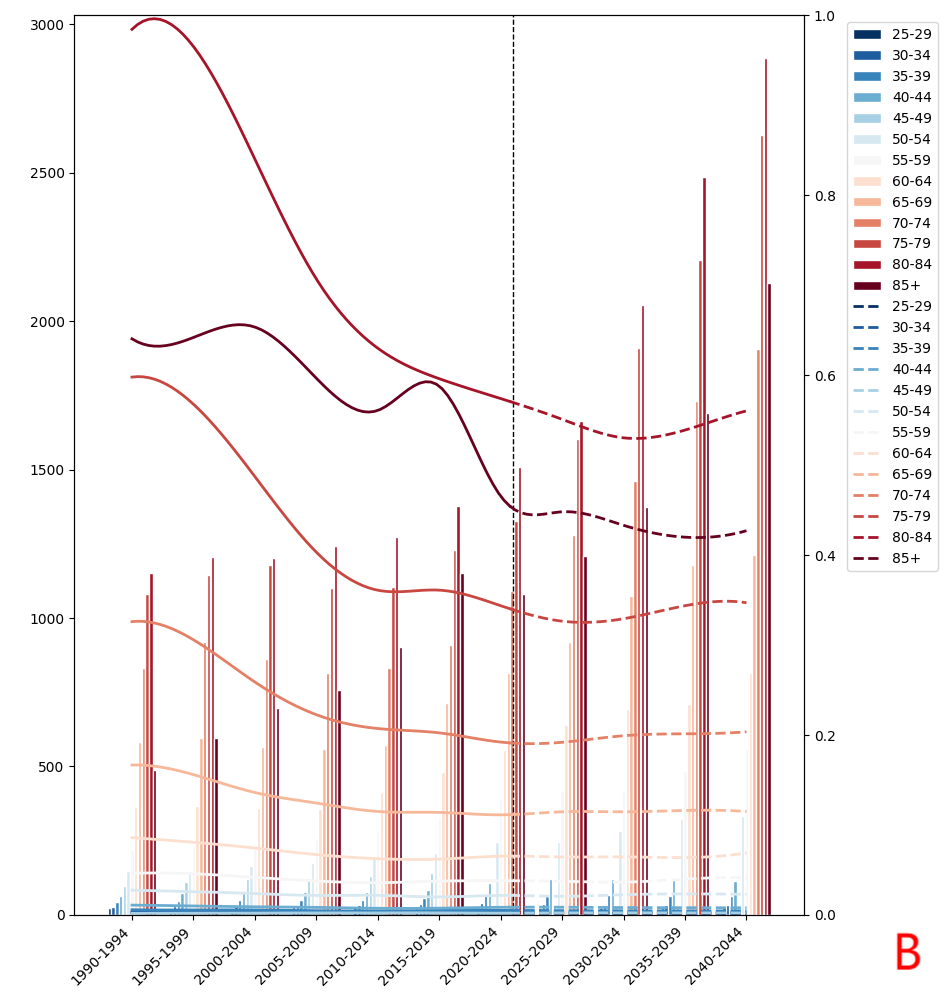
**

**
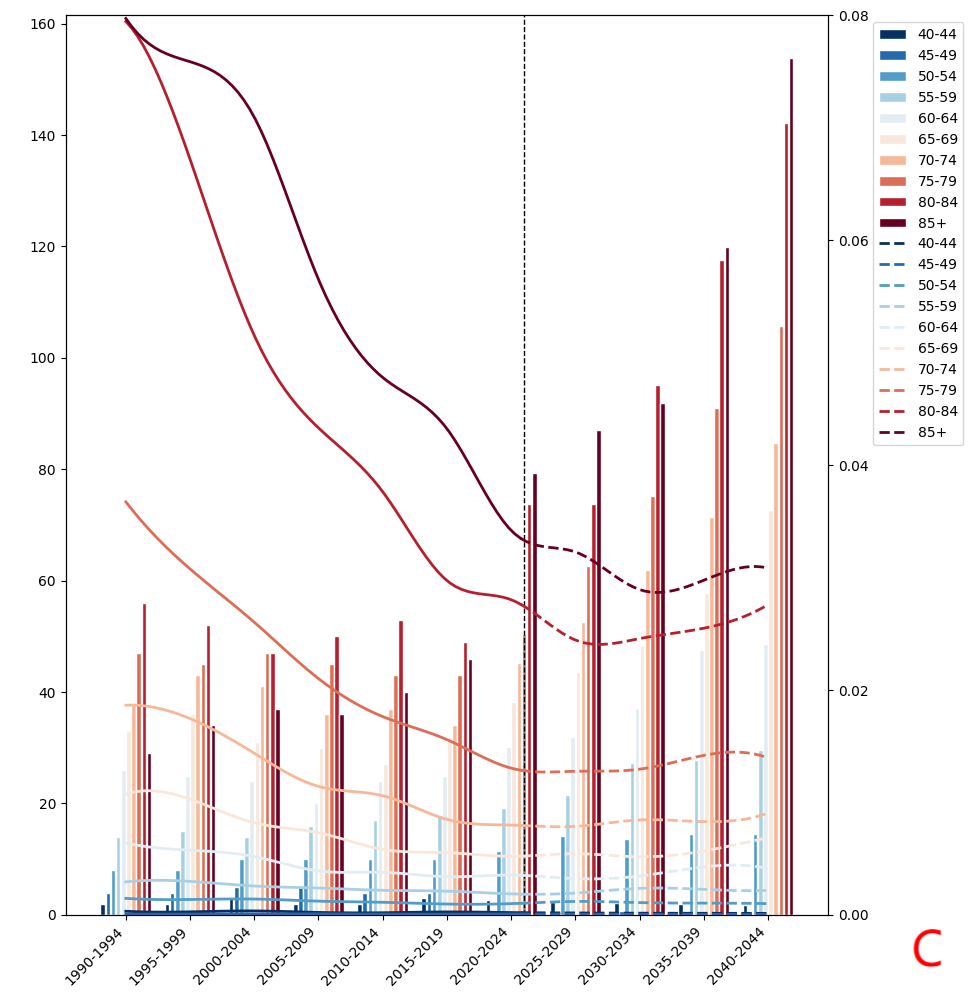

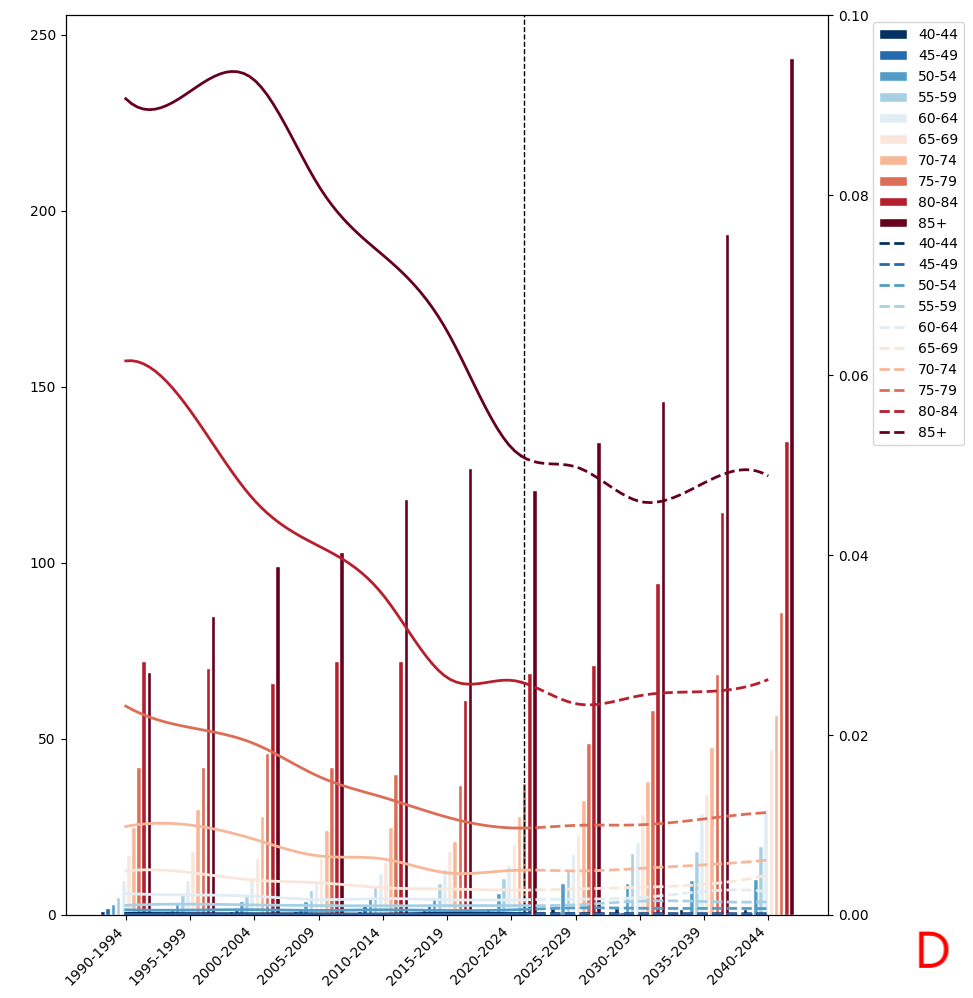
**

**
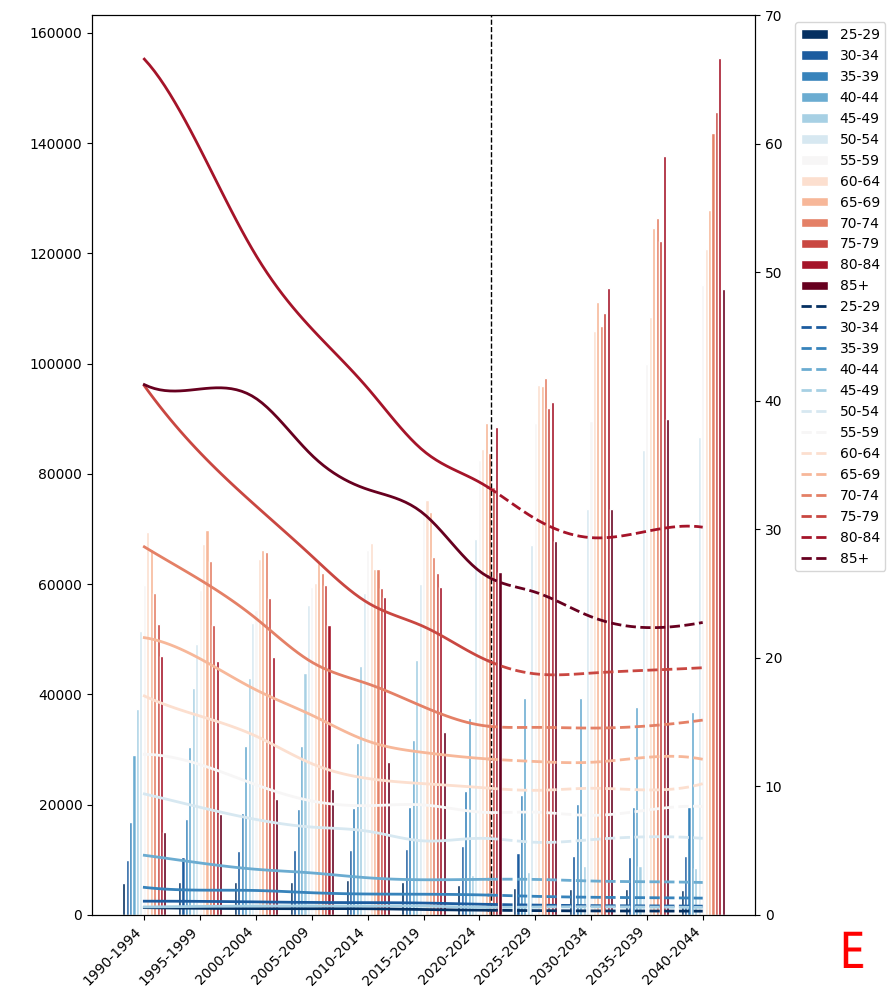

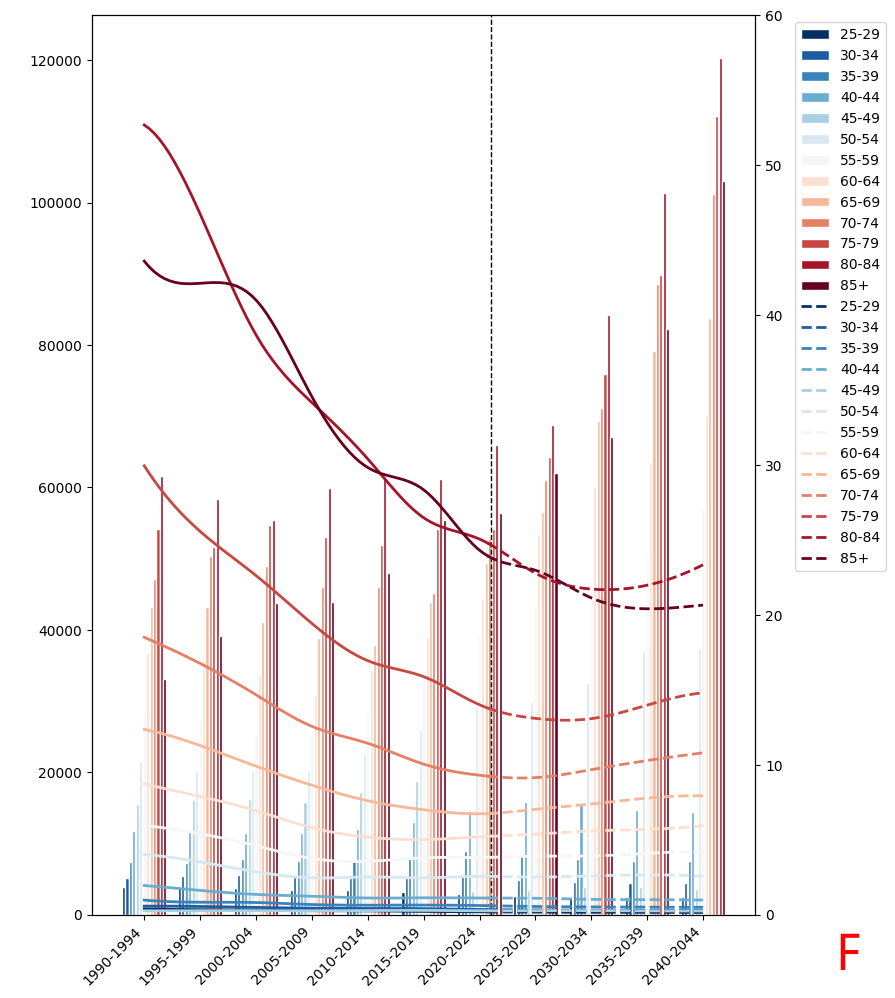

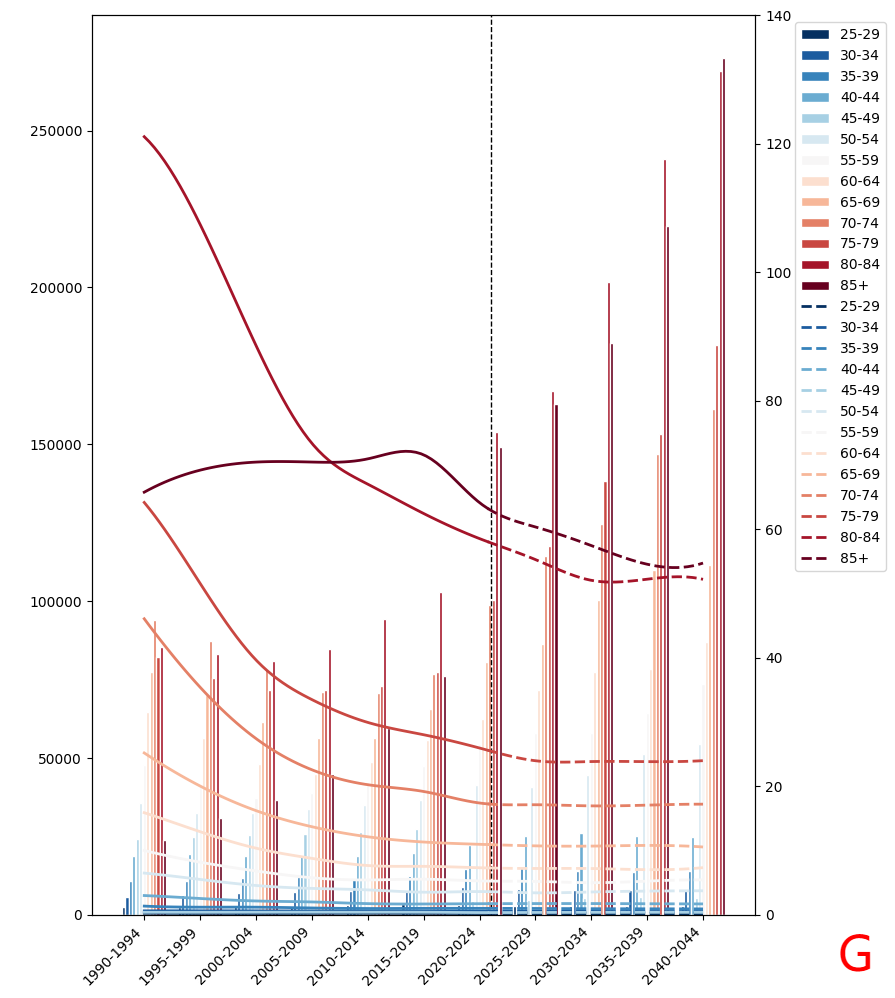

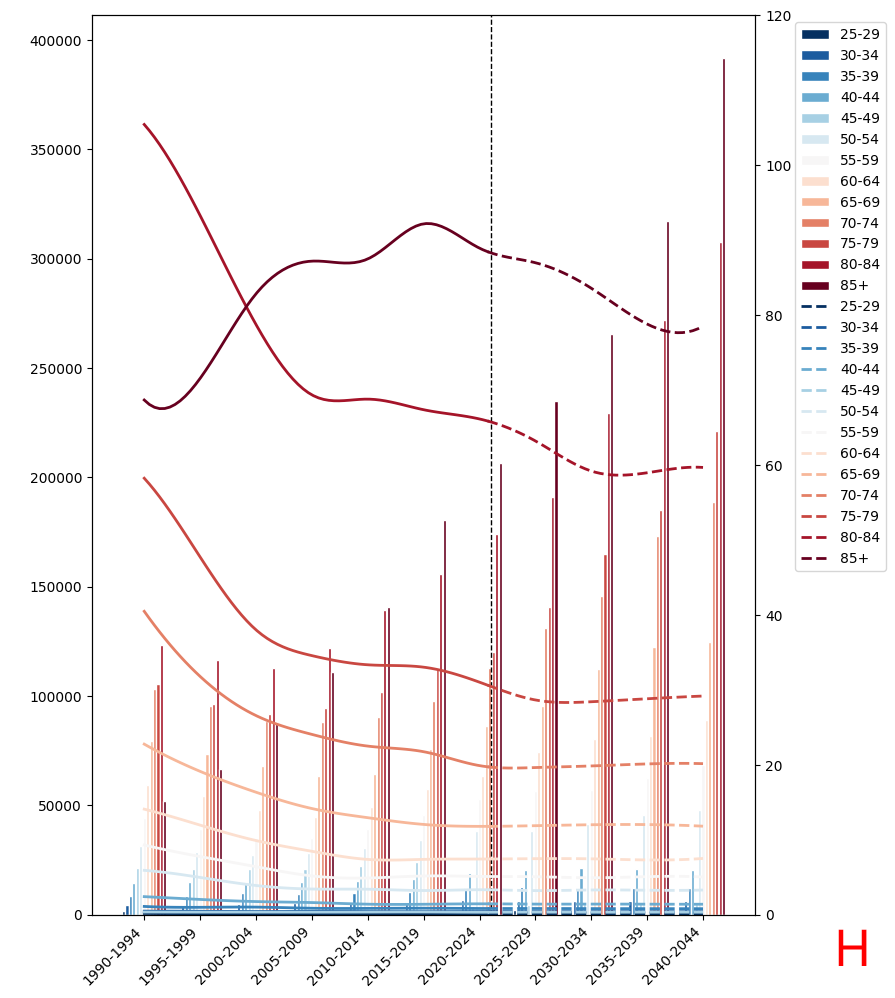
**

**Figure S6.Projected Mortality Numbers and Rates for Four CVDs Attributable to Insufficient vegetables Intake, Stratified by Gender, in Global by 2044**(A, B. Male/Female AA; C, D. Male/Female PAD; E, F. Male/Female IHD; G, H. Male/Female HHD attributed to insufficient vegetable intake. The line chart represents mortality rate per 100,000 people, while the bar chart represents the number of deaths.)

**
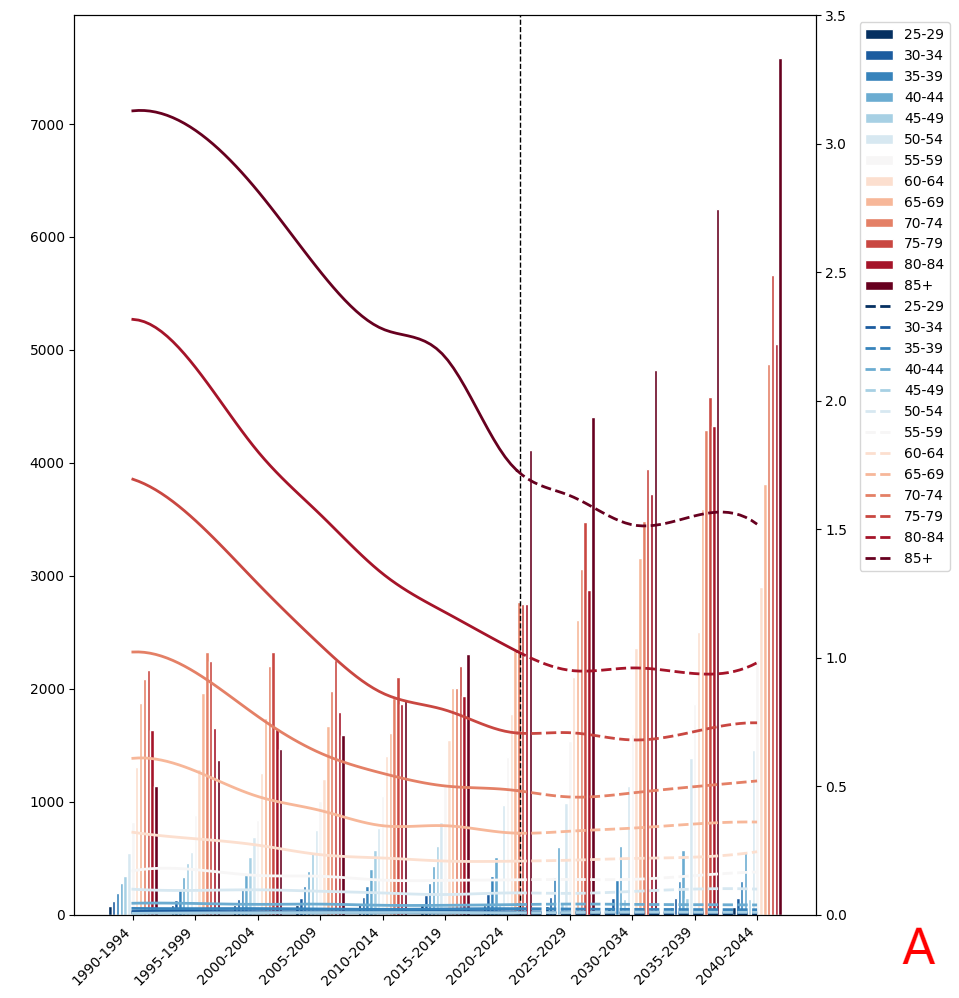

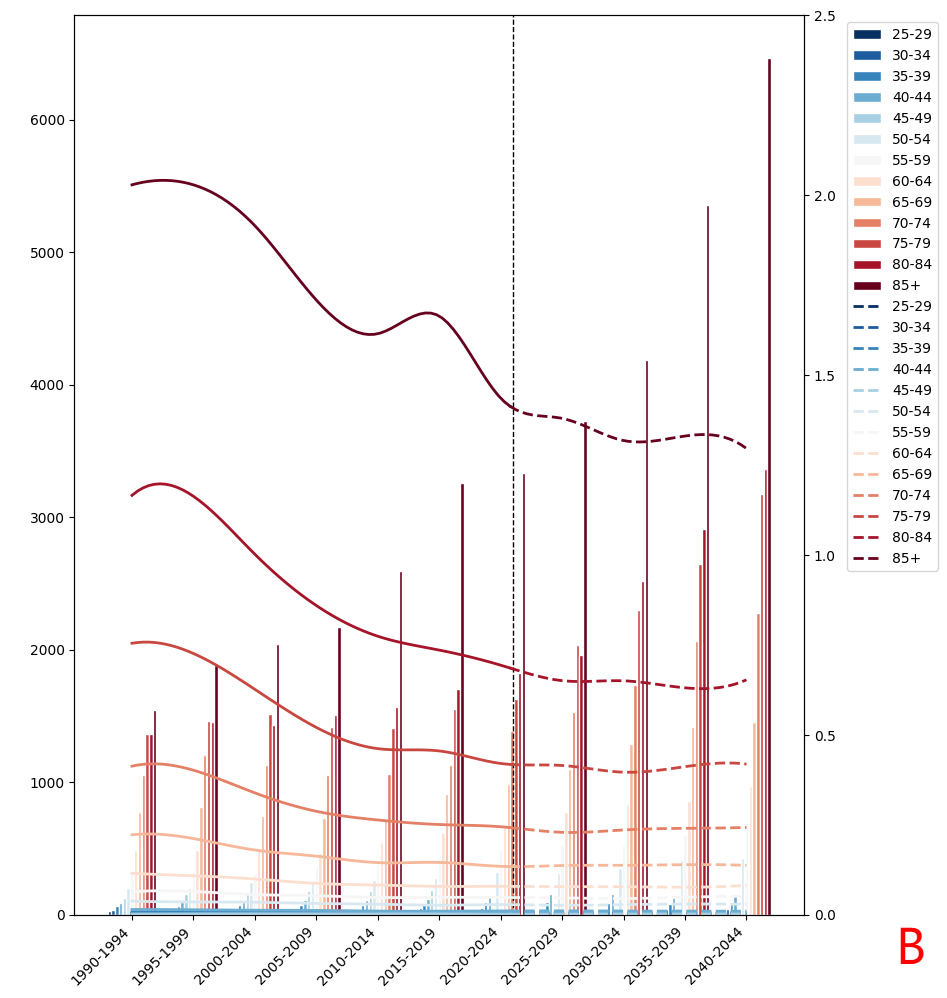
**

**
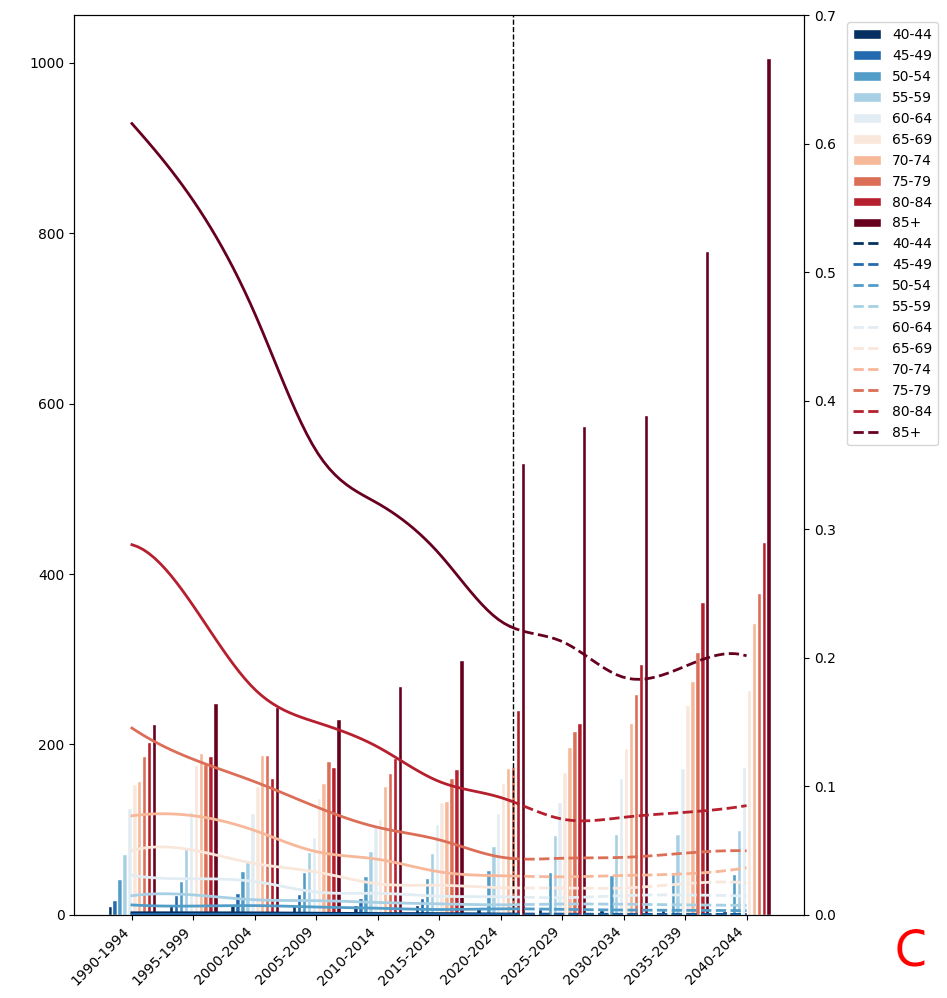

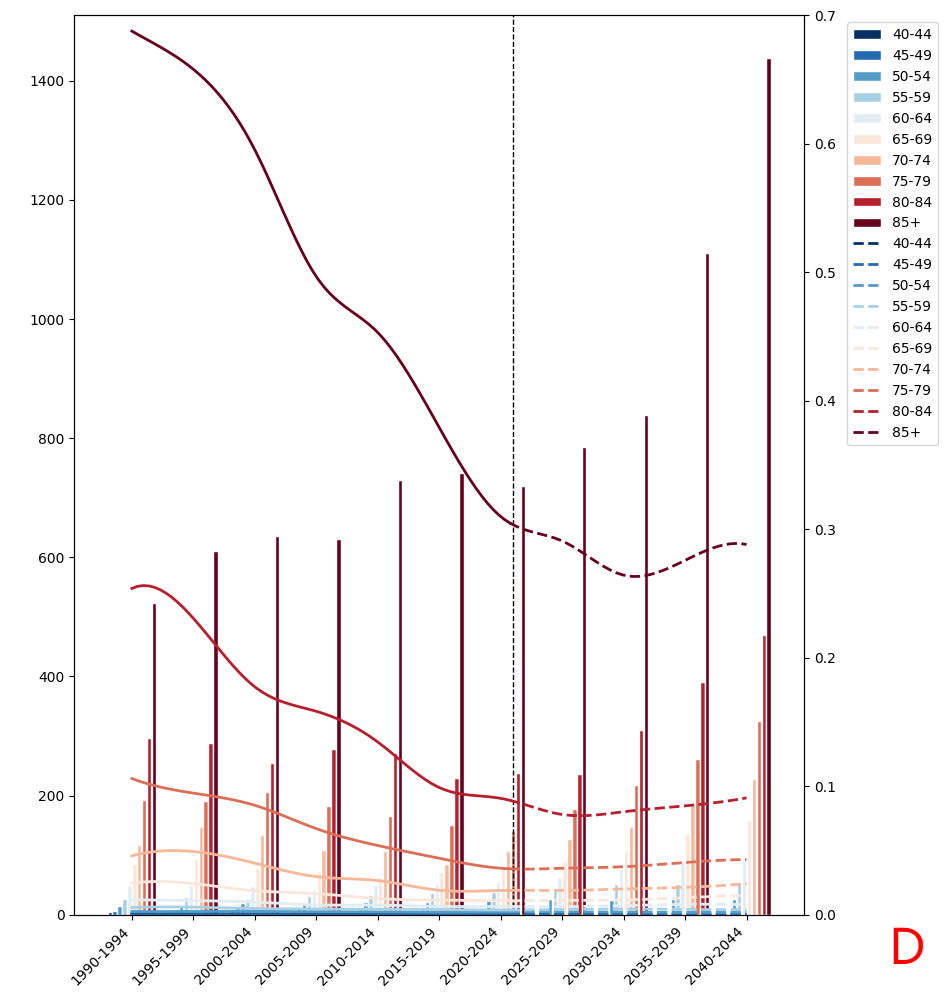
**

**
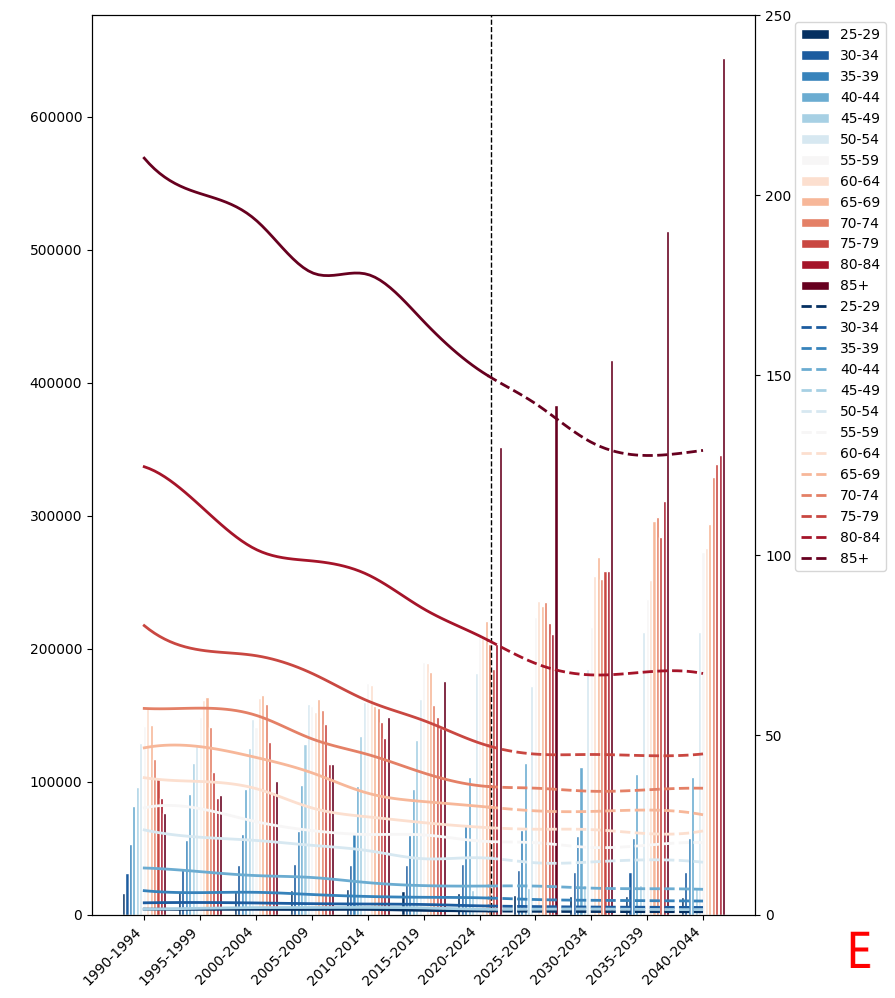

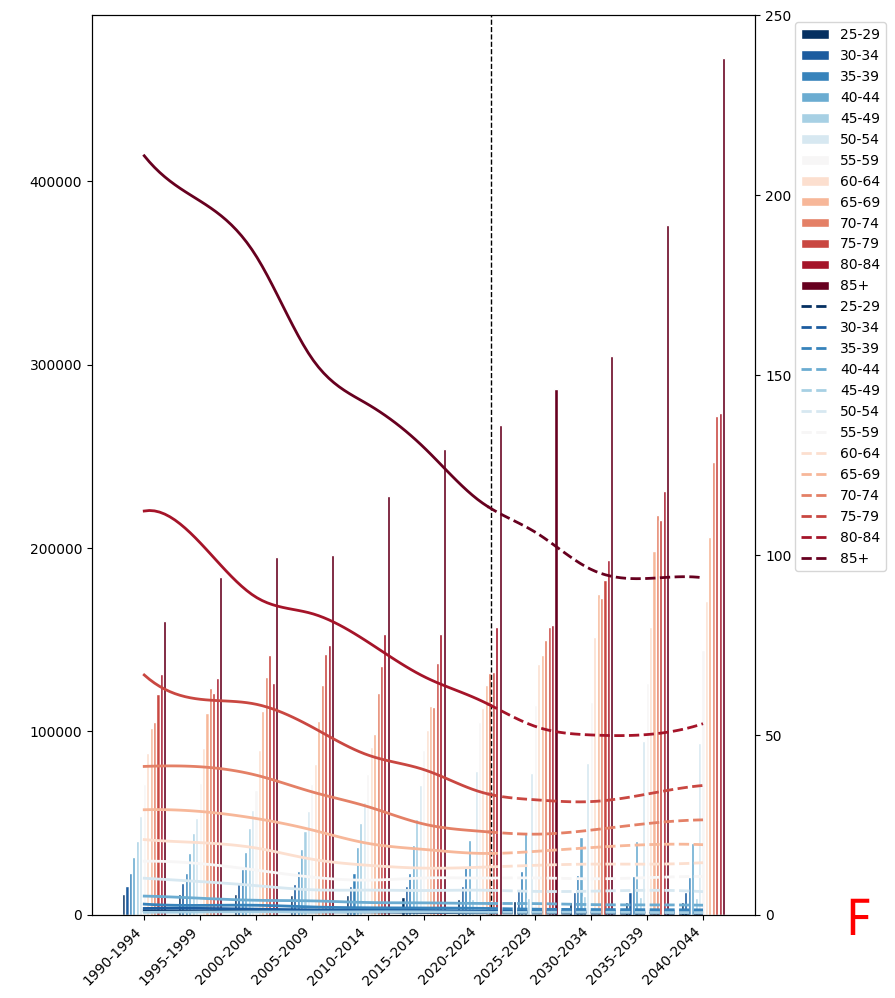
**

**
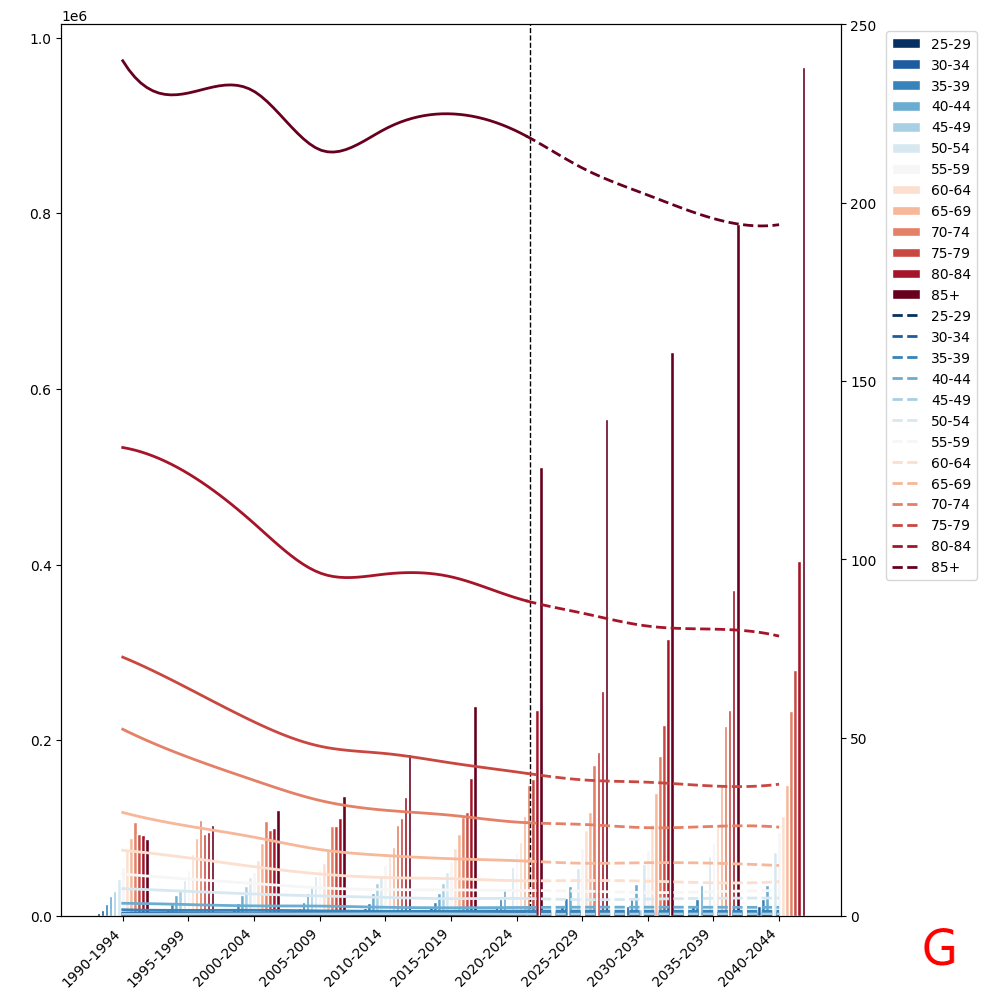
**
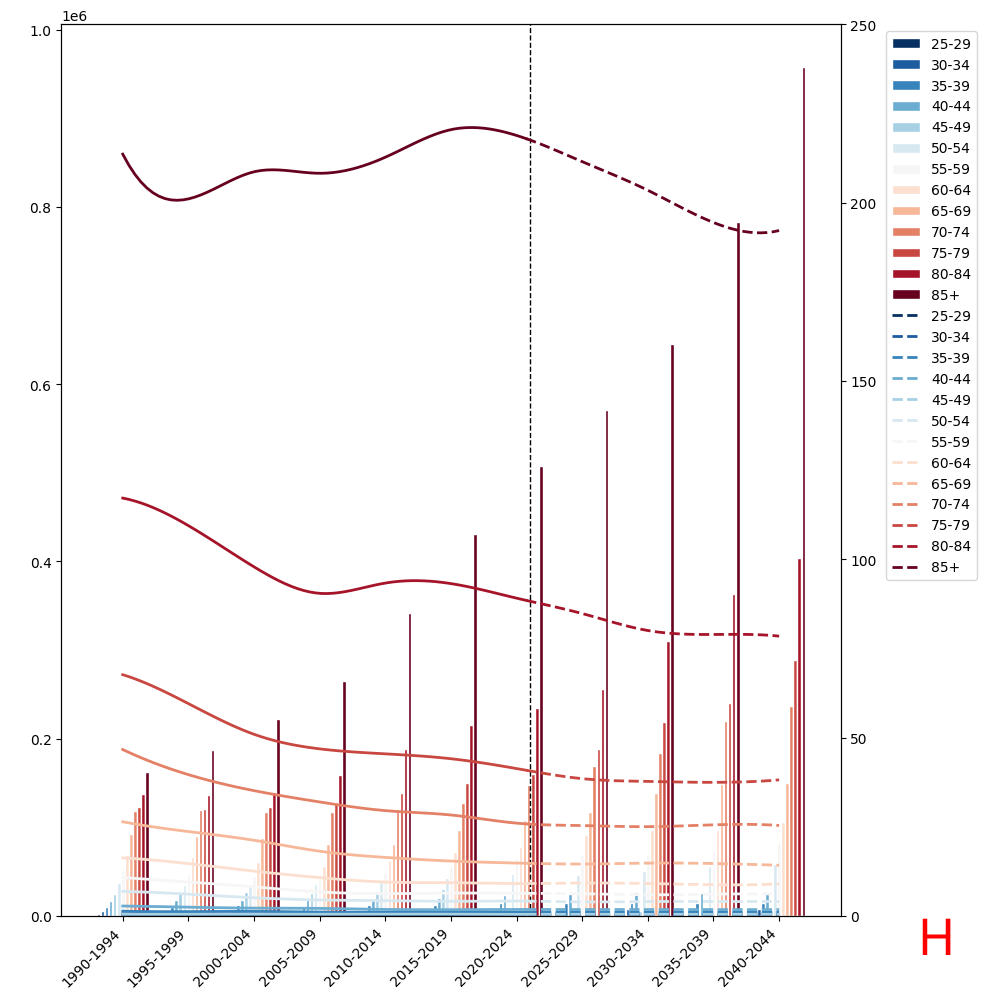


**Figure S7.Projected Mortality Numbers and Rates for Four CVDs Attributable to Insufficient vegetables Intake, Stratified by Gender, in Global by 2044**(A, B. Male/Female AA; C, D. Male/Female PAD; E, F. Male/Female IHD; G, H. Male/Female HHD attributed to insufficient fruit intake. The line chart represents mortality rate per 100,000 people, while the bar chart represents the number of deaths.)
